# Cross-Sectional Plasma Proteomic Phenotypes Associated with Prolonged Intermittent Levosimendan Exposure in Advanced Heart Failure

**DOI:** 10.1021/acsptsci.6c00195

**Published:** 2026-08-25

**Authors:** José Mesquita Bastos, Ines Cruz, Luis Santos, Rui Vitorino

**Affiliations:** † Serviço de Cardiologia da Unidade de Saúde Local Região Aveiro, Aveiro 3814-501, Portugal; ‡ Institute of Biomedicine (iBiMED), Department of Medical Sciences, University of Aveiro, Aveiro 3810-193, Portugal; § Cardiovascular R&D Centre − RISE, Department of Surgery and Physiology, Faculty of Medicine of the University of Porto, Porto 4200-319, Portugal

**Keywords:** Heart failure, Levosimendan, Proteomics, Biomarkers, Advanced therapy, Mass spectrometry

## Abstract

Levosimendan is intermittently administered in selected patients with advanced heart failure, but data on prolonged exposure and associated circulating molecular profiles remain limited. We conducted a retrospective observational study of 41 patients with heart failure with reduced ejection fraction, including eight patients exposed to ≥22 intermittent levosimendan infusions and 33 nonexposed controls followed in the same specialist heart failure setting. Baseline clinical characteristics, follow-up outcomes, and plasma proteomic profiles were assessed using label-free data-independent acquisition mass spectrometry. Patients exposed to prolonged intermittent levosimendan were older and had more advanced heart failure at baseline, including lower left ventricular ejection fraction and higher NT-proBNP concentrations. During clinical follow-up from the first specialist cardiology observation to plasma sampling, cardiovascular hospitalization occurred in 3/8 levosimendan-exposed patients and 1/33 controls, while cardiovascular death occurred in 2/8 and 0/33, respectively; these outcomes are reported descriptively because of baseline imbalance, retrospective treatment allocation, and the small exposed-group size. These outcomes are reported descriptively because of baseline imbalance and the small exposed-group size. Plasma proteomic analysis identified cross-sectional differences between groups. Control patients showed higher representation of immune and inflammatory proteins, whereas levosimendan-exposed patients showed higher abundance of proteins related to antioxidant defense, redox regulation, coagulation/hemostasis, and metabolic adaptation, including GPX3, SELENOP, PROS1, F2, AHSG, and ALDOA. These findings define an exploratory plasma proteomic phenotype associated with prolonged intermittent levosimendan exposure in real-world advanced heart failure. Prospective controlled studies with baseline and serial proteomic sampling are required to determine whether these molecular differences reflect treatment-associated adaptation or underlying clinical differences.

## Graphical abstract

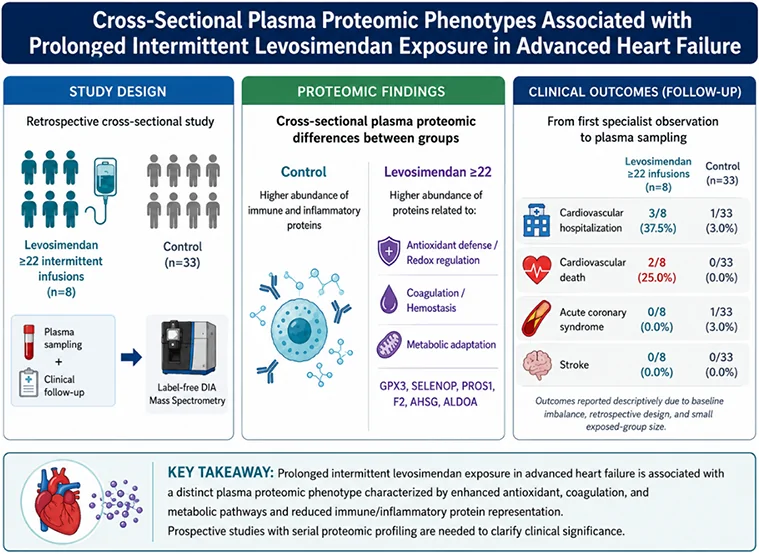

## Introduction

Heart failure (HF) affects an estimated 64 million people worldwide, with a prevalence of 1–3% and a lifetime risk approaching 25%.^1^ Despite therapeutic advances, prognosis remains poor, particularly among patients with advanced chronic HF (Stage D or New York Heart Association (NYHA) III–IV), where one-year mortality reaches 30–50%.^2^ These patients experience recurrent hospitalizations, worsening symptoms, loss of quality of life, and increasing healthcare costs, highlighting the need for innovative therapies that can provide sustained clinical stability.^3^ Levosimendan is a calcium-sensitizing inodilator with a unique dual mechanism of action: it enhances myocardial contractility without increasing intracellular calcium and is associated with vasodilation through ATP-sensitive potassium channel activation.^4^ It also opens mitochondrial K+ channels, conferring anti-ischemic and cytoprotective effects. Unlike catecholamine inotropes, levosimendan does not significantly increase oxygen consumption or proarrhythmic risk, making it an attractive option for advanced HF. Intermittent administration aims to improve hemodynamics and symptoms while avoiding the harmful effects seen with long-term adrenergic inotropes.^5^

Short-term randomized trials support these benefits. The LION-HEART (Levosimendan Intermittent Administration in Outpatients with Advanced Heart Failure) trial showed that biweekly levosimendan infusions over 12 weeks significantly reduced N-terminal pro-B-type natriuretic peptide (NT-proBNP) levels and lowered the risk of hospitalization for heart failure by approximately 75%.^6^ The LAICA (Long-Term Intermittent Administration of Levosimendan in Patients with Advanced Heart Failure) study evaluated monthly infusions for one year; although underpowered due to insufficient recruitment, it showed promising reductions in early decompensation and improved survival signals compared with placebo^6^. These findings suggest that intermittent levosimendan may provide clinical stabilization, yet evidence on long-term outcomes remains limited.^7^

Consequently, the use of levosimendan in chronic heart failure remains cautious. While approved in Europe for acute and chronic indications, it is recommended as a Class IIb option for selected end-stage patients, often as palliative support or a bridge to transplant or left ventricular assist device (LVAD).^8^ This cautious approach is due to an ongoing gap: there is insufficient robust long-term efficacy data and an incomplete understanding of the drug’s long-term molecular effects.

In addition to its hemodynamic profile, levosimendan demonstrates anti-inflammatory, antiapoptotic, and antioxidant effects in experimental studies,^9^ indicating possible disease-modifying properties. However, the systemic biological consequences of repeated exposure in advanced HF patients have not been comprehensively explored. No clinical studies have characterized the chronic plasma proteomic changes associated with extended levosimendan therapy, indicating a significant gap in mechanistic understanding.

Proteomics offers a robust framework to address this gap. Modern high-throughput, unbiased proteomic profiling enables a comprehensive examination of circulating proteins and pathways altered by cardiometabolic therapies.^10^ Such molecular insights may clarify mechanisms of action, identify biomarkers of response, and inform patient selection. In this context, the present study assessed the long-term clinical and molecular effects of intermittent levosimendan in patients with advanced heart failure administered in a day hospital. We examined a cohort undergoing more than 22 cycles of therapy, once every 2 weeks, during extended follow-up (1 year), correlating clinical outcomes with data-independent acquisition (DIA) plasma proteomics. We aimed to delineate the systemic molecular changes associated with prolonged levosimendan exposure and to provide new evidence regarding its potential long-term benefits in advanced heart failure.

## Materials and Methods

### Study Design and Population

Baseline was defined as the clinical visit immediately before the first levosimendan infusion in exposed patients. For controls, baseline was defined as an analogous specialist heart failure visit during the same care period. Plasma samples were collected later during routine follow-up and were not temporally synchronized with baseline clinical assessment. Therefore, baseline clinical variables and proteomic measurements represent different time points. This retrospective, observational cohort study included patients with advanced heart failure followed at a tertiary care center between January 2020 and December 2023 under part of the “HTelescan Project” approved by the Ethics Committee (ref. 2404–2022, April 24, 2022). There were patients referred to the day hospital. The eligibility criteria include patients, who had reduced left ventricular ejection fraction (LVEF < 40%) and persistent symptoms despite optimized guideline-directed medical therapy. Patients were categorized into two groups based on levosimendan exposure on top of modifying prognostic therapy tolerated by the patient: (1) a treatment group receiving ≥22 intermittent levosimendan infusions, and (2) a control group (Cont) with no levosimendan therapy but with modifying prognostic therapy tolerated by the patient. All exposed patients met clinical indications for intermittent inotropic support: refractory congestion, end-organ hypoperfusion, or documented intolerance to neurohormonal agents. The control group consisted of patients with advanced heart failure who were concurrently monitored at the same tertiary care center, using similar inclusion criteria (LVEF < 40%, persistent symptoms despite guideline-directed medical therapy) but to whom were not given levosimendan because (advance in a more hemodynamic status or had a contraindication to the drug (advance renal disease, hepatic dysfunction, severe hypotension) or by physician discretion. None of the patients in the Cont group had previously received levosimendan. Given the limited number of patients receiving prolonged intermittent levosimendan (n = 8), formal propensity score matching or multivariable adjustment was not performed, as such approaches are statistically unstable in very small samples and may introduce bias. The analysis therefore follows a descriptive, exploratory design reflective of real-world clinical practice.

### Levosimendan Administration

Levosimendan was administered intravenously over 6 h without a bolus, at a continuous infusion rate of 0.1 μg/kg/min. Infusions were generally repeated every 2 weeks. In selected clinically stable patients, based on symptom trajectory and New York Heart Association (NYHA) functional class, the interval could be extended to up to 4 weeks; however, this occurred infrequently. All patients in the levosimendan-exposed group completed at least 22 intermittent infusions during the follow-up period. This threshold was reached before plasma collection for proteomic analysis; therefore, the proteomic assay captured a cross-sectional molecular profile after prolonged levosimendan exposure rather than an early or acute treatment response. Therefore, receipt of ≥22 infusions was used to define prolonged intermittent levosimendan exposure. All levosimendan infusions were administered in a monitored hospital day-care setting under specialist heart failure supervision.

### Clinical and Laboratory Assessments

Baseline and follow-up data were extracted from electronic health records, including demographic variables, comorbidities, medication profiles, echocardiographic parameters, and laboratory values. The primary clinical outcomes were New York Heart Association (NYHA) functional class, left ventricular ejection fraction (LVEF), N-terminal pro-B-type natriuretic peptide (NT-proBNP).

Secondary variables included systolic and diastolic blood pressure, heart rate, serum creatinine, urea, hemoglobin, and platelet count. Cardiovascular hospitalizations and mortality outcomes were tracked during follow-up. For postsampling outcome ascertainment, follow-up was defined as the interval between the date of blood/plasma collection for proteomic analysis and the last available clinical contact or electronic health-record review [insert date]. Cardiovascular hospitalization and mortality occurring during this interval were extracted from the electronic health records.

### Plasma Proteomic Profiling

Plasma sampling was performed at follow-up visits in sessions for repeated levosimendan (Levos) infusion. Blood samples were collected in EDTA tubes and centrifuged at 2,000 × g for 10 min at 4 °C to obtain plasma. The plasma fraction was aliquoted and stored at −80 °C until proteomic analysis. Total protein extracts were obtained directly from plasma samples without depletion of high-abundance proteins. Plasma sampling was performed during routine follow-up visits, typically approximately 15 days after the last levosimendan infusion, and not at a standardized pharmacodynamic point. Plasma protein quality was initially assessed by one-dimensional SDS–PAGE. For this, 0.5 μL of each total protein extract was loaded onto 12% polyacrylamide gels, which were then stained with Coomassie Brilliant Blue G-250 (CBB-G250). Gel images were examined to confirm protein integrity and consistency between samples.

Gel lanes were stained, excised, and subjected to in-gel digestion. Proteins were reduced with dithiothreitol (DTT) and subsequently alkylated with iodoacetamide (IAA) prior to trypsin digestion. Protein digestion was carried out using a filter-assisted sample preparation (FASP) workflow. Approximately 50 μg of solubilized protein from each plasma sample was loaded onto 30 kDa molecular-weight cutoff filters. Proteins were reduced and alkylated with iodoacetamide, then digested with sequencing-grade trypsin for 18 h at 37 °C. The resulting peptides were recovered from the filters and prepared for liquid chromatography–mass spectrometry (LC–MS) analysis. Prior to DIA, peptide-level quality Cont was performed using an RSLCnano system coupled to a Bruker Impact II mass spectrometer to verify digestion efficiency, peptide yield, and sample complexity. Comprehensive proteomic profiling was conducted by LC–MS using an RSLCnano system interfaced with a Thermo Scientific Exploris 480 Orbitrap mass spectrometer. Peptides were separated using a 90 min reversed-phase gradient on a nanoLC system equipped with a C18 analytical column (e.g., 75 μm × 25 cm, 2 μm particle size, 100 Å pore size) maintained at 40 °C. Mobile phases consisted of 0.1% formic acid in water (A) and 0.1% formic acid in acetonitrile (B). Peptides were eluted using a linear gradient from 5% to 35% B over 90 min at a flow rate of 300 nL/min. Full MS scans were acquired in the Orbitrap at 120,000 resolution (*m*/*z* 200), and MS/MS spectra were acquired in the Orbitrap at 15,000 resolutions after HCD fragmentation. Data-independent acquisition (DIA) was performed across the *m*/*z* 400–800 precursor range. All peptide mixtures were analyzed individually, and 1000 ng of peptide was injected for each run based on the concentration determined during peptide QC.

DIA raw files were processed using DIA-NN (version 1.9.2) in library-free mode. Searches were performed against the UniProtKB human reference proteome (taxonomy: *Homo sapiens*, taxon ID 9606; release 27-11-2024; 20,650 sequences) and an iRT_trypsin calibrant library containing 12 sequences. Match-between-runs (MBR) was enabled and applied separately to each biological subset. False discovery rates were controlled at 1% at both the precursor and protein group levels. DIA-NN’s default protein inference algorithm was used to assemble protein groups exclusively from proteotypic peptides.

### Statistical and Multivariate Analysis

All statistical analyses were performed using MetaboAnalyst 6.0 (www.metaboanalyst.ca). Protein intensity values were log2-transformed and autoscaled, and missing values were imputed using a k-nearest neighbors (KNN) approach only after stringent filtering (proteins with <20% missing values). Imputation was applied exclusively for exploratory multivariate visualization and functional enrichment analyses and was not used to support inferential statistics, prediction, or causal interpretation. Group comparisons were conducted using unpaired *t* tests or Mann–Whitney U tests, depending on data normality assessed by the Shapiro–Wilk test. Multiple hypothesis testing was corrected using the Benjamini–Hochberg method, with statistical significance defined as a false discovery rate (FDR) < 0.05. Multivariate analyses included Partial Least Squares–Discriminant Analysis (PLS-DA), hierarchical clustering analysis (HCA) using Euclidean distance and heatmap visualization of Z-score–normalized protein expression profiles. Given the limited sample size, PLS-DA results were not used for prediction or inferential statistics. All statistical outputs and graphical representations were generated within the MetaboAnalyst environment.

### Bioinformatics and Functional Enrichment

Differentially abundant proteins (FDR < 0.05) were further analyzed for functional significance using the ShyGO platform. Gene Ontology (GO) Biological Process terms were used as the annotation framework. Enrichment significance was quantified and ranked according to −log10­(FDR) and visualized using dot plots to highlight the most overrepresented biological pathways.

### Slot Blot Validation

We performed slot blot analysis to confirm the proteomic results. Using a slot blot apparatus under vacuum, equal amounts of plasma protein were applied to nitrocellulose membranes. After immobilization, the membranes were stained with Ponceau S to verify equal total protein loading. The membranes were then blocked with 5% nonfat dry milk in TBS-T for 1 h at room temperature and incubated overnight at 4 °C with antialdolase A primary antibody (AB150396, Anti-Aldolase antibody [EPR9725], Abcam), which has been validated for Western blotting with human samples. After washing, the membranes were incubated at room temperature for 1 h with HRP-conjugated secondary antibody. Chemiluminescence was used to detect signals, and densitometry was used for quantification. Total protein staining was used to normalize the ALDOA signal intensity. Given the absence of electrophoretic separation, slot blot does not resolve protein isoforms or potential comigrating species, and signal specificity depends entirely on antibody performance.

## Results

### Baseline Characteristics

We evaluated 41 patients with advanced HF; eight (19.5%) met the criterion for prolonged intermittent levosimendan exposure, and 33 (80.5%) served as Cont. In levosimendan-exposed patients, baseline clinical characteristics were recorded before the first levosimendan infusion. In control patients, baseline characteristics were recorded at an analogous reference visit during specialist heart failure follow-up. Plasma proteomic profiling was performed later during routine follow-up after prolonged therapy. Therefore, baseline clinical variables and molecular measurements represent different time points and should not be interpreted as temporally synchronized. Plasma proteomic profiling, however, was performed later during routine follow-up after prolonged therapy. Therefore, baseline clinical variables and molecular measurements represent different time points and should not be interpreted as temporally synchronized. Patients who received Levos were significantly older than Cont (75.8 ± 8.4 years vs 68.0 ± 10.0 years, p = 0.050). Age and sex distributions differed between groups, both of which may influence clinical status and circulating protein profiles; these baseline imbalances reflect real-world practice, where Levos is typically administered to older and more frail patients with advanced disease. These baseline imbalances reflect treatment allocation in routine care, where Levos is preferentially administered to older, more severely ill patients with limited therapeutic options.

As expected (Table 1), at baseline, prior to initiation of Levos therapy, the levosimendan-exposed group presented with more severe heart failure. Levos left ventricular ejection fraction (LVEF) was lower (24.5 ± 5.9% vs 34.3 ± 10.2%, p = 0.013), and worst NYHA functional class (*p* < 0.001). In the exposed group, systolic blood pressure was lower (102.2 ± 16.2 mmHg vs 115.9 ± 16.7 mmHg, p = 0.043). However, diastolic pressure and heart rate were similar between the two groups. Comorbidities such as hypertension, diabetes, dyslipidemia, obesity, atrial fibrillation, and chronic kidney disease were common across the cohort, though renal impairment was more pronounced among levosimendan-exposed patients (creatinine 2.1 ± 1.1 vs 1.4 ± 0.5 mg/dL, p = 0.004; urea 118.3 ± 106.3 vs 65.7 ± 25.3 mg/dL, p = 0.013).

The Levos group had much higher baseline NT-proBNP levels (6795.8 ± 5201.8 pg/mL vs 1782.9 ± 2445.9 pg/mL, *p* < 0.001), indicating more severe heart failure. Exposed patients also had lower levels of hemoglobin and platelets (both *p* < 0.05). The use of guideline-directed medical therapy was generally high in both groups, including β-blockers, ACEI/ARB, and ARNI; however, the use of mineralocorticoid receptor antagonists (MRAs) was significantly lower among levosimendan-exposed patients (37.5% vs 84.8%, p = 0.013). SGLT2 inhibitor use remained high in both groups (87.5% vs 97.0%, p = 0.36).

Cardiac resynchronization therapy (CRT) was observed exclusively in the Levos group (50%, p = 0.001), suggesting a higher prevalence of electrical desynchrony. At baseline, prior to initiation of Levos therapy, levosimendan-exposed patients exhibited a higher burden of heart failure-related hospitalizations, consistent with their more advanced clinical status (37.5% vs 3.0%, p = 0.019). Follow-up clinical outcomes are reported separately in Table 2. During follow-up, cardiovascular hospitalization occurred in 3/8 (37.5%) levosimendan-exposed patients and 1/33 (3.0%) control patients, while cardiovascular death occurred in 1/8 (12.5%) and 0/33 (0.0%), respectively. Acute coronary syndrome occurred in 0/8 (0.0%) levosimendan-exposed patients and 1/33 (3.0%) controls, and no stroke events were recorded in either group. Composite cardiovascular events, defined as acute coronary syndrome, cardiovascular hospitalization, stroke, or cardiovascular death during follow-up, occurred in 3/8 (37.5%) levosimendan-exposed patients and 2/33 (6.1%) controls. Because exposed patients had more severe baseline disease and the analysis was retrospective, these outcomes are reported descriptively and should not be interpreted as causal evidence of treatment efficacy. Table 1.

**Table 1:** Baseline Characteristics of Patients with and without Long-Term Intermittent Levosimendan Therapy^a^

Sample size, n	Control (*n* = 33)	Levosimendan ≥ 22 sessions (*n* = 8)	p-value
Age, years	68.0 ± 10.0	75.8 ± 8.4	0.042
Sex, male, n/N (%)	23/32 (71.9)	7/8 (87.5)	0.653
Etiology, n/N (%)	Ischemic: 10/33 (30.3); Nonischemic: 23/33 (69.7)	Ischemic: 2/8 (25.0); Nonischemic: 6/8 (75.0)	1.000
LVEF at baseline, %	34.3 ± 10.2	24.5 ± 5.9	0.002
NYHA functional class at baseline, n/N (%)	I: 16/33 (48.5); II: 14/33 (42.4); III: 3/33 (9.1)	I: 1/8 (12.5); II: 1/8 (12.5); III: 6/8 (75.0)	<0.001
Minimum weight, kg	79.2 ± 15.1	69.0 ± 7.6	0.012
Weight, kg	79.7 ± 15.2	74.5 ± 11.5	0.300
Systolic BP, mmHg	115.9 ± 16.7	102.2 ± 16.2	0.058
Diastolic BP, mmHg	66.8 ± 12.0	59.5 ± 11.7	0.144
Heart rate, bpm	68.2 ± 12.5	66.0 ± 8.8	0.573
Congestion at baseline, n/N (%)	2/33 (6.1)	4/8 (50.0)	0.009
Therapeutic adjustment, n/N (%)	9/33 (27.3)	1/8 (12.5)	0.653
Hypertension, n/N (%)	25/33 (75.8)	3/8 (37.5)	0.084
Dyslipidemia, n/N (%)	26/33 (78.8)	3/8 (37.5)	0.034
Obesity, n/N (%)	12/33 (36.4)	1/8 (12.5)	0.398
Family history, n/N (%)	1/33 (3.0)	0/8 (0.0)	1.000
COPD, n/N (%)	4/33 (12.1)	1/8 (12.5)	1.000
Alcohol use, n/N (%)	10/33 (30.3)	2/8 (25.0)	1.000
Chronic heart failure, n/N (%)	11/33 (33.3)	2/8 (25.0)	1.000
Previous stroke, n/N (%)	2/32 (6.2)	1/8 (12.5)	0.498
Previous myocardial infarction, n/N (%)	8/33 (24.2)	1/8 (12.5)	0.659
Peripheral artery disease, n/N (%)	6/33 (18.2)	0/8 (0.0)	0.323
CRT, n/N (%)	0/33 (0.0)	4/8 (50.0)	<0.001
Diabetes status, n/N (%)	No: 23/33 (69.7); Type 1: 1/33 (3.0); Type 2: 9/33 (27.3)	No: 4/8 (50.0); Type 1: 0/8 (0.0); Type 2: 4/8 (50.0)	0.434
Smoking status, n/N (%)	Never: 17/33 (51.5); Former: 8/33 (24.2); Current: 7/33 (21.2); 3: 1/33 (3.0)	Never: 5/8 (62.5); Former: 3/8 (37.5); Current: 0/8 (0.0); 3: 0/8 (0.0)	0.474
Atrial fibrillation, n/N (%)	No: 23/33 (69.7); Paroxysmal: 7/33 (21.2); Persistent: 2/33 (6.1); Permanent: 1/33 (3.0)	No: 0/8 (0.0); Paroxysmal: 1/8 (12.5); Persistent: 0/8 (0.0); Permanent: 7/8 (87.5)	<0.001
CKD category, n/N (%)	0: 9/33 (27.3); 1: 11/33 (33.3); 2: 7/33 (21.2); 3: 4/33 (12.1); 4: 2/33 (6.1)	0: 0/8 (0.0); 1: 2/8 (25.0); 2: 0/8 (0.0); 3: 3/8 (37.5); 4: 3/8 (37.5)	0.020
Number of previous vascular events, n/N (%)	0: 22/33 (66.7); 1: 6/33 (18.2); 2: 3/33 (9.1); 3: 2/33 (6.1)	0: 7/8 (87.5); 1: 1/8 (12.5); 2: 0/8 (0.0); 3: 0/8 (0.0)	0.631
ACEI/ARB at baseline, n/N (%)	12/33 (36.4)	3/8 (37.5)	1.000
ARNI at baseline, n/N (%)	20/33 (60.6)	2/8 (25.0)	0.115
Ivabradine at baseline, n/N (%)	0/33 (0.0)	0/8 (0.0)	1.000
Beta-blocker at baseline, n/N (%)	30/33 (90.9)	7/8 (87.5)	1.000
MRA at baseline, n/N (%)	28/33 (84.8)	3/8 (37.5)	0.013
SGLT2 inhibitor at baseline, n/N (%)	32/33 (97.0)	7/8 (87.5)	0.356
GLP-1 agonist at baseline, n/N (%)	0/33 (0.0)	0/8 (0.0)	1.000
Furosemide at baseline, mg/day	42.4 ± 31.1	97.5 ± 39.2	0.005
Hemoglobin, g/dL	13.9 ± 2.0	12.2 ± 1.9	0.043
Platelets, 10^9/L	208.4 ± 60.6	157.0 ± 63.9	0.066
Creatinine, mg/dL	1.36 ± 0.46	2.13 ± 1.14	0.098
Urea, mg/dL	65.7 ± 25.3	118.3 ± 106.3	0.206
NT-proBNP, pg/mL	1782.9 ± 2445.9	6795.8 ± 5201.8	0.030
Ferritin	156.6 ± 105.8	—	—
Transferrin saturation, %	30.5 ± 11.3	49.0 ± 0.0	—
CRP	0.35 ± 0.52	0.67 ± 0.45	0.262

a *Data are presented as mean ± SD or n/N (%), as appropriate. p-values are descriptive because of the retrospective observational design and baseline imbalance between groups. BP, blood pressure; CKD, chronic kidney disease; COPD, chronic obstructive pulmonary disease; CRT, cardiac resynchronization therapy; LVEF, left ventricular ejection fraction; MRA, mineralocorticoid receptor antagonist; NT-proBNP, N-terminal pro-B-type natriuretic peptide; NYHA, New York Heart Association; SGLT2, sodium–glucose cotransporter 2.*

Follow-up clinical outcomes are summarized separately from baseline characteristics in Table 2. Cardiovascular hospitalization during follow-up occurred in 3/8 patients exposed to prolonged intermittent levosimendan and in 1/33 controls. Cardiovascular death occurred in 1/8 levosimendan-exposed patients and in 0/33 controls. Acute coronary syndrome occurred in 0/8 levosimendan-exposed patients and 1/33 controls, and no stroke events were recorded in either group. Composite cardiovascular events, defined as acute coronary syndrome, cardiovascular hospitalization, stroke, or cardiovascular death during follow-up, occurred in 3/8 levosimendan-exposed patients and 2/33 controls. Given the retrospective design, the small, exposed group, and marked baseline imbalance, these outcome data are reported descriptively and should not be interpreted as evidence of treatment efficacy.

**Table 2:** Follow-Up Treatment and Clinical Outcomes in Patients with and without Long-Term Intermittent Levosimendan Therapy^a^

Sample size, n	Control (*n* = 33)	Levosimendan ≥ 22 sessions (*n* = 8)	p-value
LVEF follow-up category (source coding 0–3), n/N (%)	0: 12/33 (36.4); 1: 11/33 (33.3); 2: 9/33 (27.3); 3: 1/33 (3.0)	0: 7/8 (87.5); 1: 1/8 (12.5); 2: 0/8 (0.0); 3: 0/8 (0.0)	0.072
ACEI/ARB at follow-up, n/N (%)	12/33 (36.4)	3/8 (37.5)	1.000
ARNI at follow-up, n/N (%)	20/33 (60.6)	2/8 (25.0)	0.115
Ivabradine at follow-up, n/N (%)	2/33 (6.1)	0/8 (0.0)	1.000
Beta-blocker at follow-up, n/N (%)	31/33 (93.9)	7/8 (87.5)	0.488
MRA at follow-up, n/N (%)	30/33 (90.9)	3/8 (37.5)	0.003
SGLT2 inhibitor at follow-up, n/N (%)	32/33 (97.0)	5/8 (62.5)	0.019
GLP-1 agonist at follow-up, n/N (%)	0/33 (0.0)	0/8 (0.0)	1.000
Furosemide at follow-up, mg/day	43.0 ± 32.1	97.5 ± 39.2	0.005
Acute coronary syndrome during follow-up, n/N (%)	1/33 (3.0)	0/8 (0.0)	1.000
Cardiovascular hospitalization during follow-up, n/N (%)	1/33 (3.0)	3/8 (37.5)	0.019
Noncardiovascular hospitalization during follow-up, n/N (%)	0/33 (0.0)	0/8 (0.0)	1.000
Hemorrhagic complication during follow-up, n/N (%)	0/33 (0.0)	0/8 (0.0)	1.000
Stroke during follow-up, n/N (%)	0/33 (0.0)	0/8 (0.0)	1.000
Cardiovascular death during follow-up, n/N (%)	0/33 (0.0)	1/8 (12.5)	0.195
Noncardiovascular death during follow-up, n/N (%)	0/33 (0.0)	0/8 (0.0)	1.000
Death of unknown cause during follow-up, n/N (%)	0/33 (0.0)	0/8 (0.0)	1.000
Composite cardiovascular events during follow-up, n/N (%)	2/33 (6.1)	3/8 (37.5)	0.043

a *Data are presented as mean ± SD or n/N (%), as appropriate. Follow-up comparisons are descriptive and should not be interpreted as causal treatment effects. The current source file contains LVEF follow-up as a coded 0–3 variable rather than a continuous percentage; replace this row with decoded LVEF values if available. Follow-up NYHA class and follow-up NT-proBNP values were not available as separate variables in the uploaded source file.*

### Proteomic Data

Proteomic analysis was performed on plasma samples collected after all levosimendan-exposed patients had reached the predefined threshold of at least 22 intermittent infusion cycles. The resulting profile should therefore be interpreted as a cross-sectional molecular phenotype after prolonged exposure, rather than as an acute postinfusion response or a longitudinal within-patient treatment effect. Because retrospective records did not consistently capture the exact cumulative number of infusions, total treatment duration, or standardized interval from the last infusion to blood collection, the levosimendan group represents patients exposed above a defined minimum threshold rather than a cohort with homogeneous cumulative dosing. These temporal and dosing limitations, together with the small exposed-group size, precluded formal time-to-event or survival analyses; clinical outcomes, including mortality, are therefore presented descriptively only. Proteomic profiling was performed on plasma samples collected remote from the last Levos infusion, thereby reflecting a stable treatment-associated molecular profile rather than acute pharmacological effects. Thus, proteomic analysis of plasma samples from the Cont and Levos-exposed groups identified a total of 1,824 proteins, each validated by at least two distinct peptides. Of these, 706 proteins were present in both conditions, constituting the core plasma proteome, while 69 proteins were unique to the Cont group and 35 were unique to the Levos group. The Cont group contained 775 proteins, and the Levos group contained 741 proteins. Thus, 83.6% of the proteins were shared between the two groups. ShyGO’s gene ontology (GO) enrichment analysis showed that each group had a distinct biological process signature. In the Cont group (Figure 1A), enriched terms were mainly related to actin cytoskeleton movement and cell adhesion, including lamellipodium assembly and organization, actin filament and cytoskeleton organization, and cell–substrate adhesion. Other overrepresented categories included the catecholamine metabolic process and immune system processes. This indicates that structural integrity and inflammatory signaling are actively regulated even at rest. In contrast, the levosimendan-exposed group (Figure 1B) showed enrichment in metabolic and stress-adaptive pathways, such as the oxaloacetate metabolic process, chaperone-mediated autophagy, pyridine nucleotide metabolism, d-glucose import, and response to heat and temperature stimuli. These findings suggest a shift toward enhanced cellular metabolism, redox balance, and proteostatic processes following Levos exposure. These enrichment profiles indicate that Levos treatment is associated with functional reprogramming of the plasma proteome, shifting it from cytoskeletal and immune-related processes to metabolic adaptation and stress resilience, consistent with its known cardioprotective and mitochondrial-modulating effects.

**Figure 1:**
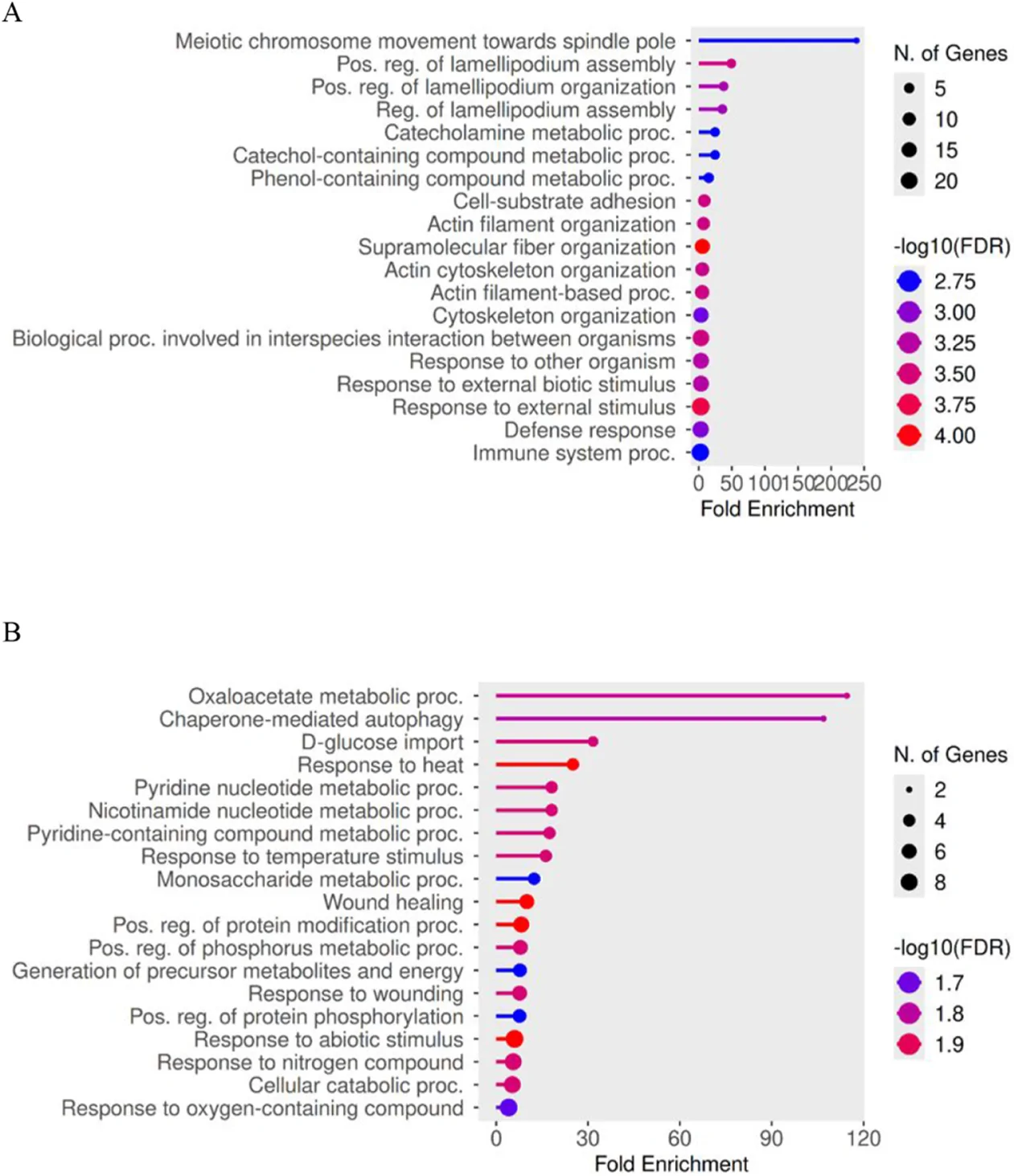
ShyGO enrichment analysis of biological processes in Cont (A) and Levos (B) groups.

After applying strict quality filters (missingness < 20%, CV < 30%), 538 proteins were retained for quantitative analysis of all samples. Partial Least Squares–Discriminant Analysis (PLS-DA) of these proteins demonstrated a clear global separation between the Cont and Levos groups. Figure 2 shows that Component 1 (8.6%) and Component 2 (17.3%) together accounted for approximately 26% of the variance. The samples were tightly clustered within their 95% confidence ellipses, indicating high similarity within groups and suggested a tendency toward group-wise separation at the global proteomic level, which should be interpreted cautiously given the exploratory nature of the analysis. Hierarchical clustering of normalized protein abundances further confirmed the presence of two distinct expression modules associated with Cont and levosimendan-exposed patients. ShyGO’s functional annotation of these clusters revealed substantial differences in the biological processes active in the two proteomic profiles. Proteins more abundant in the Cont samples were primarily associated with immune and inflammatory processes, including innate and mucosal immune responses, complement activation via the classical pathway, humoral immune response mediated by circulating immunoglobulins, and defense responses to bacterial and external biotic stimuli. C-reactive protein (CRP), serum amyloid A1 and A2 (SAA1/2), complement components C2, C4B, C4BPB, and C7, inter-α-trypsin inhibitor heavy chain 4 (ITIH4), and several immunoglobulin variable and constant domains (IGHV, IGKV, IGLV) were the most prominent proteins in this group. These features indicate that the Cont plasma proteome reflects an activated immune environment with complement cascade engagement and acute-phase responses. In contrast, proteins elevated in the levosimendan-exposed group indicated a shift toward anti-inflammatory and cytoprotective mechanisms. The most overrepresented biological processes included the acute-phase response, negative regulation of complement and humoral immune activation, opsonization, blood coagulation, and reactive oxygen species (ROS) metabolic processes. Wound healing, regulation of body fluid balance, and hemostasis were also among the most enriched categories. Key proteins contributing to these processes were selenoprotein P (SELENOP), glutathione peroxidase 3 (GPX3), vitamin K–dependent protein S (PROS1), prothrombin (F2), alpha-2-HS-glycoprotein (AHSG), and aldolase A (ALDOA). To provide orthogonal validation of the proteomic findings, slot blot analysis was performed for aldolase A (ALDOA). Slot blot analysis of ALDOA showed a trend consistent with the DIA proteomic data; however, due to the semiquantitative nature of the assay and signal variability, this validation should be interpreted as supportive rather than confirmatory. The representative membrane and quantification are presented in Supplementary Figure S1. DIA-based quantification (Figure 3), performed with FDR < 1% at precursor and protein levels, provides the primary quantitative evidence in this study. In contrast, antibody-based validation in plasma is inherently limited by dynamic range constraints and epitope accessibility. Orthogonal validation was attempted but yielded variable signal quality; therefore, conclusions rely primarily on DIA-based quantification. Their coordinated upregulation suggests adaptive proteomic remodeling characterized by enhanced antioxidant capacity, optimized redox homeostasis, and regulated modulation of coagulation and inflammatory responses. These findings demonstrate that levosimendan treatment shifts the circulating proteome from immune activation toward metabolic optimization and oxidative stress resistance. This pattern supports the PLS-DA clustering results, which separated the two conditions, and aligns with current knowledge of levosimendan’s cardioprotective effects, including improved mitochondrial efficiency, anti-inflammatory signaling, and endothelial stabilization. These multivariate patterns should be interpreted cautiously, as missing value imputation in small cohorts may influence apparent group separation. Proteomic profiling was performed at a single time point after prolonged therapy, without baseline molecular measurements. Therefore, the observed differences represent cross-sectional proteomic signatures associated with long-term levosimendan exposure rather than within-patient treatment-induced changes. Figure 3.

**Figure 2:**
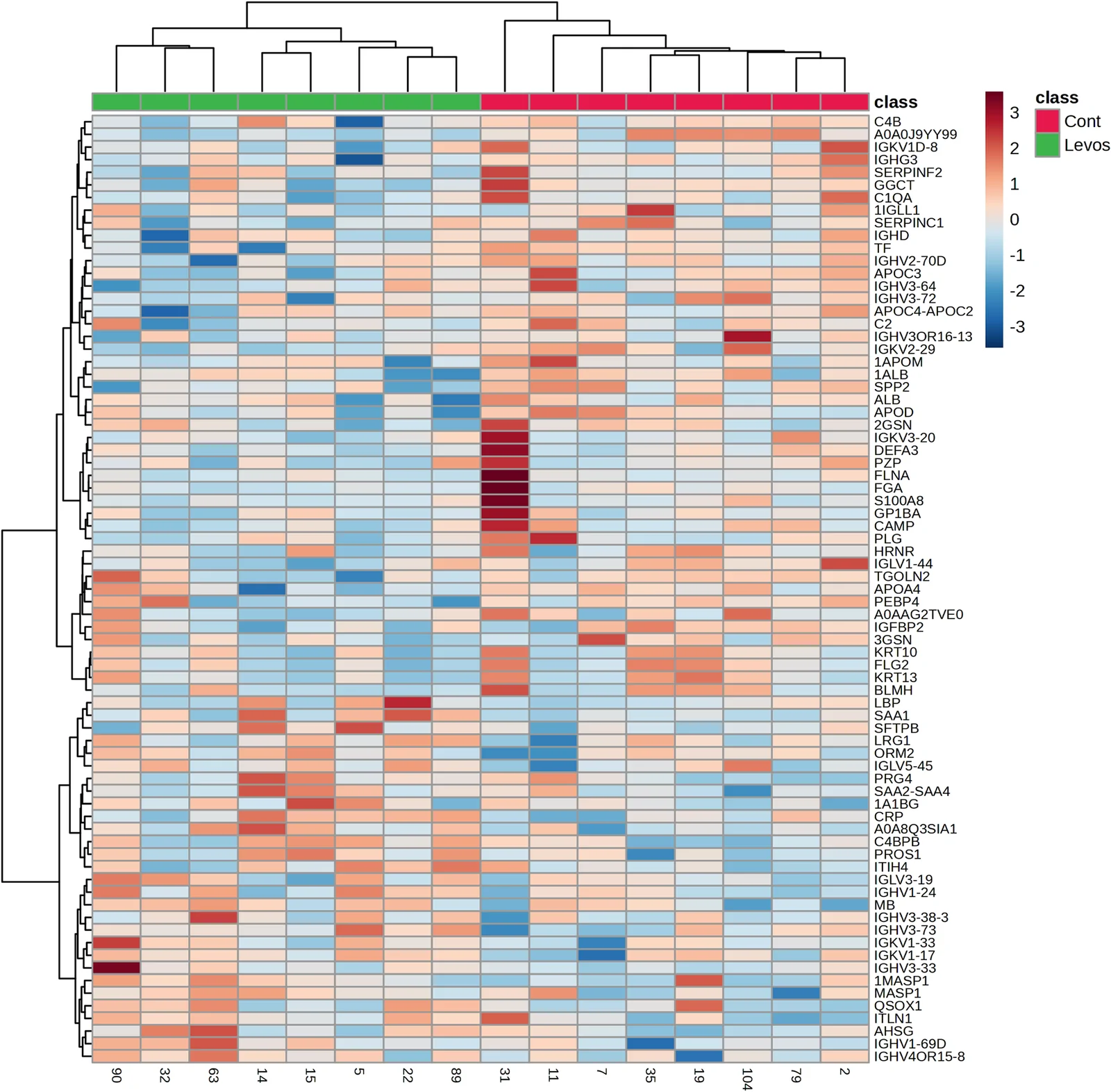
Heatmap of hierarchical clustering of DIA-quantified plasma proteins (top *n* = 100) showing differences between Cont and Levos-exposed patients. Protein intensities were log2-transformed, Z-score normalized across samples, and subjected to unsupervised hierarchical clustering using Euclidean distance and Ward’s linkage. The heatmap reveals two main sample clusters (top dendrogram), corresponding to the Cont group (red) and the Levos-exposed group (green). Rows represent different proteins, and columns represent patient samples. Blue indicates lower abundance, and red indicates higher abundance. The protein-level dendrogram (left) highlights distinct coexpression modules, reflecting coordinated biological responses. This clustering pattern demonstrates clear separation between the groups and identifies the proteins most responsible for the molecular differences associated with treatment. PLS-DA is shown for exploration visualization only; no predictive modeling or statistical inference was derived from this analysis due to the limited sample size.

**Figure 3:**
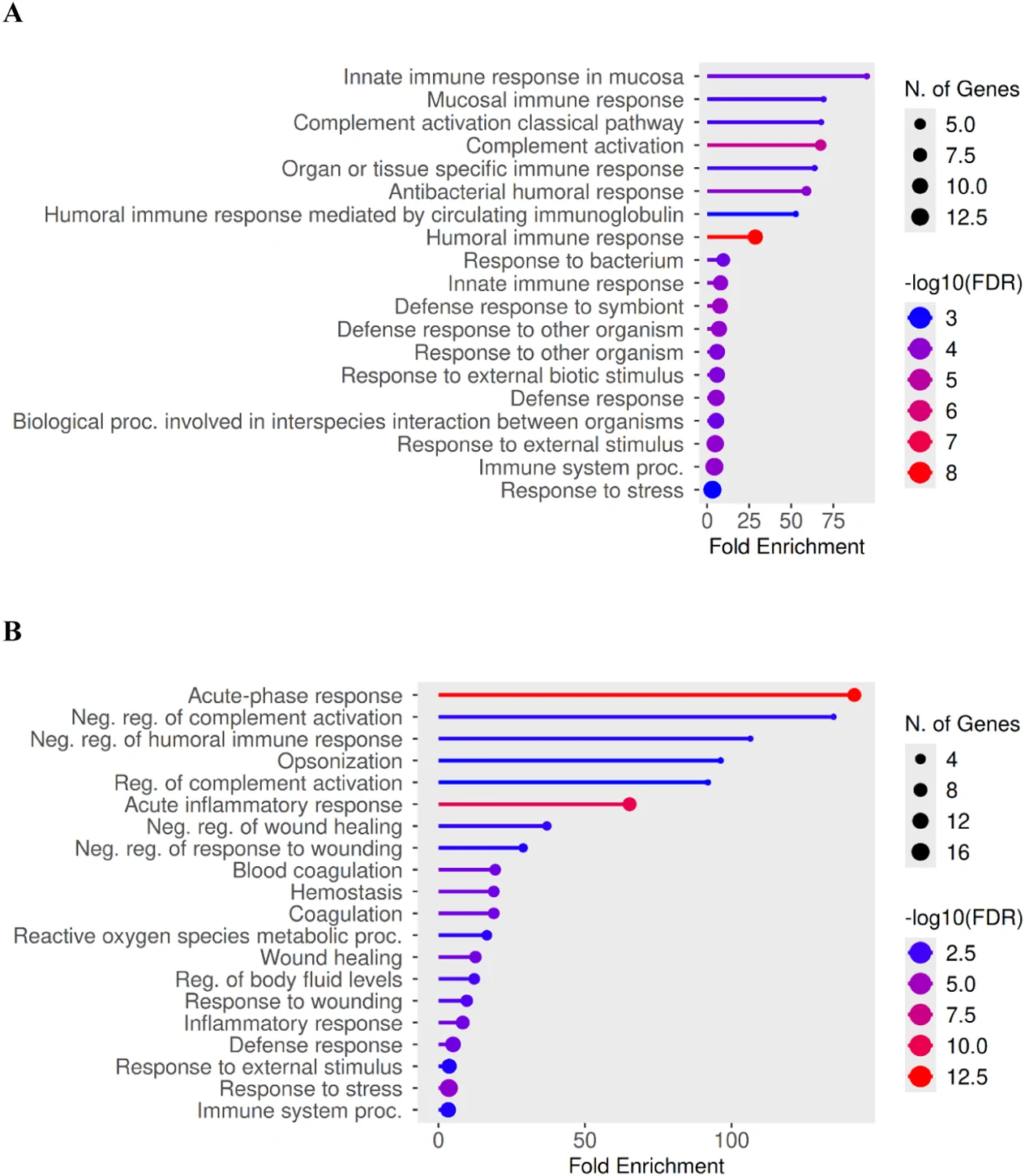
Functional enrichment analysis of differentially abundant proteins using ShyGO. (A) Cont group: enriched biological processes were dominated by immune and inflammatory pathways, including mucosal and innate immune responses, complement activation (classical pathway), antibacterial humoral responses, and immunoglobulin-mediated humoral immunity. (B) Levos group: enrichment shifted toward processes associated with acute-phase regulation, negative regulation of complement activity, oxidative stress metabolism, coagulation/hemostasis, wound healing, and tissue repair, indicating a more cytoprotective and adaptive response profile. Dot size reflects the number of proteins contributing to each GO term, while color intensity represents the statistical significance of enrichment (−log10 FDR). Fold enrichment is plotted on the *x*-axis, highlighting the magnitude of pathway overrepresentation in each group.

## Discussion

In this cohort, patients receiving more than 22 intermittent levosimendan infusions exhibited clinical stability over follow-up, despite more severe baseline disease. Because treatment allocation was not randomized and baseline disease severity differed substantially between groups, observed clinical and molecular differences may reflect underlying patient characteristics rather than direct pharmacological effects. Importantly, because plasma proteomics were obtained at a single follow-up time point without baseline sampling, the present study does not permit inference of causal treatment effects or temporal molecular remodeling. The identified proteomic patterns therefore represent cross-sectional phenotypes associated with prolonged levosimendan exposure in a real-world advanced heart failure population. These results are consistent with previous studies on intermittent Levos in advanced heart failure. In LION-HEART, biweekly infusions over 12 weeks significantly reduced NT-proBNP, improved quality of life, and lowered the risk of heart failure rehospitalization by approximately 75%. Caution is needed to analysis of the results of NT-proBNP because the levosimendan arm has worse creatinine and subsequently higher NT-proBNP values. The presence in the arm of levosimendan, of a worst ejection fraction and NYHA class, reinforces the results and the interpretation of NT-proBNP, even knowing we cannot compare the absolutes values. A 2024 meta-analysis of 15 trials (1,181 patients) further confirmed that intermittent Levos increases LVEF by about 6 percentage points, reduces natriuretic peptides, and decreases all-cause mortality (risk ratio ≈0.60).^6^ Although earlier studies suggested neutral effects on overall mortality, they consistently reported reductions in cardiovascular death.^11^ Because no deaths occurred in the levosimendan-exposed group during follow-up, mortality comparisons between groups were only descriptive and not suitable for survival analysis. So, patients exposed to Levos had a much poorer clinical state at the start of the study. Therefore, the clinical trajectories shown reflect stabilization or improvement but cannot be concluded that Levos is superior to other treatments. Our study builds on these findings by examining significantly longer treatment duration (>20 sessions), a period rarely investigated in previous research. The Cont group was intentionally selected from the same real-world advanced heart failure population rather than from a matched or trial-defined cohort. This approach increases clinical relevance but also introduces variability in prior decompensation histories and treatment pathways. These differences are a normal part of decision-making in advanced heart failure and must be considered when comparing groups, especially when analyzing proteomic data. Although levosimendan-exposed patients had a higher baseline hospitalization burden reflecting more advanced disease, follow-up after treatment initiation was associated with clinical stabilization despite worse initial status. However, this observation should not be interpreted as evidence of a causal treatment effect. It is important to note that the proteome variations identified in this study cannot be considered definitive treatment-induced changes. Proteomic profiling was performed at a single time point after extended therapy, preventing within-patient longitudinal comparisons. Therefore, the observed molecular signatures should be viewed as a cross-sectional phenotype associated with prolonged Levos exposure, rather than as direct indicators of temporal proteome remodeling. Because plasma samples were obtained outside the acute postinfusion period, the observed proteomic signatures are more consistent with sustained proteomic phenotypes associated with prolonged Levos exposure rather than transient drug-induced changes. A novel aspect of our study is the integration of plasma proteomics, providing, to the best of our knowledge, an initial characterization of cross-sectional systemic molecular profiles associated with long-term levosimendan exposure in humans. Importantly, the molecular conclusions are primarily supported by DIA-based quantification with stringent FDR control (<1% at precursor and protein levels). Antibody-based validation approaches in plasma remain technically constrained, and in our study yielded variable signal quality; therefore, they are interpreted as supportive rather than confirmatory. After more than 22 infusion cycles, prolonged levosimendan exposure was associated with higher representation of antioxidant, metabolic, and cellular stress-response pathways, together with lower relative representation of immune and inflammatory processes. These cross-sectional molecular patterns are compatible with biological effects previously reported in short-term studies. Acute Levos administration has been shown to reduce inflammatory cytokines (IL-6, TNF-α, IL-1β, MCP-1) within hours to 12 days and to decrease oxidative stress markers such as nitrosylated proteins and lipid peroxidation products.^12^ However, prior studies were limited to acute or subacute settings. Our findings provide preliminary evidence that prolonged levosimendan exposure is associated with a plasma proteomic phenotype characterized by antioxidant defense and lower relative inflammatory pathway representation.

The biological plausibility of these proteomic associations is supported by preclinical research. Levos enhances myocardial contractility via calcium sensitization without raising intracellular calcium, limiting metabolic demand. It also activates vascular and mitochondrial K_ATP channels, improves oxygen utilization, and exerts antiapoptotic and anti-inflammatory effects in experimental models.^13^ Activation of Nrf2-mediated antioxidant pathways, mitochondrial protection, and modulation of inflammatory signaling have been documented in cardiomyocytes and septic cardiomyopathy models.^13^ Our proteomic data are compatible with these mechanisms, showing higher abundance of antioxidant proteins, including GPX3 and SELENOP, and enrichment of metabolic-adaptive pathways. However, these findings should be interpreted as associative and do not demonstrate durable biological remodeling induced by therapy. These findings are consistent with previously described cellular effects of levosimendan and may represent biological correlates of clinical stability during long-term use.

A further strength of our study is the focus on elderly, highly comorbid, real-world advanced heart failure population, distinct from traditional trial cohorts. Many of our patients had significant cardiorenal syndrome, chronic kidney disease near guideline contraindication thresholds, diabetes, anemia, or other comorbidities that often lead to exclusion from clinical trials. This explains the markedly lower use of MRAs in our cohort, largely due to hyperkalemia and renal dysfunction, in contrast to guideline-directed therapy uptake in randomized studies. Our findings therefore apply to a “no-option” phenotype of advanced heart failure frequently encountered in clinical practice but rarely represented in trials.

Another notable feature of our cohort is the high prevalence of CRT devices, indicating substantial electrical desynchrony. While CRT improves outcomes in advanced heart failure, previous Levos studies did not stratify or analyze responses according to device therapy status. Our results suggest that prolonged levosimendan use in patients with CRT was associated with clinical stability; however, the study design does not allow conclusions regarding additive benefit or interaction with device therapy. The safe use of Levos in individuals with significant renal impairment, anemia, diabetes, and multiple comorbidities reinforces its feasibility in complex advanced heart failure patients.^1^

Overall, our findings extend existing observational data by describing the clinical trajectories and cross-sectional molecular profiles of patients receiving prolonged intermittent levosimendan in routine care. While the data are consistent with clinical stabilization and distinct proteomic patterns, the study design does not permit causal inference regarding treatment efficacy or molecular remodeling. This study fills an important gap by describing cross-sectional plasma proteomic profiles associated with prolonged levosimendan exposure in elderly, frail, multimorbid patients who are often ineligible for optimized neurohormonal therapy. These findings support further prospective studies to determine whether levosimendan contributes directly to clinical stabilization or molecular adaptation in this population.

### Study Limitations

This study has several limitations that should be considered when interpreting the findings. The retrospective design introduces potential confounding, and the two groups differed in baseline characteristics, reflecting real-world treatment decisions. Although such differences are expected in advanced HF populations, they may influence clinical and proteomic comparisons. The number of patients receiving long-term Levos was modest, limiting statistical power to detect differences in outcomes such as mortality. Since individuals exposed to Levos were selected due to clinical deterioration and survival during extended therapy, survivor bias is possible. Additionally, the absence of baseline proteome samples limits causal inference, and the observed changes may reflect preexisting biological variations or adaptive responses unrelated to therapy. Detailed quantification of prior heart failure decompensation episodes and cumulative hospitalization burden was not uniformly available for all patients, limiting more granular stratification of disease trajectory between exposed and unexposed groups. Precise cumulative levosimendan exposure could not be reconstructed for all patients, introducing heterogeneity in treatment intensity and duration within the levosimendan group. This precludes dose-response analysis and limits biological interpretation at the level of quantified exposure. Accordingly, proteomic differences should be interpreted as cross-sectional phenotypes associated with prolonged intermittent levosimendan therapy rather than exposure-standardized molecular effects. Plasma sampling was not performed at a standardized time point relative to levosimendan administration and typically occurred approximately 15 days after the last infusion. Given the long-acting active metabolite of levosimendan, interindividual variability in residual pharmacodynamic effects is likely. This heterogeneity further limits mechanistic interpretation of proteomic differences and reinforces that the observed molecular patterns represent cross-sectional phenotypes associated with prolonged therapy rather than synchronized pharmacodynamic responses. Baseline clinical characteristics and proteomic sampling were not temporally aligned. Clinical variables were extracted at treatment initiation, whereas plasma samples were collected later during follow-up. In addition, the control group was not matched to the exposed patients by disease stage at the time of sampling or by baseline characteristics with proteomic profiling after an equivalent follow-up period. These design constraints introduce structural confounding and limit causal interpretation. Consequently, the proteomic comparisons reflect cross-sectional molecular phenotypes of two clinically distinct advanced heart failure populations rather than treatment-associated effects.

Proteomic analyses were performed in a subset of patients and represent a single sampling point after extended therapy; therefore, the temporal evolution of molecular changes could not be assessed. In addition, while DIA mass spectrometry offers comprehensive coverage, it may not fully capture very low-abundance proteins or subtle post-translational modifications. Using KNN-based imputation on a small proteomic data set presents another issue. Even when applied after rigorous missingness filtering, imputation may partially increase group differences or reduce variation. Therefore, these results should be considered hypothesis-generating rather than confirmatory. Using supervised multivariate methods such as PLS-DA with small-sample, high-dimensional proteomic data sets presents a significant risk of overfitting. Therefore, this study restricted PLS-DA to exploratory visualization and did not use it for hypothesis testing or classification. Biological interpretation primarily relied on unsupervised clustering and pathway-level enrichment analyses, which are more robust to limitations in data size. Most critically, the absence of baseline proteomic measurements precludes within-patient comparison and prevents attribution of molecular differences directly to levosimendan treatment. Consequently, the proteomic findings should be interpreted strictly as associative and hypothesis-generating. The ALDOA validation was performed using slot blot rather than full Western blot, which limits molecular weight resolution and may reduce specificity. Signal variability and the lack of band-based validation reduce confidence in quantitative interpretation. A prospective longitudinal design with defined exposure and repeated sampling would be required to establish treatment-related molecular effects. Finally, our cohort consisted primarily of older, multimorbid patients, many with significant cardiorenal impairment, which may limit the generalizability of the results to broader HF populations.

## Conclusion

In this real-world retrospective cohort of advanced heart failure patients, prolonged intermittent levosimendan exposure identified a clinically more severe subgroup with distinct cross-sectional plasma proteomic features. Compared with nonexposed controls, levosimendan-exposed patients showed higher representation of proteins involved in antioxidant defense, redox regulation, coagulation/hemostasis, and metabolic adaptation, whereas controls showed higher representation of immune and inflammatory pathways. Because treatment allocation was nonrandomized, baseline severity differed substantially between groups, and plasma proteomics were performed only at follow-up, these findings should be interpreted as exploratory and hypothesis-generating. Prospective controlled studies with standardized exposure assessment and serial proteomic sampling are required to determine whether these molecular profiles reflect treatment-associated adaptation, disease severity, or both.

## Supplementary Material



## Data Availability

Mass spectrometry proteomics data were deposited at the ProteomeXchange Consortium via the PRIDE repository with data set identifier PRIDE with reference: 1–20251108–121316–5079579.

## Notes

Figure S1: representative ALDOA slot blot membrane and densitometric quantification supporting the DIA-based plasma proteomic analysis; Table S1: protein groups identified and quantified by DIA mass spectrometry across the plasma samples included in the study (PDF)
