# Supplementary material for: Cross-Sectional Plasma Proteomic Phenotypes Associated with Prolonged Intermittent Levosimendan Exposure in Advanced Heart Failure
Source: ACS Pharmacol Transl Sci. 2026 Aug 25;9(9):2513–24. doi: 10.1021/acsptsci.6c00195 (PMC13632881; doi:10.1021/acsptsci.6c00195)
Supplement: Supplementary file 1 [file pt6c00195_si_001.pdf]

Supporting Information

Cross-Sectional Plasma Proteomic Phenotypes Associated with Prolonged Intermittent Levosimendan Exposure in Advanced Heart Failure

José Mesquita Bastos[1,2], Ines Cruz[1], Luis Santos[1], and Rui Vitorino[2,3]*

[1] Serviço de Cardiologia da Unidade de Saúde Local Região de Aveiro, Aveiro, Portugal

[2] Institute of Biomedicine (iBiMED), Department of Medical Sciences, University of Aveiro, Aveiro, Portugal

[3] Cardiovascular R&D Centre – UnIC@RISE, Department of Surgery and Physiology, Faculty of Medicine, University of Porto, Porto, Portugal

*Corresponding author: Rui Vitorino

Email: rvitorino@ua.pt

Contents

Figure S1. Representative ALDOA slot blot membrane and densitometric quantification supporting the DIA-based plasma proteomic analysis.

Table S1. Protein groups identified and quantified by DIA mass spectrometry across the plasma samples included in the study.

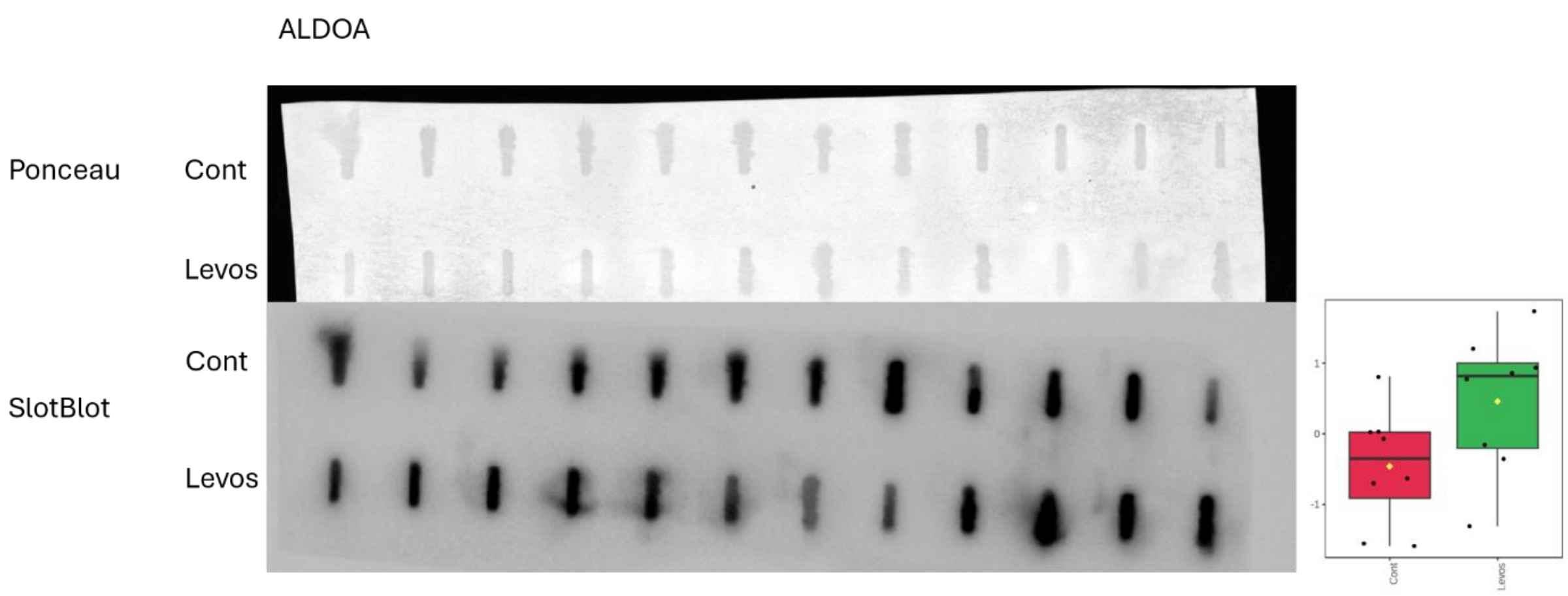

Figure S1. Slot blot validation of ALDOA expression in plasma samples from control and levosimendan-treated patients. Equal amounts of plasma protein were applied to membranes and probed using anti-ALDOA antibody (AB150396). Ponceau S staining was used as a loading control.

Protein Group	Protein Names	Genes	First.Protein.Description	35	11	31	79	19	7	104	2	5	14	15	22	32	63	89	90
P04217	A1BG_HUMAN	A1BG	Alpha-1B-glycoprotein	2E+09	2,19E+09	2,06E+09	1,98E+09	1,88E+09	2,09E+09	1,69E+09	2,05E+09	2,05E+09	1,62E+09	2,24E+09	2,03E+09	1,39E+09	2,2E+09	1,67E+09	2,12E+09
P04217-2	A1BG_HUMAN	A1BG	Isoform 2 of Alpha-1B-glycoprotein	25330200	25538600		25721100	21945400	27077700	20445300	18064800	36274700	23706600	42743300	26947500	24051800	30633200	18784400	28950900
P01023	A2MG_HUMAN	A2M	Alpha-2-macroglobulin	8,21E+09	8,12E+09	6,95E+09	9,83E+09	8,69E+09	8,85E+09	6,04E+09	5,14E+09	5,14E+09	4,07E+09	5,99E+09	6,52E+09	9,72E+09	1,36E+10	5,73E+09	1,13E+10
P60709	ACTB_HUMAN	ACTB	Actin, cytoplasmic 1	59857200	77554400	1,11E+10	39625900	83511700	49597200	2,19E+08	44283800	67295600	86111100	94043200	79980200	42818800	2,67E+08	56556800	53129400
Q562R1	ACTBL_HUMAN	ACTBL2	Beta-actin-like protein 2	22868300	27564800	3,3E+09	19438700	30585000	19286900	1,04E+08	14140700	26059300	30948900	37217300	35720400	16496900	1,03E+08	19071400	22251100
O15204	ADEC1_HUMAN	ADAMDEC1	ADAM DEC1	695971		1043860	606687	964287	1447730		921800	702987	363129	567635	425793	926372	445616	470724	1013100
Q76LX8	ATS13_HUMAN	ADAMTS13	A disintegrin and metalloproteinas	1476930	1536340		924929	1429820	1345150	1023600	1356650	1553500	2343730	1382390	1543420	1356470	2180510	1406930	1274670
Q86TH1	ATL2_HUMAN	ADAMTSL2	ADAMTS-like protein 2	1149060	625422		786076	1791210	900260	623480	756449	588448	852825	517361	940172	767026	1145060	1359900	914127
Q6UY14	ATL4_HUMAN	ADAMTSL4	ADAMTS-like protein 4	1951630	1980600	2701690	2878780	3845120	3199510	2272560	1644830	1206970	1073660	2148640	2169330	2505330	2763060	2447880	3485210
P48960	AGRE5_HUMAN	ADGRE5	Adhesion G protein-coupled recept	74558	71527.6	242359	71968.3		141657	151990	170322	66284.9			105458	148014	163596	75743.4	162495
Q86SQ4	AGRG6_HUMAN	ADGRG6	Adhesion G-protein coupled recept	572507	645897			780432	654529	401574	384946	641793	597243	656059	877289		625526	454679	557159
Q15848	ADIPO_HUMAN	ADIPOQ	Adiponectin	91180700	39572300	47907000	38370000	46189900	64679900	56089200	89110800	41433600	18079500	27587900	37496600	79278400	1,13E+08	66384300	98895500
P43652	AFAM_HUMAN	AFM	Afamin	2,03E+09	3,97E+09	2,24E+09	2,3E+09	1,9E+09	2,37E+09	2,1E+09	3,41E+09	2,78E+09	5,05E+09	2,57E+09	2,77E+09	2,88E+09	3,58E+09	1,85E+09	1,84E+09
P01019	ANGT_HUMAN	AGT	Angiotensinogen	5,12E+08	7,88E+08	7,37E+08	6,35E+08	4,24E+08	9,2E+08	5,14E+08	8,12E+08	4,99E+08	6,75E+08	5,9E+08	6,53E+08	5,46E+08	4,03E+08	7,08E+08	5,68E+08
P02765	FETUA_HUMAN	AHSG	Alpha-2-HS-glycoprotein	2,29E+09	3,26E+09	3,44E+09	2,71E+09	2,2E+09	2,91E+09	2,57E+09	3,23E+09	2,34E+09	2,63E+09	2,99E+09	3,57E+09	4,48E+09	5,07E+09	3,69E+09	3,03E+09
A0A0C4DGB6	A0A0C4DGB6_HUMAN	ALB	Albumin	5,78E+10	7,05E+10	8,13E+10	6,71E+10	7,57E+10	6,17E+10	5,83E+10	6,68E+10	4,56E+10	6,77E+10	7,21E+10	6,47E+10	6,31E+10	6,22E+10	4,2E+10	6,76E+10
B7WNR0	B7WNR0_HUMAN	ALB	Albumin	4,21E+10	4,89E+10	4,49E+10	3,35E+10	4,28E+10	4,49E+10	4,92E+10	4,26E+10	3,72E+10	4,4E+10	4,36E+10	3,1E+10	4,09E+10	4,45E+10	3,03E+10	4,15E+10
P04075	ALDOA_HUMAN	ALDOA	Fructose-bisphosphate aldolase A	5009790		2,78E+08		4385160	3936810	8070670	1867740	11990800	8383580	4493350	5945270	58074000	25731200	4052370	
P05062	ALDOB_HUMAN	ALDOB	Fructose-bisphosphate aldolase B	2712450	35013900	19911500	1497420	7141830	6089020	3418880	1126080	7268140	7861780	5216000	37240700	8845800	5238930	2736530	5944360
P02760	AMBP_HUMAN	AMBP	Protein AMBP	9,82E+08	9,49E+08	9,78E+08	1,21E+09	1,17E+09	8,73E+08	1,07E+09	9,79E+08	8,47E+08	1E+09	8,87E+08	1,1E+09	1,17E+09	8,86E+08	9,74E+08	1,58E+09
P03950	ANGI_HUMAN	ANG	Angiogenin	3529270	2286280	3911680	5132720	2931620	2766470	2146760	4706420	2440970	3093430	4099840	2679220	2527500		3194370	1425560
Q9Y5C1	ANGL3_HUMAN	ANGPTL3	Angiopoietin-related protein 3	2102070	469853	945880	1081050	1610620		1197180		891911	1039000		1508130	2205780	1149740	1736730	1891330
P15144	AMPN_HUMAN	ANPEP	Aminopeptidase N	3007950	2940350	2962410	1509270	4589990	2812500	1525860	1451980	2393260	1769800	2493210	2880580	2006170	2755660	3818610	3460530
P58335	ANTR2_HUMAN	ANTXR2	Anthrax toxin receptor 2	1347970	4234300	3176500	938335	1223420	2362650	2122230	1158560	1035540	1703860	2364650	1842440	2230300	1741630	1007080	1486360
P07355	ANXA2_HUMAN	ANXA2	Annexin A2	945961		4205740	418484	2468110	580240	898502		1542450	680719	437139	622809	813076	12063200	734646	959956
Q16853	AOC3_HUMAN	AOC3	Amine oxidase [copper-containing]	2136410	989355		969355	4496460	3035410		2832970	1133610	1754520	1069270	978575	1426110	3441410	2491160	1716400
P02743	SAMP_HUMAN	APCS	Serum amyloid P-component	2,23E+08	4,69E+08	5,56E+08	2,19E+08	2,76E+08	3,05E+08	4,4E+08	2,72E+08	3,42E+08	5,54E+08	6,81E+08	3,5E+08	3,73E+08	3,03E+08	3,29E+08	3,39E+08
Q9HDC9	APMAP_HUMAN	APMAP	Adipocyte plasma membrane-assc	4387770	18564900	34575300	12073700	6462220	8720420	18495100	8180230	9337020	3914160	19744100	7114960	13980200	11422800	5483910	8826030
P02647	APOA1_HUMAN	APOA1	Apolipoprotein A-I	2,24E+10	3,47E+10	2,66E+10	1,62E+10	1,29E+10	2,05E+10	2,04E+10	2,27E+10	1,8E+10	2,08E+10	1,9E+10	1,84E+10	2,33E+10	2,23E+10	2,03E+10	1,98E+10
P02652	APOA2_HUMAN	APOA2	Apolipoprotein A-II	6,18E+09	1,37E+10	1,17E+10	5,94E+09	4,49E+09	7,23E+09	7,33E+09	6,22E+09	7,11E+09	6,98E+09	9,38E+09	6,41E+09	7,26E+09	8,02E+09	3,87E+09	7,77E+09
P06727	APOA4_HUMAN	APOA4	Apolipoprotein A-IV	1,41E+09	1,29E+09	1,61E+09	9,3E+08	1,15E+09	1,87E+09	1,94E+09	1,25E+09	5,98E+08	3,42E+08	1,02E+09	1,02E+09	1,77E+09	1,13E+09	9,03E+08	2,29E+09
Q6Q788	APOA5_HUMAN	APOA5	Apolipoprotein A-V		108795	65918.3	62844.6	89069.8	46200.9		92681	55965.7	64768.8	50207.5	46588.7		35605.4	44675.8	
A0AAG2UUW0	A0AAG2UUW0_HUMAN	APOB	Apolipoprotein B	8,38E+08	1,9E+09	2,33E+09	1,11E+09	1,45E+09	1,59E+09	1,73E+09	1,46E+09	1,6E+09	2,88E+09	1,6E+09	1,62E+09	1,41E+09	2,09E+09	9,5E+08	1,58E+09
P02654	APOC1_HUMAN	APOC1	Apolipoprotein C-I	3,5E+08	1,06E+09	4,55E+08	3,75E+08	2,74E+08	4,53E+08	4,41E+08	4,56E+08	4,08E+08	5,41E+08	5,7E+08	3,26E+08	2,62E+08	4,56E+08	5,05E+08	4,97E+08
P02656	APOC3_HUMAN	APOC3	Apolipoprotein C-III	1,53E+09	4,39E+09	2,02E+09	2,41E+09	2,27E+09	1,58E+09	2,27E+09	2,85E+09	1,47E+09	1,93E+09	9,64E+08	2,4E+09	1,18E+09	1,14E+09	1,82E+09	2,12E+09
P55056	APOC4_HUMAN	APOC4	Apolipoprotein C-IV	5071710	17962900	20115500	12087400	3930590	10686300	11024600	29158000	12209300	30065600	9513200	10820400	1998810	2525260	16161100	9700960
K7ER74	K7ER74_HUMAN	APOC4-APOC2	Apolipoprotein C-II	4,65E+08	8,31E+08	7,59E+08	6,14E+08	4,69E+08	6,1E+08	6,11E+08	9,67E+08	5,08E+08	7,13E+08	7,19E+08	7,6E+08	1,69E+08	3,02E+08	5,61E+08	4,61E+08
P05090	APOD_HUMAN	APOD	Apolipoprotein D	1,13E+09	1,64E+09	1,16E+09	7,8E+08	1,03E+09	1,53E+09	9,27E+08	7,58E+08	5,19E+08	8,82E+08	1,09E+09	8,99E+08	8,81E+08	7,11E+08	4,51E+08	1,18E+09
P02649	APOE_HUMAN	APOE	Apolipoprotein E	3,66E+08	4,47E+08	2,95E+08	2,63E+08	2,9E+08	4,31E+08	2,92E+08	3,65E+08	3,56E+08	5,92E+08	2,97E+08	3,43E+08	2,35E+08	3,6E+08	4,6E+08	3,66E+08
Q13790	APOF_HUMAN	APOF	Apolipoprotein F	1,72E+08	1,97E+08	1,17E+08	1,21E+08	1,41E+08	1,59E+08	1,64E+08	1,74E+08	1,35E+08	1,13E+08	2,01E+08	1,37E+08	1,37E+08	1,44E+08	1,26E+08	1,64E+08
P02749	APOH_HUMAN	APOH	Beta-2-glycoprotein 1	3,25E+09	3,44E+09	5,89E+09	3,31E+09	3,08E+09	3,66E+09	2,81E+09	2,05E+09	2,72E+09	1,75E+09	3,3E+09	3,89E+09	3,62E+09	1,71E+09	3,37E+09	3,84E+09
O14791	APOL1_HUMAN	APOL1	Apolipoprotein L1	1,24E+08	4,17E+08	1,8E+08	1,52E+08	1,48E+08	2,04E+08	1,77E+08	2,16E+08	3,21E+08	3,46E+08	3,17E+08	1,44E+08	1,32E+08	1,38E+08	1,37E+08	1,46E+08
O95445	APOM_HUMAN	APOM	Apolipoprotein M	3,09E+08	9,18E+08	5,03E+08	2E+08	1,72E+08	3,02E+08	3,4E+08	3,51E+08	3,05E+08	4,02E+08	3,45E+08	2,09E+08	1,89E+08	2,99E+08	2,66E+08	2,93E+08
O95445-2	APOM_HUMAN	APOM	Isoform 2 of Apolipoprotein M	44937800	1,48E+08	90383200	26281900	35439800	45548000	59032000	53280300	61534200	53540300	59181400	14312900	30427400	36775700	38465900	37335600
P05067	A4_HUMAN	APP	Amyloid-beta precursor protein	2585020	4856820	10813900	2856080	1935360	1727160	2406320	3070700	2902630	5144040	5876440	2656480	1053480	1732120	3973830	1921220
P05089	ARGI1_HUMAN	ARG1	Arginase-1	3857610		20894100	2166600	8103280	2264240	4771320	1580560	2151770	2238170	1416710	1425460	1812260	7172010	1512970	4714450
O75882	ATRN_HUMAN	ATRN	Attractin	1,69E+08	3,03E+08	1,5E+08	2,05E+08	2,48E+08	2,37E+08	1,87E+08	2,1E+08	1,7E+08	3,51E+08	2,18E+08	1,85E+08	1,79E+08	2,29E+08	2,27E+08	2,12E+08
P25311	ZA2G_HUMAN	AZGP1	Zinc-alpha-2-glycoprotein	1,05E+09	8,61E+08	7,42E+08	8,03E+08	1,09E+09	8,41E+08	8,48E+08	1,11E+09	6,75E+08	6,74E+08	8,52E+08	1,05E+09	1,02E+09	5,92E+08	6,75E+08	1,42E+09
P61769	B2MG_HUMAN	B2M	Beta-2-microglobulin	1,13E+08	31948400	34722100	1,7E+08	1,12E+08	89832700	63466200	1,28E+08	79942000	26521500	47809000	60334100	1,1E+08	30373700	55521400	2,04E+08
Q9NY97	B3GN2_HUMAN	B3GNT2	N-acetyllactosaminide beta-1,3-N-	981240		631511	737505	646074	864841	773800		268348	650402	768265	610274	741460	851666	730642	771303
P80723	BASP1_HUMAN	BASP1	Brain acid soluble protein 1	562574	373772	1612300	337934	677270	692421	1004500	422824	328685	396655	648857	941126	441527	372206	340075	729725
P06276	CHLE_HUMAN	BCHE	Cholinesterase	22971800	37343900	26839000	31701200	20513300	22997600	24894000	27937000	33957100	43418900	36387800	32225400	21247000	23185800	23927400	23748500
Q13867	BLMH_HUMAN	BLMH	Bleomycin hydrolase	54712300	11573900	98516700	14641600	53259900	12812500	42400900	16053500	11574400	13225800	13060900	11105800	9709350	44746700	14191000	17889500
P30043	BLVRB_HUMAN	BLVRB	Flavin reductase (NADPH)	8271340	8824820	1,47E+09	4151180	7475440	4157740	10270200	8297680	6385630	19320900	6712280	5744450	8798990	7103290	10409900	5550540
Q8TDL5	BPIB1_HUMAN	BPIFB1	BPI fold-containing family B memb	460910	2314190		266546	740882	711704	307327	407628	1937170	916126	1709160	572772	561235	456378		
Q10588	BST1_HUMAN	BST1	ADP-ribosyl cyclase/cyclic ADP-rib	5529840	9210230	23132200	6595440	17842400	9089420	3836040	3538990	7526040	9809420	3652620	10300100	6619690	12660900	8882950	13913000
P43251	BTD_HUMAN	BTD	Biotinidase	30185700	56158800	22002800	41231300	33350600	45196900	44056900	43198500	37692400	45278700	46165800	39116700	25722800	44422600	33688500	35947900
P02745	C1QA_HUMAN	C1QA	Complement C1q subcomponent s	2,29E+08	2,23E+08	3,24E+08	2,3E+08	2,41E+08	2,17E+08	1,97E+08	3,06E+08	1,84E+08	2,23E+08	1,68E+08	2,11E+08	2,13E+08	2,49E+08	2,33E+08	2,09E+08
P02746	C1QB_HUMAN	C1QB	Complement C1q subcomponent s	2,63E+08	2,92E+08	8,44E+08	2,02E+08	3,01E+08	3,92E+08	3,87E+08	4,09E+08	3,64E+08	4,64E+08	2,76E+08	3,19E+08	3,7E+08	2,88E+08	2,44E+08	2,63E+08
P02747	C1QC_HUMAN	C1QC	Complement C1q subcomponent s	2,66E+08	2,64E+08	5,17E+08	2,22E+08	2,96E+08	2,88E+08	2,12E+08	3,55E+08	2,54E+08	2,66E+08	2,44E+08	2,43E+08	2,83E+08	2,72E+08	2,45E+08	2,99E+08
A0A3B3ISR2	A0A3B3ISR2_HUMAN	C1R	complement subcomponent C1r	1,01E+09	1,25E+09	1,21E+09	1,08E+09	1,36E+09	1,35E+09	1,33E+09	1,11E+09	1,35E+09	1,82E+09	1,21E+09	1,24E+09	1,14E+09	1,2E+09	1,22E+09	1,13E+09
Q9NZP8	C1RL_HUMAN	C1RL	Complement C1r subcomponent-l	90423100	62316400	65350600	51409700	95533000	64408800	95127000	1,08E+08	80951900	1,01E+08	76084900	50129600	79672200	84253700	98563300	80296200
P09871	C1S_HUMAN	C1S	Complement C1s subcomponent	3,04E+08	3,44E+08	3,58E+08	3,44E+08	4,12E+08	4E+08	3,64E+08	3,34E+08	3,59E+08	4,59E+08	3,61E+08	3,69E+08	3,37E+08	3,72E+08	3,52E+08	3,44E+08
P06681	CO2_HUMAN	C2	Complement C2	1,11E+08	1,49E+08	1,2E+08	1,19E+08	99021300	1,29E+08	1,26E+08	1,13E+08	1,14E+08	1,11E+08	1,12E+08	1,1E+08	83992600	95086500	1,04E+08	1,41E+08
A0A8Q3SI22	A0A8Q3SI22_HUMAN	C3	Complement C3 (Fragment)	1,7E+08	2,38E+08	2,04E+08	1,89E+08	1,55E+08	1,45E+08	1,76E+08	1,93E+08	1,56E+08	2,68E+08	2,47E+08	2,2E+08	1,63E+08	1,64E+08	1,72E+08	2,21E+08
A0A8Q3SI34	A0A8Q3SI34_HUMAN	C3	Complement C3 (Fragment)	54487800	94704400	82431400	59548300	54210100	1,14E+08	49322500	45278000	82649000	98189800	58629200	73593800	34297500	94361000	89256500	71387700
P01024	CO3_HUMAN	C3	Complement C3	4,65E+09	5,97E+09	5E+09	5,44E+09	4,04E+09	4,38E+09	4,72E+09	5,55E+09	4,95E+09	7,03E+09	5,71E+09	4,96E+09	4,5E+09	5,59E+09	5,07E+09	5,04E+09
P0C0L4	CO4A_HUMAN	C4A	Complement C4-A	1,3E+09	1,96E+09	1,2E+09	1,83E+09	1,68E+09	1,23E+09	1,28E+09	1,1E+09	7,97E+08	2,46E+09	1,85E+09	1,16E+09	7,23E+08	1,27E+09	1,33E+09	1,27E+09
P0C0L5	CO4B_HUMAN	C4B	Complement C4-B	4,26E+08	6,99E+08	5,6E+08	7,2E+08	5,66E+08	2,2E+08	4,87E+08	4,82E+08	41134000	1,13E+09	5,16E+08	3,37E+08	1,31E+08	2,81E+08	3,86E+08	3,16E+08
P04003	C4BPA_HUMAN	C4BPA	C4b-binding protein alpha chain	1,29E+09	2,7E+09	1,9E+09	1,79E+09	1,67E+09	1,97E+09	1,43E+09	2,39E+09	2,14E+09	2,4E+09	2,54E+09	1,66E+09	1,6E+09	1,54E+09	2,79E+09	2,18E+09
P20851	C4BPB_HUMAN	C4BPB	C4b-binding protein beta chain	1,23E+08	1,79E+08	1,89E+08	1,57E+08	1,31E+08	1,81E+08	1,25E+08	1,72E+08	2,21E+08	2,2E+08	2,31E+08	1,69E+08	1,35E+08	1,34E+08	2,2E+08	2,04E+08
A0A8V8TMU5	A0A8V8TMU5_HUMAN	C5	Complement C5 (Fragment)	3,53E+08	2,96E+08	3,01E+08	3,06E+08	3,25E+08	2,51E+08	2,96E+08	2,61E+08	3,11E+08	3,94E+08	3,63E+08	2,72E+08	2,44E+08	3,42E+08	3,96E+08	3E+08
P01031	CO5_HUMAN	C5	Complement C5	3,51E+08	3,69E+08	1,57E+08	3,49E+08	3,47E+08	3,01E+08	3,07E+08	2,64E+08	4,09E+08	4,58E+08	4,26E+08	2,77E+08	2E+08	3,23E+08	4,9E+08	3,16E+08
P13671	CO6_HUMAN	C6	Complement component C6	1,93E+08	97630200	1,81E+08	1,84E+08	1,82E+08	2E+08	1,92E+08	2,04E+08	2,52E+08	2,71E+08	2,11E+08	2,08E+08	1,43E+08	1,83E+08	1,83E+08	1,62E+08
P10643	CO7_HUMAN	C7	Complement component C7	3,11E+08	2,57E+08	1,34E+08	2,22E+08	3,31E+08	3,12E+08	2,43E+08	2,58E+08	1,93E+08	1,69E+08	1,56E+08	1,38E+08	2,22E+08	2,81E+08	2,8E+08	2,98E+08
P07357	CO8A_HUMAN	C8A	Complement component C8 alpha	2,43E+08	2,49E+08	1,81E+08	1,82E+08	2,25E+08	2,3E+08	2,37E+08	3,07E+08	1,92E+08	3,41E+08	3,39E+08	2,36E+08	1,94E+08	1,93E+08	2,6E+08	1,66E+08
P07358	CO8B_HUMAN	C8B	Complement component C8 beta c	69931100	95370000	96484900	72372900	74412200	98306800	75459500	95830300	81697600	1,25E+08	1,14E+08	67173700	78474300	90784900	85047000	68322800
P07360	CO8G_HUMAN	C8G	Complement component C8 gamn	1,36E+08	1,62E+08	1,62E+08	1,32E+08	1,16E+08	1,67E+08	1,45E+08	1,64E+08	1,22E+08	2,2E+08	1,73E+08	1,49E+08	1,34E+08	1,41E+08	1,57E+08	1,12E+08
P02748	CO9_HUMAN	C9	Complement component C9	6,76E+08	4,32E+08	5,49E+08	6,21E+08	7,2E+08	5,72E+08	5,58E+08	6,61E+08	7,86E+08	6,2E+08	7,05E+08	6,86E+08	4,99E+08	5,96E+08	6E+08	5,4E+08
E5RIF9	E5RIF9_HUMAN	CA1	Carbonic anhydrase (Fragment)	16647900	18546600	4,49E+09	3670030	16709100	9700390	14689200	19357500	7240490	27192300	8361760	10690800	19678900	9705100	31196600	3991360
P00915	CAH1_HUMAN	CA1	Carbonic anhydrase 1	53721900	63029900	8,43E+09	16498700	56720200	36733700	62628500	67854000	31977300	1,04E+08	25095000	38892700	49712800	32474300	1,05E+08	14619900
P00918	CAH2_HUMAN	CA2	Carbonic anhydrase 2	2718240	7182640	9,15E+08	2958870	5594010	4079740	7827120	6133380	4471400	11275000	3348820	4879660	4808810	2703560	12430800	1210880
P54289	CA2D1_HUMAN	CACNA2D1	Voltage-dependent calcium channel subunit al		3407550	3963590	2684120	4936800	5266430	3556700		1299910	2769110	2140570	3630120	2961670	2882050	1997400	3018030
Q9NZT1	CALL5_HUMAN	CALML5	Calmodulin-like protein 5	12997400		15426400	5973230	3681060	8007630	10401800	3069620	7780690	3291730	4358540	3481230	2767470	2,68E+08	13953100	10384500
P27797	CALR_HUMAN	CALR	Calreticulin	4652160	2442270	1,13E+08	4310020	7174640	4320270	5491290	5365860	2742930	2938650	3817210	5976130	5203210	11047400	3093100	10140400
P49913	CAMP_HUMAN	CAMP	Cathelicidin antimicrobial peptide	4079060	14072400	39656600	10569400	4453350	3797440	10257100	4213750	2285860	4599940	5999700	3058650	2216490	3218710	7043770	3987220
P31944	CASPE_HUMAN	CASP14	Caspase-14	16362200	6835610	21928100	9090310	14287400	6134680	32020900	6565890	12178200	5015000	5691850	4752540	7050410	24703200	10479800	11921300
P04040	CATA_HUMAN	CAT	Catalase	49678100	17157400	7,07E+08	12306700	23932900	13118400	27970300	14091800	13254100	35479800	16815800	20024900	11992400	13201700	24645300	13506500
Q6YHK3	CD109_HUMAN	CD109	CD109 antigen	475466	1172640	3234290	1719470	1649910	2606050	2022300	1352050	1228260	487525	609684	2262430	893537	1848310	1207440	1787530
P08571	CD14_HUMAN	CD14	Monocyte differentiation antigen C	33100700	27221100	25695900	30682000	33663200	34681900	26518100	31717300	37460000	29155400	28553500	39592700	21941400	25759600	37116800	37075000
Q86VB7	C163A_HUMAN	CD163	Scavenger receptor cysteine-rich t	3476490	1974760	2933860	5417290	5299570	4965730	3259720	6562110	1551160	2837260	1950510	4180460	4061600	3093740	5581340	10133800
P16070	CD44_HUMAN	CD44	CD44 antigen	51919000	46553200	79302000	48586500	54660400	57409800	48207500	40220800	54470800	35772200	41445200	38811200	55521200	40788000	41238000	81982500
P13987	CD59_HUMAN	CD59	CD59 glycoprotein	4057270	2024650	79826100	2982420	2847140	1700510	2212650	2103000	1351860		1409510	2143020	3109910	2302790	1740750	4773200
O43866	CD5L_HUMAN	CD5L	CD5 antigen-like	4,81E+08	3,41E+08	2,66E+08	2,82E+08	3,05E+08	3,44E+08	8,76E+08	9,38E+08	5,48E+08	4,14E+08	3,4E+08	6,04E+08	1,96E+08	2,65E+08	2,88E+08	4,6E+08
Q9NPY3	C1QR1_HUMAN	CD93	Complement component C1q rece	2619260	895085	2894730	2124960	4354400	2872920	1873170	2151770	1394730	915620	1833120	2178020	2995200	3174530	1916070	6189720
P12830	CADH1_HUMAN	CDH1	Cadherin-1	2048510	1043150	1860800	2069040	1673740	4539360	2569710	2921750	1226640	1242920	1432370	2873470	2459490	1662990	1335630	5153150
P55290	CAD13_HUMAN	CDH13	Cadherin-13	2048750	3497720	3594220	2133530	3458070	3716000	3207680	1936240	2196680	3185420	2326050	3423490	2803870	3641980	1540670	3633220
P33151	CADH5_HUMAN	CDH5	Cadherin-5	28447800	21487200	29100000	35413000	49374600	29366200	27031500	25788300	16664700	16607100	20798000	19597400	42725000	56181300	23400600	39105600
P11597	CETP_HUMAN	CETP	Cholesteryl ester transfer protein	20686200	14203700	23401400	8624730	17469200	14031500	18063500	15260000	9184340	15778100	19244800	16633000	23466700	26845600	17280300	29747400
P00746	CFAD_HUMAN	CFD	Complement factor D	1,51E+08	43248300	56223800	1,62E+08	1,19E+08	1,17E+08	98645400	1,54E+08	46162400	29999700	59886700	81776200	2,02E+08	72630100	71690300	1,59E+08
A0A8Q3SI99	A0A8Q3SI99_HUMAN	CFH	Complement factor H	5,02E+08	4,24E+08	4,08E+08	5,66E+08	5,56E+08	4,71E+08	4,23E+08	4,45E+08	4,3E+08	5,41E+08	5,11E+08	4,73E+08	3,47E+08	3,92E+08	4,55E+08	5,45E+08
Q03591	FHR1_HUMAN	CFHR1	Complement factor H-related prot	2,27E+08	82829200	75895800	1,96E+08	2,14E+08	1,75E+08	1,69E+08	79927500	1,22E+08	96488800	2,13E+08	2,57E+08			1,09E+08	1,64E+08
P36980	FHR2_HUMAN	CFHR2	Complement factor H-related prot	1,86E+08	1,27E+08	89321400	2,45E+08	2,13E+08	1,66E+08	1,41E+08	2E+08	85719800	2,07E+08	2,01E+08	2,06E+08	1,45E+08	1,1E+08	2,19E+08	2,73E+08
Q92496	FHR4_HUMAN	CFHR4	Complement factor H-related prot	22185000	16829100	14514800	40333000	24463000	36090700	28304800	35835700	16472300	41542600	91119300	21250100	20618400	27520600	36565600	30418300
Q9BXR6	FHR5_HUMAN	CFHR5	Complement factor H-related prot	1,92E+08	1,09E+08	1,08E+08	1,82E+08	1,71E+08	1,81E+08	1,67E+08	1,08E+08	1,37E+08	1,47E+08	1,96E+08	1,77E+08	41033500	25732300	1,6E+08	1,3E+08
P05156	CFAI_HUMAN	CFI	Complement factor I	3,03E+08	3,55E+08	2,26E+08	3,74E+08	2,84E+08	3,11E+08	3,33E+08	3,22E+08	3,73E+08	4,19E+08	4,45E+08	2,98E+08	2,62E+08	3E+08	3,07E+08	2,79E+08
P23528	COF1_HUMAN	CFL1	Cofilin-1	1757620	4290430	2,07E+08	3986830	6656290	1730850	5833670	3131220	2073020	5335660	2837820	1662910	1430890	7964290	2842690	2881450
P27918	PROP_HUMAN	CFP	Properdin	29759500	35807900	35842600	24748800	17399300	23187800	20181200	41737000	29240400	38005400	26562000	26258000	22223000	35404000	21940900	35007800
P10645	CMGA_HUMAN	CHGA	Chromogranin-A	4562110		1516210	10749100	6051920	1538190	1493920	41949100	5541440		1799030	2891740	1406660	3315490	1232180	20998600
O00533	NCHL1_HUMAN	CHL1	Neural cell adhesion molecule L1-l	10083100	9359300	6807550	7337910	10774800	12577900	9418620	7252800	9258270	6803120	8563160	9284380	12769200	17122900	7754630	15122400
P05452	TETN_HUMAN	CLEC3B	Tetranectin	1,81E+08	1,84E+08	2,16E+08	1,2E+08	1,9E+08	1,89E+08	2,33E+08	1,65E+08	1,96E+08	1,73E+08	1,87E+08	1,78E+08	1,91E+08	2,11E+08	1,4E+08	1,76E+08
P10909-6	CLUS_HUMAN	CLU	Isoform 6 of Clusterin	8,54E+08	1,08E+09	1,03E+09	8,53E+08	1E+09	1,11E+09	8,83E+08	9,28E+08	8,99E+08	1,06E+09	8,53E+08	7,79E+08	8,96E+08	9,37E+08	7,96E+08	1,04E+09
Q96KN2	CNDP1_HUMAN	CNDP1	Beta-Ala-His dipeptidase	23701200	66706400	24266800	22105400	21835100	20933000	41789900	36081900	22233900	60700100	33115200	42589200	49439500	6122430	32623100	14109700
P39060	COIA1_HUMAN	COL18A1	Collagen alpha-1(XVIII) chain	4508180	2941630	4547950	5735280	4922020	3137870	3308630	4932090	5930920	2199710	2477930	1671440	5006420	3128730	3384540	4695340
P12109	CO6A1_HUMAN	COL6A1	Collagen alpha-1(VI) chain	633211	819956	567839	453039	1267420	1037290	929803	303990	446269	836108	625436	850093	688310	878986	1205330	1154870
P12111	CO6A3_HUMAN	COL6A3	Collagen alpha-3(VI) chain	1932650	1478000	1685690	1541900	2377070	2188120	2339540	1162010	907270	582921	1769770	1458150	2345110	2336850	1328510	3811700
Q9Y6Z7	COL10_HUMAN	COLEC10	Collectin-10	13346800	8141470	8089270	8842390	24990800	10491600	8866430	8538140	7370130	7035980	6818970	12371900	7994750	16695300	17766300	13299500
Q9BWP8	COL11_HUMAN	COLEC11	Collectin-11	7784720	7957440	9481750	8113120	17256900	8209450	5346130	5714910	7487260	6941380	3961280	7649060	5184750	13134000	12579800	6335830
P49747	COMP_HUMAN	COMP	Cartilage oligomeric matrix proteir	18206100	13642400	16648400	11288100	14715300	25988300	19633600	10247000	4385750	7905380	12048600	12149100	19209000	18712400	13673600	34705100
P31146	COR1A_HUMAN	CORO1A	Coronin-1A	975573	1152810	45246400	1033230	1523940	1080810	3314660	885438	739358	1995960	1204320	1194590	656440	1070410	891376	718653
P00450	CERU_HUMAN	CP	Ceruloplasmin	2,74E+09	2,25E+09	2,61E+09	2,38E+09	3,62E+09	2,55E+09	2,2E+09	2,35E+09	2,59E+09	1,9E+09	2,68E+09	2,34E+09	2,4E+09	2,49E+09	3,26E+09	2,99E+09
Q96IY4	CBPB2_HUMAN	CPB2	Carboxypeptidase B2	73417600	1,09E+08	74824500	77230100	70105400	92274600	86444000	85287900	75439200	97013500	95390500	1,06E+08	78348900	76686600	78489100	97975800
P15169	CBPN_HUMAN	CPN1	Carboxypeptidase N catalytic chai	1,1E+08	1,47E+08	1,1E+08	74406700	74423000	1,31E+08	88166000	1,09E+08	1,24E+08	1,53E+08	1,32E+08	1,01E+08	86959200	1,06E+08	67755400	65638800
P22792	CPN2_HUMAN	CPN2	Carboxypeptidase N subunit 2	2,5E+08	4,03E+08	2,51E+08	2,5E+08	2,11E+08	3E+08	1,93E+08	2,46E+08	2,68E+08	3,8E+08	2,99E+08	2,66E+08	2,48E+08	2,75E+08	1,84E+08	1,77E+08
P54108	CRIS3_HUMAN	CRISP3	Cysteine-rich secretory protein 3	14703600	9516790	11824500	17359600	10566500	12403800	9460580	10273000	6202510	8831240	14877400	18855000	7399540	12401300	9887830	9068240
P02741	CRP_HUMAN	CRP	C-reactive protein	29308200	3044380	10404000	90967000	29668700	2892420	11437100	25598300	86249500	3,44E+08	98257000	1,09E+08	4329460	22638100	1,6E+08	17101400
Q9NQ79	CRAC1_HUMAN	CRTAC1	Cartilage acidic protein 1	16737900	26191800	22828400	20809300	14899100	24651600	23139700	6577510	16768100	13542800	14246100	15754600	13706600	13628900	16717200	28939600
P07333	CSF1R_HUMAN	CSF1R	Macrophage colony-stimulating fac	2453250	1537970	2685350	3604660	4299790	4693210	2611760	3864350	1371730	1463930	1271960	2528410	3202420	3207040	3884420	4831830

P01034	CYTC_HUMAN	CST3	Cystatin-C	99832400	28631900	58203300	1,04E+08	98655300	77989300	65709700	1,04E+08	41581900	28142600	41996400	60771700	1,22E+08	52724000	42438200	1,41E+08
Q15828	CYTM_HUMAN	CST6	Cystatin-M	8502700	2279280	3752260	3884020	5953450	5356300	5103160	5664320	2279430	1813440	3257470	2365070	5879690	6748200	2409810	13102700
Q01459	DIAC_HUMAN	CTBS	Di-N-acetylchitobiase	1719640	2553420	2442450	1983450	2646850	2046650	1594110	1900320	1804420	2597030	2480560	2039020	1924770	2005460	1513280	2272200
P07858	CATB_HUMAN	CTSB	Cathepsin B	1470360		4371310	2169940	3961240	2042370	2515030	2226790	758816	828840	1044250	2693260	3664070	3122230	3896360	2858100
P07339	CATD_HUMAN	CTSD	Cathepsin D	7700720	7028400	7292960	9935450	6934790	6511500	6968400	3325310	5486760	7619790	5787300	7627420	2825090	23115200	7733390	7431850
P08311	CATG_HUMAN	CTSG	Cathepsin G	4030370	1566960	38496400	4698660	4979090	2042070	4708390	2435420	3391500	4421230	3767270	2556100	1995810		4209530	3150170
P09668	CATH_HUMAN	CTSH	Pro-cathepsin H	459563	391112		280331	516752	412613	687536	236336	301526	579731	219596	187286	246877	476082	257997	
Q14118	DAG1_HUMAN	DAG1	Dystroglycan 1	2751450	663839	1735500	2874540	2616120	2219200	2418810	1803250	869085	874677	1347430	1847080	3571200	1282710	1433800	5372700
P09172	DOPO_HUMAN	DBH	Dopamine beta-hydroxylase	13352400	4823490	3279750	6922750		9096130	3110830	18312400	10704800	28107300	3013580	5234200	16887700		2414390	19714600
P81605	DCD_HUMAN	DCD	Dermcidin	8866630	7398870	1,24E+08	8026930	13508500	13114400	7149310	12193900	15005700	11854900	15118000	11096200	9675590	12496700	4668250	9969240
P59666	DEF3_HUMAN	DEFA3	Neutrophil defensin 3	32276800	19811500	7,08E+08	78285600	48707000	14588100	24111600	49663800	12584000	29242900	23827200	18831200	28333800	10967500	28406900	47032700
Q6E0U4	DMKN_HUMAN	DMKN	Dermokine	1120670	1098530	737094	541052	605359	975150	1399050	890384	457816	391066	1146390	379750	1141670	832245	558876	1584700
Q08554	DSC1_HUMAN	DSC1	Desmocollin-1	17952500	6360210	23256500	6435070	20767900	7089900	10426400	6059230	6363100	5964600	6528060	5077990	8289590	15128000	7024300	16187200
Q02413	DSG1_HUMAN	DSG1	Desmoglein-1	73689100	28597400	71314100	31580000	94429700	35129700	50364800	28795900	32181600	32692200	26015500	17391700	48282100	93130900	36919800	53236900
Q14126	DSG2_HUMAN	DSG2	Desmoglein-2	2403440		2530040	2414720	2745330	3199550	973553	1937350			1170060	1883910	2723840	1558190	1218720	3234370
P15924	DESP_HUMAN	DSP	Desmoplakin	19756500	6076830	33823900	9992550	24259700	12041500	13832300	9003710	8500860	7707460	7833180	6464760	8912520	41879400	11582800	14363200
Q16610	ECM1_HUMAN	ECM1	Extracellular matrix protein 1	46116500	29259200	52384900	33396400	35255300	34289900	35285900	28601800	21032500	37170900	23594100	29250800	15886300	69445300	38751500	44491500
Q12805	FBLN3_HUMAN	EFEMP1	EGF-containing fibulin-like extrace	32254500	11021900	28903300	27575400	43520400	25871300	29972500	20256400	14191900	8907170	20487600	25712200	47025800	36553500	32804400	64027400
P00533	EGFR_HUMAN	EGFR	Epidermal growth factor receptor		55786	52361.1	22295.3	49975.8	24920.7	32036.9	47690.8	21906.9	63630.9	40140.5	59056.1		54486.6	30330.3	41816.3
I3L397	I3L397_HUMAN	EIF5A	Eukaryotic translation initiation fac	236224	511288	1,18E+08	235026	385810	294376	260945	327855	118069	929115	228473	199154	307826	1919230	662554	251214
Q9ULC0	MUCEN_HUMAN	EMCN	Endomucin	307581	302524		504888	648003	293854	626418	560126	304938	287664	372158	440444		330373	421053	1184390
P06733	ENOA_HUMAN	ENO1	Alpha-enolase	5459630	3642430	1,67E+08	1129930	5067370	4045890	15177800	2659330	3810610	4244120	3693890	5060120	31806500	55951700	4556310	3412070
Q13822	ENPP2_HUMAN	ENPP2	Autotaxin	934320	738654	630174	1238860	834449	1173200	908130	949297	751958	561790	709408	297928	1406860	1559550	1068140	1327430
P00742	FA10_HUMAN	F10	Coagulation factor X	29681000	88334700	85181200	58760000	60542500	80706300	66285200	55189700	75098600	85117800	95951200	63392900	49827000	53810800	54937500	64699800
P03951	FA11_HUMAN	F11	Coagulation factor XI	57226900	44005700	58954100	45741600	48122400	52835100	39820300	53777900	60020200	68471800	46171500	40298200	34158200	25698500	51545000	36293300
A0A8Q3WL37	A0A8Q3WL37_HUMAN	F12	Coagulation factor XII	29446400	33171100	16282500	17750500	20184500	35017500	35065500	26637100	28514100	42443700	48728000	28015100	13092100	32078300	35081500	36954200
P00748	FA12_HUMAN	F12	Coagulation factor XII	3,65E+08	4E+08	2,64E+08	2,38E+08	2,12E+08	3,81E+08	3,63E+08	3,04E+08	3,62E+08	4,87E+08	4,46E+08	3,09E+08	1,46E+08	3,77E+08	3,76E+08	3,82E+08
P00488	F13A_HUMAN	F13A1	Coagulation factor XIII A chain	16294800	17592200	2,67E+08	10120200	9960970	16941400	7229250	9057180	6980080	12375200	14256400	10726900	14634700	25910600	9467280	23729100
P05160	F13B_HUMAN	F13B	Coagulation factor XIII B chain	51178800	66709500	58162700	47811500	52558600	54947500	44073600	48012700	39991300	72223400	75957200	42716000	51717900	53004000	55100600	60109000
P00734	THRB_HUMAN	F2	Prothrombin	4,45E+08	9,57E+08	1,15E+09	7,24E+08	6,84E+08	4,04E+08	6,5E+08	7,53E+08	8,54E+08	1,08E+09	8,69E+08	7,41E+08	5,92E+08	7,05E+08	8,04E+08	7,72E+08
P12259	FA5_HUMAN	F5	Coagulation factor V	17857700	22776200	64766900	30093800	19498700	20657100	29014500	26196800	42341900	31975400	29921400	20137600	27464700	24592400	19238900	29465500
P08709	FA7_HUMAN	F7	Coagulation factor VII	1716600	4232160	4512370	5014070	3910000	4685290	5805850	4852290	4466180	4687680	4907610	3200510	4813890	3479180	3384370	5596430
P00740	FA9_HUMAN	F9	Coagulation factor IX	32637600	72583500	60950200	68815300	47835300	57785100	66367300	68195600	60467800	77501600	66229100	79721000	46678400	46363900	53088300	58065500
P23142	FBLN1_HUMAN	FBLN1	Fibulin-1	68184600	36860900	52508500	52466600	1,43E+08	89287300	51808700	50169700	44459500	48765000	44062400	73687300	88754000	1,18E+08	68736100	1,07E+08
P23142-4	FBLN1_HUMAN	FBLN1	Isoform C of Fibulin-1	66156200	25317900	36758000	47496300	1,06E+08	63926800	51364300	37065500	33816100	31350100	37549200	63270400	80417500	83538600	41739000	82911300
Q9UBX5	FBLN5_HUMAN	FBLN5	Fibulin-5	177944		352978	147346	274149	100808	98338.5	122072			87019.2	116599	125158	83863.5	155320	316912
P35555	FBN1_HUMAN	FBN1	Fibrillin-1	2271840	1479910	1559070	1573120	2784450	2392400	1253540	1602620	1315570	1432980	1406390	2197500	2339640	1912380	3010540	2989090
Q9Y6R7	FCGBP_HUMAN	FCGBP	IgGFc-binding protein	37637100	40596700	21751900	87947800	90415900	71324700	1,14E+08	76398200	54727600	27116600	40887500	59057800	73253300	62264100	59143100	1,07E+08
P08637	FCG3A_HUMAN	FCGR3A	Low affinity immunoglobulin gamm	8839250	8214390	9768030	5791190	12616700	6306760	6494100	8981820	5488920	4322460	3854360	6265330	9363920	11321100	7381060	9315950
O00602	FCN1_HUMAN	FCN1	Ficolin-1	108060	108926	1023160	140963	262542	126722	77484.1		94641	153138	180539	134089	75791.3		140106	118724
Q15485	FCN2_HUMAN	FCN2	Ficolin-2	23800600	22160400	8649230	14423800	25997100	7312790	10183100	10115300	24090900	13867200	24110800	14087900	8357870	15648700	9842120	12621600
O75636	FCN3_HUMAN	FCN3	Ficolin-3	1,23E+08	1,53E+08	83657400	87947300	52048100	1,17E+08	1,09E+08	1,15E+08	1,93E+08	2,42E+08	1,65E+08	1,26E+08	78705700	84822800	51417500	1,35E+08
Q9UGM5	FETUB_HUMAN	FETUB	Fetuin-B	70352800	76561600	69391900	51775300	65365300	57689100	56353000	47034300	63759000	46966500	45163200	45260600	77599800	89240200	1,29E+08	74859400
P02671	FIBA_HUMAN	FGA	Fibrinogen alpha chain	9,14E+08	3,12E+08	3,72E+10	6,09E+08	4,86E+08	7,06E+08	7,29E+08	3,61E+08	5,19E+09	3,29E+08	3,63E+08	4,22E+08	4,15E+08	2,43E+08	4,29E+08	6,76E+08
P02675	FIBB_HUMAN	FGB	Fibrinogen beta chain	61428700	10882400	9,2E+09	26196000	17172000	24726600	18206200	9132900	29024900	14329600	13530200	33084000	10154300	25574400	19491400	39665800
P02679	FIBG_HUMAN	FGG	Fibrinogen gamma chain	61688300	6775290	5,19E+09	25377200	16672900	25243700	16978600	9463130	25283700	11694800	13546800	35958200	11079000	18591800	18503200	43042100
P20930	FILA_HUMAN	FLG	Filaggrin	2330560	462923	7155750	1263250	2908360	1053780	2119150	1498480	1557460	501031	1318730	1098350	1343030	5142400	1043950	3162630
Q5D862	FILA2_HUMAN	FLG2	Filaggrin-2	44950600	10385200	46433300	17252500	42880700	14275200	24446300	13911300	18694300	10985700	8682640	7762060	12918900	25044500	10853900	27606600
P21333	FLNA_HUMAN	FLNA	Filamin-A	10433200	18397200	5,18E+09	11393400	12682100		16249800		13659100	15595600	26422800	7438690	6353990	10401900	8278730	17893500
P02751-1	FINC_HUMAN	FN1	Isoform 1 of Fibronectin	6,55E+08	9,81E+08	3,6E+08	5,76E+08	4,72E+08	4,17E+08	6,86E+08	5,2E+08	4,78E+08	9,59E+08	6,21E+08	7,52E+08	3,12E+08	9,65E+08	7,61E+08	7,98E+08
Q12841	FSTL1_HUMAN	FSTL1	Follistatin-related protein 1	261720	256118	282314	222000	433678	290663	394341	274169	191903	261669	283034	305396	251439	265693	315516	410461
Q14697	GANAB_HUMAN	GANAB	Neutral alpha-glucosidase AB	285788		5153040	356415	428321	476384	487918	267676	267327	244010		371491		530067		568312
P04406	G3P_HUMAN	GAPDH	Glyceraldehyde-3-phosphate dehy	17202800	7814440	1,07E+09	9959000	23033900	8812300	44635700	6753190	10321100	14144500	10256100	9120640	10688700	83155400	11076600	11715000
P02774	VTDB_HUMAN	GC	Vitamin D-binding protein	2,16E+09	2,06E+09	2,04E+09	1,84E+09	1,9E+09	2,24E+09	1,81E+09	2,44E+09	2,1E+09	2,3E+09	2,43E+09	1,83E+09	1,64E+09	2,04E+09	2E+09	2,4E+09
O75223	GGCT_HUMAN	GGCT	Gamma-glutamylcyclotransferase	9028390		43728300		9807270	7071640	9489110		4138690		2183560	3100800	2360610	17833800	6453850	6833350
Q92820	GGH_HUMAN	GGH	Gamma-glutamyl hydrolase	6630110	5826240	4751470	4099340	5711220	6501660	4910160	3922090	8990900	9601710	6457770	5383610	4469460	8475480	8188330	4552910
P07359	GP1BA_HUMAN	GP1BA	Platelet glycoprotein Ib alpha chair	31184900	47117600	1,32E+08	33439400	38986300	21712500	28526000	27136800	26893600	36287600	42508800	25317700	19674200	18819800	28375300	38325500
P40197	GPV_HUMAN	GP5	Platelet glycoprotein V	8121880	27844100	49445400	10870100	5162000	4768230	14277200	13479100	10896500	22689200	20945100	15455600	3602560	4629270	11755900	7904640
P80108	PHLD_HUMAN	GPLD1	Phosphatidylinositol-glycan-specif	35752200	1,2E+08	70570600	33884300	45504400	65074000	52780200	41972100	38837700	71271200	56965500	74146600	24197800	55190000	81294800	60141100
P22352	GPX3_HUMAN	GPX3	Glutathione peroxidase 3	99370800	1,81E+08	1,57E+08	55551300	1,13E+08	1,15E+08	1,09E+08	61213300	1,15E+08	1,98E+08	1,43E+08	1,73E+08	1,17E+08	1,68E+08	1,06E+08	72531500
A0A0A0MT01	A0A0A0MT01_HUMAN	GSN	Gelsolin	5019630	2395270	5874310	3768360	4947520	3959010	3655020	5357800	1824130	3358650	5483280	4753960	5184660	4780400	3843240	4032650
A0A8V8TND7	A0A8V8TND7_HUMAN	GSN	Gelsolin	5441600	2701550	6339890	4896260	4906890	6387110	4079260	5143420	3955120	5647820	4939130	5485840	6307870	7393700	4247660	4513750
P06396-2	GELS_HUMAN	GSN	Isoform 2 of Gelsolin	6,54E+08	5,87E+08	9,44E+08	4,76E+08	6,71E+08	6,96E+08	5,32E+08	5,89E+08	4,19E+08	4,93E+08	5,84E+08	5,42E+08	7,31E+08	5,98E+08	4,35E+08	6,7E+08
P06396	GELS_HUMAN	GSN	Gelsolin	2,29E+08	1,96E+08	2,07E+08	2,5E+08	2,44E+08	2,98E+08	1,95E+08	2,38E+08	2,08E+08	1,9E+08	2,29E+08	1,78E+08	1,89E+08	2,29E+08	1,93E+08	2,65E+08
P00390	GSHR_HUMAN	GSR	Glutathione reductase, mitochond	5623730	3647700	32744300		3693730	2971710			2091430	3183510	2799660	3636480	2222070	2437170	2522780	3800590
P0C0S8	H2A1H_HUMAN	H2AC11	Histone H2A type 1	4696600	4748020	2,48E+08		5609460	4520980	4296260	7091720	3498340	7584560	7324040	13248800	4213830	82680900		3723700
P16104	H2AX_HUMAN	H2AX	Histone H2AX	11793100		2,53E+08		13214100	7866400	13930000	10175500	8298050	17548900	9166520	18988800	6916220	1,63E+08	7083870	7387940
O60814	H2B1C_HUMAN	H2BC12	Histone H2B type 1-K	3445050	3818150	3,33E+08	1049120	3758680	5563590	21666500	3388380	3118820	8128480	4883820	8281660		92603600	1800960	
P62805	H4_HUMAN	H4C1	Histone H4	6878520	5566310	4,27E+08		5757260	3920120	30391800	6834900	4246080	10598000	6496480	11210200	1872920	26563900	3565430	
Q14520	HABP2_HUMAN	HABP2	Hyaluro-binding protein 2	1,59E+08	1,78E+08	1,6E+08	1,34E+08	1,84E+08	1,73E+08	1,48E+08	1,49E+08	1,35E+08	1,91E+08	1,41E+08	96206400	1,14E+08	1,31E+08	1,91E+08	1,75E+08
G3V1N2	G3V1N2_HUMAN	HBA1	Alpha-globin	1,79E+09	3,26E+09	1,59E+10	1,41E+09	2,19E+09	1,91E+09	3,19E+09	2,99E+09	2,28E+09	5,15E+09	2,47E+09	3,12E+09	2,65E+09	2,3E+09	3,39E+09	9,88E+08
P69905	HBA_HUMAN	HBA1	Hemoglobin subunit alpha	63313000	2,63E+08	8E+09	1,99E+08	2,44E+08	1,44E+08	2,78E+08	2,78E+08	2,7E+08	9,54E+08	2,75E+08	1,97E+08	1,93E+08	1,09E+08	4,14E+08	77614600
A0A2R8Y7C0	A0A2R8Y7C0_HUMAN	HBA2	Hemoglobin subunit alpha 2 (Fragn	1,8E+09	2,25E+09	8,54E+10	9,6E+08	2,16E+09	7,53E+08	2,3E+09	1,81E+09	1,29E+09	4,14E+09	1,84E+09	1,96E+09	1,65E+09	1,17E+09	3,1E+09	7,53E+08
P68871	HBB_HUMAN	HBB	Hemoglobin subunit beta	3E+09	4,46E+09	7,14E+10	2,5E+09	3,05E+09	2,61E+09	3,7E+09	4,02E+09	3,58E+09	8,87E+09	3,46E+09	3,06E+09	2,33E+09	2,53E+09	6,91E+09	1,15E+09
P02042	HBD_HUMAN	HBD	Hemoglobin subunit delta	2,95E+09	4,58E+09	3,01E+11	2,56E+09	3,57E+09	2,77E+09	4,93E+09	4,32E+09	3,27E+09	8,58E+09	3,81E+09	4,33E+09	3,52E+09	3,09E+09	6,12E+09	1,49E+09
A0AAG2TV02	A0AAG2TV02_HUMAN	hCG_2003024	HCG2003024	17419900		12563400	15552800	22288100	20717500		17520800	11413000	30463800	4517390	9307950	11363800	20345200	17956400	56018000
Q9ULI3	HEG1_HUMAN	HEG1	Protein HEG homolog 1	3300780	2598510	2620630	2872890	4487850	3454200	3147500	3504460	2256800	1419360	3086260	2641060	3439010	2604000	2819400	5438630
Q04756	HGFA_HUMAN	HGFAC	Hepatocyte growth factor activator	36285100	66758800	33998200	34388900	35817200	39185900	52648300	36843900	27487900	47978100	42294700	26937800	35158500	25547700	36921600	50112200
D5H3J5	D5H3J5_HUMAN	HLA-B	HLA class I histocompatibility antig	622812		25440700	1283880	1700760	828381	1107330	4029330		1035180		820896	1039680	2001920	422470	2108200
P00738	HPT_HUMAN	HP	Haptoglobin	1,5E+08	1,63E+10	1,55E+10	2,1E+10	1,49E+10	1,53E+10	9,34E+09	2,37E+10	1,94E+10	1,74E+10	1,9E+10	1,6E+10	8,07E+09	7,5E+09	9,92E+09	1,65E+10
P00739	HPTR_HUMAN	HPR	Haptoglobin-related protein	4,85E+08	2,17E+09	8,19E+08	4,13E+08	5,22E+08	7,39E+08	8,73E+08	7,9E+08	1,25E+09	1,23E+09	1,43E+09	4,79E+08	5,26E+08	4,17E+08	3,75E+08	3,02E+08
P00739-2	HPTR_HUMAN	HPR	Isoform 2 of Haptoglobin-related pı	57070,7	1272520	392725	134205		284438	128643		218002	111044	269122	69759.7	338855	81511.9	242189	115660
Q9Y251	HPSE_HUMAN	HPSE	Heparanase	267187	369074	1253270	193202			206680		388320	390514	324304		161020	508695	206328	175746
P02790	HEMO_HUMAN	HPX	Hemopexin	4,57E+09	8,69E+09	1,05E+10	1,14E+10	8,7E+09	9,9E+09	1,2E+10	1,09E+10	8,87E+09	9,7E+09	1,11E+10	1,19E+10	1,01E+10	9,47E+09	1,01E+10	8,9E+09
P04196	HRG_HUMAN	HRG	Histidine-rich glycoprotein	8,08E+08	1,37E+09	1,38E+09	1,21E+09	7,41E+08	1,19E+09	1,49E+09	1,08E+09	1,16E+09	8,42E+08	9,5E+08	1,21E+09	1,06E+09	1,47E+09	1,38E+09	9,67E+08
Q86YZ3	HORN_HUMAN	HRNR	Hornerin	53395900	16082400	67456300	29260200	60891400	28100900	41125900	31668800	18998600	20199700	56655200	24751700	35142300	21836400	30011600	32339100
P14625	ENPL_HUMAN	HSP90B1	Endoplasmin	3343210	4778060	44696900	3910580	5703260	3679880	5855400	3620850	4418100	3622300	3709970	4387100	4008780	7816980	1564060	7004430
P34931	HS71L_HUMAN	HSPA1L	Heat shock 70 kDa protein 1-like	1290760	740552	39542100	733826	7921500	966077	4887480	802294		1510510	1017330	1558630		13887600		
P11021	BIP_HUMAN	HSPA5	Endoplasmic reticulum chaperone	30070200	43974000	3,24E+08	34315700	42773000	41485700	40958000	26274800	25568000	27639400	38832200	28759600	31124300	1,04E+08	26519900	35992000
P11142	HSP7C_HUMAN	HSPA8	Heat shock cognate 71 kDa proteir	2482460	2312620	2,57E+08		2214250	2262340	5061360		1193050	3473010	2215400	2043470	2976180	22760400	2213280	
P98160	PGBM_HUMAN	HSPG2	Basement membrane-specific hep	6648680	3988340	4180400	4930970	7648750	7430180	5711200	4228290	3173220	3902240	3876980	7894110	7073660	7792140	5274470	10109600
Q9Y4L1	HYOU1_HUMAN	HYOU1	Hypoxia up-regulated protein 1	1278570	668968	2503260	1015880	1639600	1375300	1709620	1375450	705213	506979	994178	1277170	1655480	683485	434104	1987310
P05362	ICAM1_HUMAN	ICAM1	Intercellular adhesion molecule 1	5535090	3888320	8290740	6399590	9941200	6483090	5379430	6000860	8119500	4835860	4720840	4817030	5611200	10328800	4885030	7618120
P13598	ICAM2_HUMAN	ICAM2	Intercellular adhesion molecule 2	2108940	4921490	11723300	4467660	7506620	3365790	3260540	2643640	2817450	3222290	2306190	3957590	3097930	4835830	4436980	5661920
O75874	IDHC_HUMAN	IDH1	Isocitrate dehydrogenase [NADP] c	332784	774928	6786000		605252	516731	645139		542099	429742		2294440	248124	1453560	306266	458181
P01344	IGF2_HUMAN	IGF2	Insulin-like growth factor 2	45838800	66559400	17903300	35292100	35869100	31106700	27258700	44699700	43981100	51878800	41080900	29216300	21937100	42184200	39776400	50550500
P11717	MPRI_HUMAN	IGF2R	Cation-independent mannose-6-pl	452564	1054420	1267960		1111870	832475	606195	314768	483177	648239	623397	632403	893424	544652	305890	877577
P35858	ALS_HUMAN	IGFALS	Insulin-like growth factor-binding p	51386400	98514300	78884300	26749200	33416600	26838500	50965400	70357300	90152500	1,12E+08	96272300	37035400	28701100	58366900	37806600	34547000
P18065	IBP2_HUMAN	IGFBP2	Insulin-like growth factor-binding p	33275900	5615860	7739850	20450200	18701200	21708400	18446100	15855900	13929200	4608670	10110900	5770930	12715400	8514000	17097800	27541200
P17936	IBP3_HUMAN	IGFBP3	Insulin-like growth factor-binding p	14093600	24333700	17230000	9087770	21487200	11936800	18722100	23443000	21352600	23021300	19258400	14852400	11115600	19069400	19376900	16841500
P22692	IBP4_HUMAN	IGFBP4	Insulin-like growth factor-binding p	10725200	4744130	3376220	8444190	11267100	8848310	5878510	11545500	4518940	4276190	5580830	7463300	10761500	5981390	6284180	13688200
P24593	IBP5_HUMAN	IGFBP5	Insulin-like growth factor-binding p	860479	1063800	602143	251639		454442	690362	908482	307632	666594	1018410	395878	216086	272366	369129	496347
P24592	IBP6_HUMAN	IGFBP6	Insulin-like growth factor-binding p	18149500	5419720	9947950	14441800	14367000	9600040	11595600	16629500	5246340	4880900	7158840	13796800	23270000	6112220	5872830	31483000
Q16270	IBP7_HUMAN	IGFBP7	Insulin-like growth factor-binding p	4417030	1747380	2657760	2452410	5444990	3704460	2733910	2750280	1608080	1406250	1584710	2152830	4828970	2708970	2745280	6317350
P01876-1	IGHA1_HUMAN	IGHA1	Isoform 1 of Immunoglobulin heavy	6,46E+09	1,09E+10	1,83E+10	1,09E+10	1,9E+10	1,2E+10	1,1E+10	1,45E+10	7,52E+09	8,68E+09	1,35E+10	9,55E+09	1,01E+10	1,46E+10	9,16E+09	2,13E+10
A0A0G2JMB2	A0A0G2JMB2_HUMAN	IGHA2	Immunoglobulin heavy constant al	8,27E+08		8,87E+08	4,99E+08	1,81E+09	1,49E+09	9,07E+08	1,14E+09	3,76E+08	1,04E+09	1,11E+09	1,22E+09	1,46E+09	2,22E+09	4,63E+08	2,51E+09
P01877-1	IGHA2_HUMAN	IGHA2	Isoform 1 of Immunoglobulin heavy	6,63E+09	1,71E+10	3,77E+09	1,16E+10	1,88E+10	8,91E+09	9,2E+09	1,37E+10	5,62E+09	1,49E+10	1,01E+10	8,32E+09	1,11E+10	1,24E+10	1,5E+10	2,69E+10
P01880	IGHD_HUMAN	IGHD	Immunoglobulin heavy constant de	1,73E+08	6,72E+08	1,22E+08	56130400	1,28E+08	81859100		4,05E+08	40231100	1,47E+08	1,75E+08	28357200	4303490	2,45E+08	1,43E+08	77945700
P01857-1	IGHG1_HUMAN	IGHG1	Isoform 1 of Immunoglobulin heavy	3,66E+10	2,98E+10	3,58E+10	2,65E+10	2,7E+10	2,24E+10	2,54E+10	3,24E+10	2,18E+10	1,91E+10	2,14E+10	3,81E+10	3,18E+10	3,94E+10	1,73E+10	2,66E+10
P01859-1	IGHG2_HUMAN	IGHG2	Isoform 1 of Immunoglobulin heavy	1,61E+10	8,76E+09	2,32E+10	8,18E+09	1,51E+10	1,73E+10	2,06E+10	7,09E+09	8,87E+09	1,91E+10	1,32E+10	8,08E+09	1,31E+10	1E+10	1,02E+10	1,3E+10
A0A9H3ZR93	A0A9H3ZR93_HUMAN	IGHG3	Immunoglobulin heavy constant ga	1,47E+10	1,39E+10	1,37E+10	1,47E+10	9,69E+09	1,22E+10	1,18E+10	2,4E+10	3,37E+09	9,41E+09	1,3E+10	1,2E+10	1,06E+10	1,48E+10	9,92E+09	8,98E+09
P01861	IGHG4_HUMAN	IGHG4	Immunoglobulin heavy constant ga	4,33E+09	5,02E+08	1,66E+09	1,05E+09	6,01E+08	1,15E+09	3,84E+09	1,17E+09	1,48E+08	4,11E+09	1,38E+09	1,79E+09	1,47E+09	1,9E+09	4,72E+09	4,4E+09
P01871-1	IGHM_HUMAN	IGHM	Isoform 1 of Immunoglobulin heavy	6,2E+09	4,24E+09	3,52E+09	3,39E+09	3,65E+09	3,94E+09	9,48E+09	9,06E+09	7,83E+09	5,41E+09	4,11E+09	7,06E+09	2,36E+09	2,85E+09	3,53E+09	5,11E+09
P23083	HV102_HUMAN	IGHV1-2	Immunoglobulin heavy variable 1-2	1,91E+08	1,46E+08	4,66E+08	75320800	2,83E+08	98602300	2,13E+08	64035700	1,95E+08	1,24E+08	64273700	1,36E+08	2,19E+08	1,16E+08	3,7E+08	1,95E+08
A0A0C4DH33	HV124_HUMAN	IGHV1-24	Immunoglobulin heavy variable 1-2	47174000	46380800	16858600	19314400	27349200	56718600	26158500	28064200	1,01E+08	18002400	30566600	59468400	33310700	81663300	58345200	1,09E+08
A0A0C4DH29	HV103_HUMAN	IGHV1-3	Immunoglobulin heavy variable 1-3	59919700	85537500	57221200	68344100		26797900	65014900	96818200	29991300	23181300	45618200	77751000	61928400	1,02E+08		37963600
A0A0A0MS14	HV145_HUMAN	IGHV1-45	Immunoglobulin heavy variable 1-4	29884000	31572900	14113600	13101900	22143200	37928600	30375500	29852200	10542000	19024100	28223800	17927100	46630100	78844000	14768200	42358200
P01743	HV146_HUMAN	IGHV1-46	Immunoglobulin heavy variable 1-4	1,65E+08	48206000	66128100	54822000	78677900	47979000	80443900	1,33E+08	81830700	68881300	52786700	63284200	1,57E+08	1,37E+08	85393300	71912700
P01742	HV169_HUMAN	IGHV1-69	Immunoglobulin heavy variable 1-6	2,41E+08	29831600	1,38E+08	73537100	1,66E+08	1,16E+08	1,53E+08	1,07E+08	81316400	1,61E+08	97705700	1,72E+08	2,04E+08	1,31E+08	74255800	1,8E+08
A0A0B4J2H0	HV69D_HUMAN	IGHV1-69D	Immunoglobulin heavy variable 1-6	1829460	25971900	17780500	14610400	12401100	11889300		10316300	13260600	17582600	35247500	15701800	37932300	1,05E+08	12168200	42046600
A0A0B4J2B8	A0A0B4J2B8_HUMAN	IGHV1OR15-9	Immunoglobulin heavy variable 1/(	5928850	4062570	10088800	3362870	2934980	6229580	1283210	3871540	4984190	4269160	1239790	1862010	6091230		7967390	
A0A0B4J1V2	HV226_HUMAN	IGHV2-26	Immunoglobulin heavy variable 2-2	4,07E+08	1,45E+08	58658100	2,49E+08	1,12E+08	1,21E+08	1,33E+08	2,59E+08	2,37E+08	1,64E+08	1,75E+08	1,55E+08	2,25E+08	2,26E+08	1,72E+08	1,8E+08
P01817	HV205_HUMAN	IGHV2-5	Immunoglobulin heavy variable 2-5	97037200	1,61E+08		60672300	42745700	74163800	73408500	92528700	70530500	40789400	68050800	79460800	27995400	87142100	29763100	1,53E+08
A0A0C4DH43	HV70D_HUMAN	IGHV2-70D	Immunoglobulin heavy variable 2-7	3,44E+08	4,72E+08	4,74E+08	1,94E+08	3,53E+08	2,03E+08	1,89E+08	4,32E+08	2,78E+08	2,51E+08	1,28E+08	3,35E+08	1,52E+08	55379400	3E+08	2,06E+08
P01762	HV311_HUMAN	IGHV3-11	Immunoglobulin heavy variable 3-1	6,35E+08	6,99E+08	4,65E+08	3,78E+08	7,72E+08	3,39E+08	7,56E+08	3,41E+08	3E+08	5,43E+08	1,65E+08	5,93E+08	5E+08	7,82E+08	4,47E+08	1,46E+09
P01766	HV313_HUMAN	IGHV3-13	Immunoglobulin heavy variable 3-1	57966300	40800500	50832400	39633600	84305200	37041200	91929200	43597100	52026300	88545300	32929700	86683400	31214800	1,1E+08	65141200	86278800
A0A0B4J1V0	HV315_HUMAN	IGHV3-15	Immunoglobulin heavy variable 3-1	1,12E+08	45492600	73480400	1,01E+08	1,38E+08	60587100	84177800	1,03E+08	92021000	90347800	1,01E+08	93652500	1,03E+08	73533000	53842400	89451500
A0A0C4DH32	HV320_HUMAN	IGHV3-20	Immunoglobulin heavy variable 3-2	20864600	14993300	13290000	7993810	16385600	3134200	20582100	5532610	8082650	9063790	3876370	29422000	12135100	12183800	9323990	32020400
P01764	HV323_HUMAN	IGHV3-23	Immunoglobulin heavy variable 3-2	8,26E+08	5,66E+08	6,68E+08	7,26E+08	7,31E+08	5,62E+08	9E+08	9,3E+08	5,96E+08	5,63E+08	4,14E+08	7,36E+08	7,98E+08	9,16E+08	7,52E+08	4,17E+08

P01768	HV330_HUMAN	IGHV3-30	Immunoglobulin heavy variable 3-3	4010000	1289510	4581030	3663910		3413540		6680300	2201480	3696240	3870670	11553000	4199780	5377950	3391490	2856500
P01772	HV333_HUMAN	IGHV3-33	Immunoglobulin heavy variable 3-3	5,65E+08	4,84E+08	5,6E+08	5,88E+08	3,88E+08	3,17E+08	4,46E+08	7,51E+08	3,65E+08	5,13E+08	6,06E+08	8,1E+08	6,59E+08	9,5E+08	6,84E+08	6,05E+09
A0A0C4DH35	HV335_HUMAN	IGHV3-35	Probable non-functional immunogl	1,97E+08	2,87E+08	2,38E+08	2,85E+08	3,35E+08	4,96E+08	3,51E+08	3,28E+08	2,33E+08	2,76E+08	1,62E+08	3,67E+08	2,88E+08	3,86E+08	2,99E+08	2,66E+08
A0A0C4DH36	HV338_HUMAN	IGHV3-38	Probable non-functional immunogl	1,05E+08	1,08E+08	1,09E+08	66987500	1,46E+08	60441200	99084100	1,82E+08	85634400	1,08E+08	1,39E+08	97185600	2,03E+08	1,27E+08	1,34E+08	96453400
P0DTE1	HV383_HUMAN	IGHV3-38-3	Probable non-functional immunogl	23948500	42894800	14465000	26920100	42226500	26562000	23543500	36368300	51803100	34096800	21357000	27851100	34748000	84171100	44235400	27389700
A0A0B4J1X8	HV343_HUMAN	IGHV3-43	Immunoglobulin heavy variable 3-4	3,17E+08	1,78E+08	2,63E+08	3,55E+08	2,01E+08	2,25E+08	1,55E+08	1,89E+08	2,44E+08	1,73E+08	1,46E+08	3,03E+08	3,03E+08	4,42E+08	2,18E+08	2,74E+08
A0A0A0MS15	HV349_HUMAN	IGHV3-49	Immunoglobulin heavy variable 3-4	4,01E+08	1,11E+08	4,59E+08	1,5E+08	4,65E+08	2,62E+08	6,14E+08	6,99E+08	5,1E+08	50969800	2,02E+08	5,72E+08	3,87E+08	2,76E+08	1,15E+08	3,9E+08
A0A075B6Q5	HV364_HUMAN	IGHV3-64	Immunoglobulin heavy variable 3-6	44544200	1,31E+08	49012200	50605100	49818700	25915000	68936400	63611600	35826300	33705300	43299400	71042500	27708600	28951400	50126400	17037400
A0A0J9YX35	HV64D_HUMAN	IGHV3-64D	Immunoglobulin heavy variable 3-6	1,44E+08	1,42E+08	1,93E+08	2,11E+08	1,94E+08	1,6E+08	3,25E+08	2,15E+08	1,63E+08	1,98E+08	1,5E+08	1,83E+08	1,8E+08	3,01E+08	2E+08	1,88E+08
P01780	HV307_HUMAN	IGHV3-7	Immunoglobulin heavy variable 3-7	1,08E+09	1,12E+09	1,11E+09	6E+08	1,21E+09	1,1E+09	9,16E+08	1,02E+09	8,42E+08	1,17E+09	6,58E+08	8,68E+08	5,99E+08	1,12E+09	1,12E+09	1,32E+09
A0A4W8ZXM2	A0A4W8ZXM2_HUMAN	IGHV3-72	Immunoglobulin heavy variable 3-7	3,07E+09	4,08E+09	3,82E+09	3,86E+09	5,2E+09	4,34E+09	5,5E+09	4,37E+09	4,29E+09	4,51E+09	2,51E+09	3,99E+09	3,33E+09	3,44E+09	3,75E+09	3,7E+09
A0A0B4J1V6	HV373_HUMAN	IGHV3-73	Immunoglobulin heavy variable 3-7	42470100	48006800	25929800	73535300	89795600	36726000	52234700	81194800	1,32E+08	61495000	55721400	71637500	62094300	66094500	1,07E+08	67356000
A0A0B4J1X5	HV374_HUMAN	IGHV3-74	Immunoglobulin heavy variable 3-7	6600000	4775400	5850890	6316160	7449850	3073820	26479100	21568400	6510440	12587500	7100180	13410500	4230140	3536920	8509440	12246100
A0A075B7B8	A0A075B7B8_HUMAN	IGHV3OR16-12	Immunoglobulin heavy variable 3/(	5,86E+08	3,48E+08	3,57E+08	5,42E+08	5,29E+08	6,05E+08	6,88E+08	5,04E+08	6,76E+08	7,57E+08	3,96E+08	6,86E+08	7,21E+08	5,41E+08	5,42E+08	4,62E+08
A0A075B7E8	A0A075B7E8_HUMAN	IGHV3OR16-13	Immunoglobulin heavy variable 3/(	3,17E+08	4,08E+08	4,09E+08	3,73E+08	3,72E+08	4,52E+08	1,16E+09	4,93E+08	2,94E+08	3,65E+08	4,83E+08	3,77E+08	4,96E+08	2,53E+08	3,68E+08	2,11E+08
A0A0C4DH34	HV428_HUMAN	IGHV4-28	Immunoglobulin heavy variable 4-2	1,55E+08	1,42E+08	5,36E+08	1,25E+08	2,66E+08	1,69E+08	1,89E+08	1,4E+08	1,45E+08	1,32E+08	1,14E+08	1,41E+08	1,88E+08	1,67E+08	2,21E+08	2,4E+08
A0A087WSY4	HV432_HUMAN	IGHV4-30-2	Immunoglobulin heavy variable 4-3	1,77E+08	1,81E+08	1,31E+08	78812600	83407700	95809700	35827700	87447000	60197700	77391300	1,01E+08	53012000	92973000	1,88E+08	1,12E+08	76937800
P06331	HV434_HUMAN	IGHV4-34	Immunoglobulin heavy variable 4-3	26696200	25681100	43792600	7735900	23006100	23619600	14441300	15871900	38690800	25871900	12180100	15460800	25573600	71642200	23836000	35736300
P01824	HV439_HUMAN	IGHV4-39	Immunoglobulin heavy variable 4-3	3,59E+08	3,14E+08	1,05E+09	1,08E+08	3,41E+08	93921100	1,35E+09	3,06E+08	2,99E+08	3,08E+08	1,76E+08	3,33E+08	3,76E+08	3,43E+08	4,95E+08	3,79E+08
A0A075B6R2	HV404_HUMAN	IGHV4-4	Immunoglobulin heavy variable 4-4	34342400	21145000	24969500	71398000		20613200	1,39E+08	27134900	41522200	32884400	28346300	23770000	39253800	82874200	22364000	57357900
A0A075B7B6	A0A075B7B6_HUMAN	IGHV4OR15-8	Immunoglobulin heavy variable 4/(	18984700	19825100	12959500	12727700	5073870	10335000	17163400	20824200	21919200	19808800	16813400	9425470	19583000	37406800	21970600	26696500
A0A0J9YXX1	HV5X1_HUMAN	IGHV5-10-1	Immunoglobulin heavy variable 5-1	3,12E+08	39659700	28330300	3,17E+08	1,79E+08	1,18E+08	2,05E+08	4,46E+08	81383000	1,71E+08	2,92E+08	1,49E+08	1,27E+08	4,48E+08	4,54E+08	2,09E+08
A0A0C4DH38	HV551_HUMAN	IGHV5-51	Immunoglobulin heavy variable 5-5	4,51E+08	1,57E+08	1,61E+08	3,57E+08	2,48E+08	2,58E+08	3,3E+08	4,04E+08	1,8E+08	2,83E+08	2,53E+08	2,88E+08	4,5E+08	4,92E+08	3,9E+08	3,42E+08
A0A0B4J1U7	HV601_HUMAN	IGHV6-1	Immunoglobulin heavy variable 6-1	4,09E+08	1,89E+08	2,01E+08	4,01E+08	3E+08	4,15E+08	2,25E+08	5,19E+08	1,04E+08	4,81E+08	2,72E+08	5,2E+08	2,45E+08	3,82E+08	3,03E+08	2,28E+08
P01834	IGKC_HUMAN	IGKC	Immunoglobulin kappa constant	1,82E+10	1,86E+10	8,23E+09	1,63E+10	1,5E+10	1,33E+10	1,35E+10	1,69E+10	1,11E+10	1,57E+10	7,71E+09	8,9E+09	1,48E+10	2,01E+10	1,06E+10	1,66E+10
A0A0A0MT96	A0A0A0MT96_HUMAN	IGKJ3	Immunoglobulin kappa joining 3 (Fi	94996400	85696600	42402000	69624000	68395100	41496800	25512900	1,02E+08	28728800	41414300	52836000	59019000	66300800	43149700	96082100	1,89E+08
A0A0A0MT69	A0A0A0MT69_HUMAN	IGKJ4	Immunoglobulin kappa joining 4 (Fi	57844700	55099900	13464100	27411500	34540700	35980400	47874900	29997800	37817900	28242300	43149100	56842500	38320600	31975100	48473100	48078200
A0A0C4DH73	KV112_HUMAN	IGKV1-12	Immunoglobulin kappa variable 1-:	3,39E+08	3,57E+08	1,45E+08	2,6E+08	3,91E+08	2,73E+08	2,49E+08	3,51E+08	2,03E+08	2,19E+08	1,62E+08	3,08E+08	3,68E+08	4,17E+08	2,53E+08	3,67E+08
A0A0B4J2D9	KV113_HUMAN	IGKV1-13	Immunoglobulin kappa variable 1D	41684500	36848500	2,28E+08	28411700		2,67E+08	6499540	33077900	31686700			46343000	3,92E+08	4,01E+08	61601500	31333200
P04430	KV116_HUMAN	IGKV1-16	Immunoglobulin kappa variable 1-:	45600700	54285500	66903400	72413000	58162100	41224600	76245900	84160400	57627000	56178500	29405100	87854800	92480000	58485300	1,07E+08	69697800
P01599	KV117_HUMAN	IGKV1-17	Immunoglobulin kappa variable 1-:	1,86E+08	85214700	97961400	91734800	2,06E+08	49203900	1,56E+08	1,93E+08	2,35E+08	1,6E+08	1,26E+08	1,87E+08	1,6E+08	1,77E+08	1,74E+08	2,12E+08
A0A075B6S5	KV127_HUMAN	IGKV1-27	Immunoglobulin kappa variable 1-2	1,55E+08	1,47E+08	1,66E+08	1,59E+08	1,54E+08	1,14E+08	1,36E+08	1,86E+08	1,09E+08	1,23E+08	1,11E+08	1,33E+08	2,45E+08	2,19E+08	1,29E+08	1,45E+08
A0AAG2T928	A0AAG2T928_HUMAN	IGKV1-33	Immunoglobulin kappa variable 1-3	2,77E+09	2,05E+09	2,21E+09	2,38E+09	2,71E+09	1,29E+09	2,17E+09	2,29E+09	3,24E+09	2,21E+09	1,79E+09	2,43E+09	2,8E+09	2,91E+09	2,62E+09	4,7E+09
P01602	KV105_HUMAN	IGKV1-5	Immunoglobulin kappa variable 1-5	5,28E+08	6,75E+08	3,39E+08	4,32E+08	5,98E+08	2,55E+08	5,59E+08	4,63E+08	5,62E+08	6,66E+08	4,6E+08	5,71E+08	3,72E+08	3,73E+08	8,36E+08	6,24E+08
A0A0C4DH67	KV108_HUMAN	IGKV1-8	Immunoglobulin kappa variable 1-8	75111400	35885200	23779100	24295100	55286000	37147200	32449600	42701000	30486100	88545500	40063600	46458200	39015300	53542900	17316200	68651700
P01601	KVD16_HUMAN	IGKV1D-16	Immunoglobulin kappa variable 1D	22327700	9685260	12735700	16754400	22721500	18019100	12756900	22009300	6111400	23150800	6740720	26965600	13322000	20188800	32160000	15799300
A0A087WSZ0	KVD08_HUMAN	IGKV1D-8	Immunoglobulin kappa variable 1D	7086450	11473800	29579300	8299590	13058000	8477330	12837800	33023200	4079200	6642900	10864300	7477530		13348900	15104000	10003300
A0A075B6R9	KV224_HUMAN	IGKV2-24	Probable non-functional immunogl	4,3E+08	2,53E+08	7,88E+08	5,16E+08	7,14E+08	2,75E+08	1,04E+09	4,87E+08	4,03E+08	7,72E+08	3,45E+08	4,4E+08	4,59E+08	4,98E+08	8,93E+08	3,61E+08
A0A075B6S2	KV229_HUMAN	IGKV2-29	Immunoglobulin kappa variable 2D	30851200	48387300	35816900	20941900	11252600	58841400	74302400	27713700	17364600	12792900	18021200	25735400	11167400	24887500	34227500	14542600
A0AAG2T961	A0AAG2T961_HUMAN	IGKV2-40	Immunoglobulin kappa variable 2-4	3,7E+09	4,35E+09	2,16E+09	5,48E+09	4,82E+09	4,35E+09	6,14E+09	4,51E+09	2,96E+09	4,36E+09	3,94E+09	2,73E+09	3,94E+09	2,84E+09	4,67E+09	4,36E+09
A0A0A0MRZ8	KV311_HUMAN	IGKV3-11	Immunoglobulin kappa variable 3D	1,71E+09	2,81E+09	2,14E+09	2,58E+09	1,97E+09	2,31E+09	1,98E+09	9,87E+08	2,54E+09	3,18E+09	1,56E+09	1,98E+09	2,8E+09	2,56E+09	2,59E+09	1,66E+09
P01619	KV320_HUMAN	IGKV3-20	Immunoglobulin kappa variable 3-2	4,59E+08	4,07E+08	2,42E+09	1,1E+09	5,04E+08	3,59E+08	3,79E+08	5,03E+08	3,25E+08	4,17E+08	2,53E+08	4,92E+08	5,91E+08	5,07E+08	6,46E+08	3,81E+08
A0A075B6H7	KV37_HUMAN	IGKV3-7	Probable non-functional immunogl	2,49E+09	3,68E+09	2,06E+09	3,14E+09	2,82E+09	2,06E+09	3,67E+09	1,93E+09	2,42E+09	2,51E+09	2,75E+09	2,97E+09	2,99E+09	4,02E+09	3,38E+09	2,79E+09
A0A087WSY6	KVD15_HUMAN	IGKV3D-15	Immunoglobulin kappa variable 3D	3,46E+08	2,8E+08	1,74E+08	2,71E+08	2,57E+08	2,86E+08	2,9E+08	1,46E+08	2,12E+08	2,32E+08	1,45E+08	2,5E+08	3,39E+08	3E+08	3,76E+08	2,24E+08
A0A0C4DH25	KVD20_HUMAN	IGKV3D-20	Immunoglobulin kappa variable 3D	2,94E+08	4,1E+08	2,88E+08	4,59E+08	5,72E+08	2,56E+08	1,35E+09	2,9E+08	5,11E+08	4,7E+08	3,24E+08	2,79E+08	4,16E+08	4,85E+08	4,11E+08	5,33E+08
P06312	KV401_HUMAN	IGKV4-1	Immunoglobulin kappa variable 4-:	1,19E+09	8E+08	6,47E+08	1,14E+09	1,05E+09	5,43E+08	1,78E+09	1,25E+09	1,25E+09	8,3E+08	4,98E+08	1,41E+09	1,41E+09	9,74E+08	1,39E+09	1,2E+09
A0A0C4DH24	KV621_HUMAN	IGKV6-21	Immunoglobulin kappa variable 6-2	41353900	59090900	1,02E+08	1,12E+08	43871700	21905500	44658300	61373300	30754600	25957400	15227800	45626900	97015300	1,59E+08	49474400	36932700
A0A0A0MT36	KVD21_HUMAN	IGKV6D-21	Immunoglobulin kappa variable 6D	13052500	83508100	4562220	8882310	21216700	17067900	5001010	15577200	7192530	14267400	9341720	10670700	3100980	22459200	26948600	53054900
P0DOY3	IGLC3_HUMAN	IGLC3	Immunoglobulin lambda constant :	2,55E+10	2,09E+10	2,2E+10	1,4E+10	2,62E+10	2,45E+10	2,49E+10	3,33E+10	1,54E+10	2,09E+10	2,22E+10	1,79E+10	2,57E+10	2,15E+10	2,15E+10	2,6E+10
P0CF74	IGLC6_HUMAN	IGLC6	Immunoglobulin lambda constant (	2,54E+10	1,64E+10	1,48E+10	1,37E+10	2,11E+10	1,89E+10	2,46E+10	2,74E+10	1,71E+10	1,8E+10	1,85E+10	2,2E+10	2,08E+10	1,84E+10	1,83E+10	2,05E+10
A0M8Q6	IGLC7_HUMAN	IGLC7	Immunoglobulin lambda constant `	3,83E+09	1,79E+09	3,34E+09	3,4E+09	3,07E+09	3,13E+09	1,83E+09	7,82E+09	3,24E+09	2,6E+09	1,92E+09	3,49E+09	3,75E+09	4,26E+09	4,63E+09	5,73E+09
C9JEE0	C9JEE0_HUMAN	IGLL1	Immunoglobulin lambda like polyp	62551700	49487200	11346300	23571000		45961400	87685700	90512300	49020000	49227000	49132900	36259100	69270800	42246400	54669000	73051200
P15814	IGLL1_HUMAN	IGLL1	Immunoglobulin lambda-like polyp	95676500	27806300	15523500	20108600	14970000	32649500	28092400	51701400	12582000	13878900	26939200	18891800	10955600	30018600	19141700	44448000
B9A064	IGLL5_HUMAN	IGLL5	Immunoglobulin lambda-like polyp	3,36E+09	3,53E+09	2,44E+09	1,62E+09	2,44E+09	3,6E+09	2,83E+09	2,57E+09	1,21E+09	1,69E+09	1,25E+09	2,29E+09	2,77E+09	2,45E+09	3,58E+09	3,64E+09
A0A075B6I4	LVX54_HUMAN	IGLV10-54	Immunoglobulin lambda variable 1	10356800	26288700	42679400	12982400			33412400	52428500	58259900	67388100		64730700	14170700	3107700	32212400	6183480
A0A0B4J1U3	LV136_HUMAN	IGLV1-36	Immunoglobulin lambda variable 1	39430900	7339370	6299100	9304750	35942700	33232100	22733200	21512000	8672980	25713800	29203100	15455200	40769300	48523100	24756200	35193900
P01703	LV140_HUMAN	IGLV1-40	Immunoglobulin lambda variable 1	1,58E+08		1,04E+08	58322400	63717300	92775300	1,13E+08	1,56E+08	72798600	58630100	43243000	1,22E+08	1,23E+08	93426000	1,17E+08	1,49E+08
P01699	LV144_HUMAN	IGLV1-44	Immunoglobulin lambda variable 1	7384110	2933150				4398730	4984450	13179800	3148320	2774950	2044470		5398700	2846040	7089270	4089300
P01700	LV147_HUMAN	IGLV1-47	Immunoglobulin lambda variable 1	1,12E+09	9,94E+08	9,29E+08	6,33E+08	8,97E+08	9,38E+08	8,1E+08	2,35E+09	1,11E+09	1,31E+09	6,76E+08	1,21E+09	1,43E+09	1,48E+09	1,1E+09	8,04E+08
P01701	LV151_HUMAN	IGLV1-51	Immunoglobulin lambda variable 1	5,58E+08	2,28E+08	1,68E+08	2,21E+08	3,79E+08	4,59E+08	4,69E+08	8,29E+08	2,79E+08	2,74E+08	2,3E+08	5,65E+08	5,8E+08	4,56E+08	4,22E+08	3,51E+08
P01706	LV211_HUMAN	IGLV2-11	Immunoglobulin lambda variable 2	4,67E+08	5,17E+08	3,81E+08	2,59E+08	6,36E+08	5,85E+08	1,13E+09	1,03E+09	4,02E+08	3,67E+08	4,04E+08	4,79E+08	6,01E+08	4,68E+08	4,61E+08	5,27E+08
P01704	LV214_HUMAN	IGLV2-14	Immunoglobulin lambda variable 2	4,6E+08		1,46E+08	9177610	2,57E+08	2,27E+08	3,85E+08	8,34E+08	2,03E+08		88520400	2,3E+08	2,7E+08		2,81E+08	
A0A075B6J9	LV218_HUMAN	IGLV2-18	Immunoglobulin lambda variable 2	32660500	53915100	62904400	54569000	56373100	75466700	1,56E+08	44001400	73263400	74768100	50888000	68932200	50831100	49018500	82000700	76148500
P01705	LV223_HUMAN	IGLV2-23	Immunoglobulin lambda variable 2	1,44E+08	2,75E+08	1,37E+08	78957300		2,77E+08	1,6E+08	3,01E+08	1,49E+08	1,11E+08	1,28E+08	1,5E+08	2,24E+08	1,67E+08	1,35E+08	2,11E+08
P01709	LV208_HUMAN	IGLV2-8	Immunoglobulin lambda variable 2	61646500	19941100	70304400	79440200	1,59E+08	21434000	1,19E+08	1,85E+08	59166100	25279900	37824700	1,06E+08	76717700	94480400	1,24E+08	1,44E+08
A0A075B6K4	LV310_HUMAN	IGLV3-10	Immunoglobulin lambda variable 3	6,05E+08	4,17E+08	1,88E+08	4,48E+08	8,02E+08	5,06E+08	6,8E+08	3,78E+08	5,45E+08	4,67E+08	6,64E+08	46883100	8,59E+08	8,5E+08	5,07E+08	1,02E+09
A0A075B6K2	LV312_HUMAN	IGLV3-12	Immunoglobulin lambda variable 3	3000320	2379940			3529800	10721800	2957940	3379620	1449810		4446160		3607080	2210620	3094900	1738380
A0A075B6K0	LV316_HUMAN	IGLV3-16	Immunoglobulin lambda variable 3	25418500	24250000	12976500		33887500	44314300	26204300	16839100	12004200	21053300	48570800	18229800	78679200	21555600	22540600	22198800
P01714	LV319_HUMAN	IGLV3-19	Immunoglobulin lambda variable 3	5,08E+08	5,78E+08	3,03E+08	3,13E+08	4,25E+08	5,21E+08	4,58E+08	3,62E+08	7,24E+08	3,45E+08	2,6E+08	4E+08	7,83E+08	6E+08	6,56E+08	8,68E+08
P01718	LV327_HUMAN	IGLV3-27	Immunoglobulin lambda variable 3	4,48E+08	4,29E+08	3,16E+08	2,08E+08	9,68E+08	4,68E+08	5,79E+08	4,87E+08	2,48E+08	5,04E+08	4,54E+08	3,27E+08	4,18E+08	3,81E+08	5,91E+08	7,03E+08
A0A075B6K5	LV39_HUMAN	IGLV3-9	Immunoglobulin lambda variable 3	1,31E+09	7,52E+08	5,16E+08	1,42E+08	8,57E+08	1,04E+09	8,59E+08	1,41E+09	6,1E+08	5,29E+08	6,42E+08	5,86E+08	6,01E+08	1,3E+09	1,08E+09	7,12E+08
A0A075B6I1	LV460_HUMAN	IGLV4-60	Immunoglobulin lambda variable 4	30035800	17123000	52703600	5903930	17773200	3485050	18858800	38271800	26455200	27393500	9555600	23064600	22849800	34381800	47339200	19551700
A0A075B6H9	LV469_HUMAN	IGLV4-69	Immunoglobulin lambda variable 4	1,85E+08	2,29E+08	1,99E+08	64779800	2,34E+08	2,45E+08	1,36E+08	2,11E+08	1,64E+08	1,24E+08	1,07E+08	50841900	1,36E+08	2,32E+08	1,71E+08	2,46E+08
A0A0G2JSC0	A0A0G2JSC0_HUMAN	IGLV5-45	Immunoglobulin lambda variable 5	21966800	5761120	12037100	11605300	33056600	19474100	67551100	21364100	20002900	24268800	43270500	53386900	46444100	17801900	26847800	28877000
P01721	LV657_HUMAN	IGLV6-57	Immunoglobulin lambda variable 6	1,78E+08	29389200	36322700	25975800	35432100	34663500	33522000	86021500	43218900	43705800	29572000	43319100	66012800	1,13E+08	1,08E+08	44612500
P04211	LV743_HUMAN	IGLV7-43	Immunoglobulin lambda variable 7	16086900	2115610	17715500	4788800	14515100	1,01E+08	5894010	67138700	9465540			13341800		20073200	21195200	10385800
A0A075B6I9	LV746_HUMAN	IGLV7-46	Immunoglobulin lambda variable 7	4,4E+08	1,06E+08	3,21E+08	2,56E+08	3,74E+08	4,42E+08	4,75E+08	6,67E+08	3,74E+08	3,1E+08	3,32E+08	3,59E+08	3,79E+08	2,32E+08	6,7E+08	3,12E+08
A0A075B6I0	LV861_HUMAN	IGLV8-61	Immunoglobulin lambda variable 8	4,08E+08	1,85E+08	7,04E+08	4,12E+08		5,66E+08	1,89E+08	5,83E+08	4,21E+08	4,34E+08	2E+08	4,38E+08	3,34E+08	64834400	2,19E+08	4,77E+08
A0A0B4J1Y8	LV949_HUMAN	IGLV9-49	Immunoglobulin lambda variable 9	1,6E+08	1,07E+08	92377700	37514300	51598000		16304200	1,01E+08	1,59E+08	81542600	69609500	1,76E+08	94094100	1,24E+08	1,1E+08	4090240
Q9NPH3	IL1AP_HUMAN	IL1RAP	Interleukin-1 receptor accessory pı	2921950	4586260	6760920		4828620	6769740	7895350	6057950	6727390	7539960	3078920	3401170		5051650	3189990	3365400
O14498	ISLR_HUMAN	ISLR	Immunoglobulin superfamily conta	3076170	752080	2314520	1346440	1993250	2249360	2257900	1682770	981899	994771	1328260	1493430	2006240	2083500	1867730	4462140
P05556	ITB1_HUMAN	ITGB1	Integrin beta-1	512157	1268380	11419700	415601	588867	464539		250829	286699	528777	338660	380040	420835	316131	516091	812770
H7C0N0	H7C0N0_HUMAN	ITIH1	Inter-alpha-trypsin inhibitor heavy	2285670	3150470	2954780	2282960	3152890	2627360	3786770	3557250	2485700	3106140	3742590	2517660	2691840	2652870	2386360	2869420
P19827	ITIH1_HUMAN	ITIH1	Inter-alpha-trypsin inhibitor heavy	1,13E+09	1,68E+09	1,24E+09	1,1E+09	1E+09	9,92E+08	1,46E+09	1,28E+09	1,21E+09	1,75E+09	1,18E+09	1,21E+09	1,12E+09	1,26E+09	9,8E+08	1,12E+09
P19823	ITIH2_HUMAN	ITIH2	Inter-alpha-trypsin inhibitor heavy	1,68E+09	2,61E+09	1,94E+09	1,68E+09	1,57E+09	1,64E+09	2,03E+09	1,7E+09	2,03E+09	2,85E+09	1,44E+09	1,91E+09	1,53E+09	2,02E+09	1,56E+09	1,65E+09
A0A994J439	A0A994J439_HUMAN	ITIH3	Inter-alpha-trypsin inhibitor heavy	1,66E+09	6,56E+08	1,11E+09	1,87E+09	1,71E+09	1,89E+09	1,71E+09	1,31E+09	2,3E+09	9,71E+08	1,41E+09	1,54E+09	1,39E+09	1,35E+09	1,47E+09	1,65E+09
B7ZKJ8	B7ZKJ8_HUMAN	ITIH4	ITIH4 protein	7934090	8034530	18120500	6605640	10714200	10326000	5172490	8044070	22380800	15060400	8940940	14825200	5010480	6062770	23978600	13949600
Q14624	ITIH4_HUMAN	ITIH4	Inter-alpha-trypsin inhibitor heavy	1,36E+09	1,13E+09	1,31E+09	1,46E+09	1,18E+09	1,15E+09	1,1E+09	1,43E+09	1,42E+09	1,27E+09	1,57E+09	1,36E+09	1,16E+09	1,18E+09	1,28E+09	1,37E+09
Q14624-2	ITIH4_HUMAN	ITIH4	Isoform 2 of Inter-alpha-trypsin inh	75116400	82896300	72868600	87944100	80769900	85875000	82098400	1,15E+08	1,14E+08	92335100	1,1E+08	72369000	67477300	71422800	63868500	80683700
Q8WWA0	ITLN1_HUMAN	ITLN1	Intelectin-1	2459750	5168110		2101650	6590820	4946360	2912500	2633230	4352850	5537720	4852640	9792110	6438760	8266280	6636960	9156120
Q9Y287	ITM2B_HUMAN	ITM2B	Integral membrane protein 2B	515103	1148870	1215900	453269	947729	329787	589030	327935	433338	377126	242127	707048	612460	275312	710611	1140260
P01591	IGJ_HUMAN	JCHAIN	Immunoglobulin J chain	5,63E+08	9,07E+08	7,46E+08	9,61E+08	8,19E+08	1,29E+09	1,37E+09	1,58E+09	1,2E+09	1,24E+09	8,85E+08	9,7E+08	1,05E+09	8,32E+08	9,03E+08	9,57E+08
P14923	PLAK_HUMAN	JUP	Junction plakoglobin	85997300	13464300	1,33E+08	29978900	1,75E+08	55637700	46033800	26348000	45837300	41995500	26265700	6220970	42132300	3,65E+08	46607500	51445300
P03952	KLKB1_HUMAN	KLKB1	Plasma kallikrein	65670200	1,5E+08	1,67E+08	1,04E+08	1,29E+08	1,09E+08	1,35E+08	1,34E+08	1,12E+08	1,79E+08	1,36E+08	70292200	96136900	95232800	1,12E+08	1,09E+08
P01042	KNG1_HUMAN	KNG1	Kininogen-1	2,37E+09	2,82E+09	2,51E+09	2,48E+09	2,51E+09	2,33E+09	2,39E+09	2,44E+09	2,27E+09	2,49E+09	2,41E+09	2,38E+09	2,42E+09	2,62E+09	2,11E+09	2,71E+09
P01042-3	KNG1_HUMAN	KNG1	Isoform 3 of Kininogen-1	4,43E+08	4,87E+08	39062300	4,51E+08	4,2E+08	4,13E+08	3,78E+08	3,63E+08	3,17E+08	2,62E+08	3,52E+08	3,75E+08	3,93E+08	3,62E+08	2,12E+08	4,37E+08
Q5T749	KPRP_HUMAN	KPRP	Keratinocyte proline-rich protein	4975930	2360350	3839980	1282930	13500400	1011160	2831750	3718660	2639970	3362640	2226710	1735430	4029340	4105920	3479310	3846410
P04264	K2C1_HUMAN	KRT1	Keratin, type II cytoskeletal 1	2,24E+09	6,38E+08	1,23E+09	1,18E+09	1,94E+09	8,3E+08	9,28E+08	7,33E+08	1,38E+09	8,65E+08	7,96E+08	5,82E+08	8,34E+08	1,26E+09	1,18E+09	1,34E+09
A0A1B0GVI3	A0A1B0GVI3_HUMAN	KRT10	Keratin, type I cytoskeletal 10	1,39E+09	4,32E+08	1,65E+09	6,69E+08	1,46E+09	4,92E+08	7,53E+08	4,93E+08	9,37E+08	4,17E+08	3,29E+08	3,05E+08	6,38E+08	9,93E+08	4,29E+08	1,02E+09
P13646	K1C13_HUMAN	KRT13	Keratin, type I cytoskeletal 13	2,68E+08	95844300	3,06E+08	1,33E+08	3,42E+08	1,05E+08	2,02E+08	94507800	1,52E+08	88770100	78286900	73803700	1,24E+08	1,37E+08	83079600	2,64E+08
P02533	K1C14_HUMAN	KRT14	Keratin, type I cytoskeletal 14	30946500	14087700	52641500	29249500	50485100	27137000	26123200	28725700	18779100	17781100	22114200	20983300	24163200	44017200	32852900	31485900
P08779	K1C16_HUMAN	KRT16	Keratin, type I cytoskeletal 16	7,97E+08	3,8E+08	9,29E+08	4,66E+08	8,95E+08	1,01E+09	4,53E+08	7,77E+08	5,79E+08	4,07E+08	3,86E+08	5,1E+08	3,78E+08	1,92E+09	1,22E+09	6,59E+08
Q04695	K1C17_HUMAN	KRT17	Keratin, type I cytoskeletal 17	39312200	12435600	47810200	27533100	40918000	28139300	26053700	22970200	26206400	13678900	15412100	16441600	13740800	1,37E+08	42155600	28610000
P35908	K22E_HUMAN	KRT2	Keratin, type II cytoskeletal 2 epide	1,36E+09	4,3E+08	1,08E+09	1,01E+09	2,3E+09	7,29E+08	7,53E+08	4,78E+08	2,37E+09	5,92E+08	4,76E+08	3,18E+08	5,32E+08	1E+09	4,18E+08	1,67E+09
P12035	K2C3_HUMAN	KRT3	Keratin, type II cytoskeletal 3	1,3E+08	40356000	1,82E+08	76877600	2,67E+08	71073500	98627200	45734200	1,9E+08	51745300	36891000	30337100	57201300	1,09E+08	28441900	1,81E+08
P19013	K2C4_HUMAN	KRT4	Keratin, type II cytoskeletal 4	1,45E+08	41080600	3,29E+08	69388900	2,92E+08	1,01E+08	73251500	69834800	1,56E+08	45673400	53077200	40365400	93350400	3,88E+08	59533900	1,56E+08
P13647	K2C5_HUMAN	KRT5	Keratin, type II cytoskeletal 5	3,48E+08	1,19E+08	5,04E+08	2,11E+08	4,77E+08	2,28E+08	2,42E+08	1,88E+08	2,53E+08	1,53E+08	1,6E+08	1,33E+08	1,9E+08	6,17E+08	2,12E+08	3,32E+08
P02538	K2C6A_HUMAN	KRT6A	Keratin, type II cytoskeletal 6A	11604200	2482790	6663510	4745810	9203540	22222800	4708580	19741900	8564790	4451780	5898270	4269530	2873670	2,1E+08	20649800	12975500
P04259	K2C6B_HUMAN	KRT6B	Keratin, type II cytoskeletal 6B	86561000	45074900	89330300	69555700	1,04E+08	1,79E+08	70902700	1,44E+08	1,05E+08	56632000	50497600	73177400	43642000	3,87E+08	2,79E+08	1,41E+08
Q3SY84	K2C71_HUMAN	KRT71	Keratin, type II cytoskeletal 71	3674900	1700130	6276740	2780050	7971250	2214230	3504350	3376320	4751410	2120950	3205310		3155570	5673100		3517600
Q7Z794	K2C1B_HUMAN	KRT77	Keratin, type II cytoskeletal 1b	1,38E+09	3,95E+08	1,94E+09	6,55E+08	9,39E+08	5,3E+08	6,26E+08	4,94E+08	7,66E+08	3,65E+08	4,22E+08	3,49E+08	6,37E+08	9,24E+08	4,21E+08	9,76E+08
Q8N1N4	K2C78_HUMAN	KRT78	Keratin, type II cytoskeletal 78	8927220	2841410	7873780	3937330	9723040	3401060	5130000	2862420	7236730	3398800	3059700	1836930	4380560	9066270	2993260	6157240
Q6KB66	K2C80_HUMAN	KRT80	Keratin, type II cytoskeletal 80	2067420	343657	2979800	735453	2130830	1123560	793324	868300	1223990	462433	546803	540035	569378	4918610		1140090
P78386	KRT85_HUMAN	KRT85	Keratin, type II cuticular Hb5	496495	381206	180718	301330	301125	339860	369571	260580	348876	349742	266086	292403	220179	283333	326410	340512
P35527	K1C9_HUMAN	KRT9	Keratin, type I cytoskeletal 9	4,18E+08	2,01E+08	2,38E+08	3,54E+08	4,81E+08	3,32E+08	2,44E+08	2,02E+08	2,31E+08	2E+08	3,81E+08	1,97E+08	2,22E+08	1,74E+08	4,68E+08	2,86E+08
P11279	LAMP1_HUMAN	LAMP1	Lysosome-associated membrane g	1647770	2274100	5739040	952203	2064830	1388880	1553000	1059730	1657620	2302280	1298220	1141980	1045070	2373230		1553090
P13473	LAMP2_HUMAN	LAMP2	Lysosome-associated membrane g	12330000	18627800	30963200	12580700	19085800	11396700	13574400	10715800	14971500	18288800	14320500	12783400	13419400	13984000	15646200	20143600
P18428	LBP_HUMAN	LBP	Lipopolysaccharide-binding protei	1,35E+08	1,18E+08	1,33E+08	1,7E+08	1,44E+08	1,3E+08	1,54E+08	1,72E+08	2,12E+08	2,28E+08	1,19E+08	3,17E+08	1,23E+08	1,25E+08	1,49E+08	1,64E+08
P04180	LCAT_HUMAN	LCAT	Phosphatidylcholine-sterol acyltra	37596300	54525500	34815900	28880900	29643500	40333300	37905000	48732700	38097500	51366300	39723800	34132300	31157300	32966500	39808700	42915600
P13796	PLSL_HUMAN	LCP1	Plastin-2	42892100	48900000	2,69E+08	27381200	45833200	38070500	90665900	40328400	56523800	54128900	51284700	60707300	43066100	50578800	35351300	48379200
P00338	LDHA_HUMAN	LDHA	L-lactate dehydrogenase A chain	4276010	5850300	1,13E+08	1708570	4332110	4835160	10054500	2514210	7797430	7974680	4594330	7157630	3533380	16814000	2900510	3392790
P07195	LDHB_HUMAN	LDHB	L-lactate dehydrogenase B chain	21860800	8532050	2,77E+08	5089460	8504500	7915530	10598700	5607830	53251300	46534500	6179580	12764700	9083000	9095090	6909860	6838470
P09382	LEG1_HUMAN	LGALS1	Galectin-1	192107	57419.9	1895340	151443	227034	163415	305661	187317		99369		231990	162915	221437		196389
Q08380	LG3BP_HUMAN	LGALS3BP	Galectin-3-binding protein	54535300	1,73E+08	68196300	1,56E+08	1,66E+08	1,19E+08	55310100	74613100	1,29E+08	89911700	66994800	94047200	92495400	1,1E+08	88237600	1,28E+08
Q8N6C8	LIRA3_HUMAN	LILRA3	Leukocyte immunoglobulin-like rec	810788		2415820	2903660	2480150	2146650	2583860	3302090	3221160			1423090	1231420	2852480	2954040	2637380
P08519	APOA_HUMAN	LPA	Apolipoprotein(a)	2,65E+08	44105300	63553000	31551100	4,65E+08	2,24E+08	3,55E+08	34872800	6,8E+08	88767300	3,74E+08	1,07E+08	49433000	4,58E+08	1,6E+08	5E+08
P02750	A2GL_HUMAN	LRG1	Leucine-rich alpha-2-glycoprotein	6,01E+08	2,75E+08	3,65E+08	5,2E+08	5,08E+08	4,83E+08	3,73E+08	4,65E+08	4,58E+08	4,74E+08	5,94E+08	6,3E+08	4,43E+08	3,63E+08	6,11E+08	6,09E+08
Q07954	LRP1_HUMAN	LRP1	Prolow-density lipoprotein recepto	3495850	5965000	4394730	4642240	3374660	4314520	7108780	4336850	5099640		3363750	5441810	5548710	6429870	3722030	6716300
P09960	LKHA4_HUMAN	LTA4H	Leukotriene A-4 hydrolase	692704	527359	10642300		718912	696731	2098090	659156	1579180	1231570		633131		2078040	234723	830361

Q14766	LTBP1_HUMAN	LTBP1	Latent-transforming growth factor I	3362890	6070590	53976400	3723880	2750620	2180990	3162490	4455360	4048880	8369070	5767610	3480500	1322280	3387170	2650320	3136850
P02788	TRFL_HUMAN	LTF	Lactotransferrin	11920100	6766820	1,34E+08	9921500	21802400	6433670	29534800	6876520	10677500	24104400	12213600	12803600	10366000	4645020	17117700	8303930
P51884	LUM_HUMAN	LUM	Lumican	3,72E+08	2,48E+08	1,97E+08	2,86E+08	5,04E+08	3,61E+08	1,43E+08	2,15E+08	2,22E+08	1,74E+08	1,79E+08	2E+08	4,01E+08	4,15E+08	4,21E+08	4,79E+08
Q9Y5Y7	LYVE1_HUMAN	LYVE1	Lymphatic vessel endothelial hyalu	5569960	14990200	16080600	10201700	8159640	12484400	8012530	9962020	5534260	7602360	7761990	11702300	15963400	12117400	14821000	21348800
P61626	LYSC_HUMAN	LYZ	Lysozyme C	87415800	44420900	1,74E+08	73449900	81114900	65099900	64574400	80068400	46351100	41045200	3,59E+08	31423800	65850300	43499100	37775200	94980900
P33908	MA1A1_HUMAN	MAN1A1	Mannosyl-oligosaccharide 1,2-alph	5852690	5819230	3635530	5234570	4599090	4487460	6374090	4958180	6814560	9143230	5529850	6638260	5615080	5596340	5787080	6570580
Q16706	MA2A1_HUMAN	MAN2A1	Alpha-mannosidase 2	1086620	1360080	1324780	1348690	1199770	1174210	1044410	1124530	1048080	1494380	1547350	1242040		1074600	888275	1262560
P48740	MASP1_HUMAN	MASP1	Man-binding lectin serine protease	20509000	28766500	24576700	16989900	24330300	19339300	21332800	24964500	23874200	28148900	25232800	23897000	25537300	28297900	22134900	24104000
P48740-2	MASP1_HUMAN	MASP1	Isoform 2 of Man-binding lectin ser	6349090	7415560	5770700	6195240	14786200	6295820	5768900	9729500	8155970	9732680	8584120	7698180	9104340	12648700	6063600	11680900
O00187	MASP2_HUMAN	MASP2	Man-binding lectin serine protease	7392870	11209100	7552750	9825160	5735830	7517590	10407200	8496510	10025900	22317700	16316800	8900440	7817130	5806440	8520460	8177220
P02144	MYG_HUMAN	MB	Myoglobin	2797230		635144	1506370	1733720	3387620	501799	554512		3013310	1199610	4160960	3971420		1438840	3432480
P11226	MBL2_HUMAN	MBL2	Mannose-binding protein C	45826400	1026090	21617200	61585300	9789030	3731440	14994700	16727600	7644200	40550600	21918600	6888220	40279900	15126000	11321200	6769520
P43121	MUC18_HUMAN	MCAM	Cell surface glycoprotein MUC18	4031990	4355560	4270120	3600390	7962870	7554200	3800110	3026590	1998590	2213780	3435550	4492180	6091700	6601930	3616880	6852040
Q7Z7M0	MEGF8_HUMAN	MEGF8	Multiple epidermal growth factor-like domains		2415230		2248380	2975470	1689030	1927340	1266200	1206810	3502690	2408700	2742560	2451990		1725870	3052690
Q9BUN1	MENT_HUMAN	MENT	Protein MENT	324909	579711	292397	237541	241773	376542	165555	358865	412315	392657	411959	274912	218025	306109	297144	257322
Q13361	MFAP5_HUMAN	MFAP5	Microfibrillar-associated protein 5	132057	100225		159585	153688		331486	159205			230271	178035	180241	136629	214326	307211
Q9UNW1	MINP1_HUMAN	MINPP1	Multiple inositol polyphosphate ph	6776860	6842760	20111300	2696370	4145840	4453000	5518490	4316250	3652830	6086260	4891320	5236350	5677050	6533890	4076400	3502580
P08253	MMP2_HUMAN	MMP2	72 kDa type IV collagenase	3696310	1903560	2451970	2322600	5888520	5083510	4166450	1420120	965821	934070	3124730	2995270	5028920	5904840	2201380	5775700
P14780	MMP9_HUMAN	MMP9	Matrix metalloproteinase-9	2968510	1387530	18108000	6941170	7176180	2353950	6387360	1370530	3242590	12238800	3803340	5929380	2036930	272015	5107090	2143950
Q13201	MMRN1_HUMAN	MMRN1	Multimerin-1	4164230	6509810	57983500	3680220	4132230	1805320	3191580	3343580	2426070	4919410	5581560	2325780	2706800	2322320	4426480	4593740
Q9H8L6	MMRN2_HUMAN	MMRN2	Multimerin-2	5106220	5005320	7328340	5680780	4657320	5934880	6054680	3788600	3876790	3921760	5530700	4175370	4506500	7363700	4928610	5191500
P05164	PERM_HUMAN	MPO	Myeloperoxidase	7646150	3124340	1,68E+08	8985120	12493600	4299780	31232800	4761620	5060990	14564800	5452970	7482570	6755580		7607000	6410830
P26927	HGFL_HUMAN	MST1	Hepatocyte growth factor-like prot	28138600	18123700	36895900	12135700	21667300	35808700	26530600	30343800	27944900	17996700	41467500	11884400	22323100	18824200	16809000	12542400
P41271	NBL1_HUMAN	NBL1	Neuroblastoma suppressor of tum	136184		27883.8	127134	195377	109601	119994	98342.1		24377	57602	82278.6	180832	42214.1	55212	257388
P13591	NCAM1_HUMAN	NCAM1	Neural cell adhesion molecule 1	1,66E+08	89517700	85828400	62466400	1,8E+08	1,62E+08	73521800	85837600	67377100	1,37E+08	81855200	1,8E+08	1,37E+08	89187400	1,27E+08	1,37E+08
P14543	NID1_HUMAN	NID1	Nidogen-1	1547470	1809630	6818510	1639520	1311560	1600430	1028840	1687890	2312100	2190990	2226820	2484150	1082530	1819510	2608080	1619220
Q04721	NOTC2_HUMAN	NOTCH2	Neurogenic locus notch homolog p	95485,9			78471.8	73420.3	107064	50922.4	74920.7	60448.9		61463.2	47224.4		45166.5	63794.4	86859.7
Q9UM47	NOTC3_HUMAN	NOTCH3	Neurogenic locus notch homolog p	330970	381162	269177	250860	504874	434604	351340	245834	144470	168163	237331	210071	365337	542251	368624	516840
O14786	NRP1_HUMAN	NRP1	Neuropilin-1	7840450	3153570	6518480	7216250	10440200	8751720	4177680	7558150	4640070	4066120	7589990	6187980	6767730	8482340	10259000	10449200
Q86UD1	OAF_HUMAN	OAF	Out at first protein homolog	1835020	1364600		1911840	1285060	1916420	1681740	1933370	1706360	1272680	648585	1616220	2398040	1743530	657713	2347730
Q99784	NOE1_HUMAN	OLFM1	Noelin	8748890	11479800	11185900	10443900	28481900	9056250	7446170	6443000	6807220	10312800	8396040	8376740	12258200	13948100	19443900	18081100
Q96CV9	OPTN_HUMAN	OPTN	Optineurin	2,15E+08	1,08E+08	1,33E+08	97713500	1,26E+08		3,79E+08	1,53E+08	61176500		1,73E+08	1,63E+08	2,93E+08	1,19E+08	97985300	3,5E+08
P02763	A1AG1_HUMAN	ORM1	Alpha-1-acid glycoprotein 1	1,22E+10	6,94E+09	1,18E+10	8,18E+09	1,05E+10	7,56E+09	7,83E+09	7,56E+09	1,08E+10	1,04E+10	1,05E+10	7,87E+09	7,02E+09	5,77E+09	9,72E+09	1,23E+10
P19652	A1AG2_HUMAN	ORM2	Alpha-1-acid glycoprotein 2	2,99E+09	1,6E+09	1,57E+09	2,76E+09	2,61E+09	2,67E+09	2,6E+09	2,11E+09	2,49E+09	3,13E+09	3,48E+09	2,94E+09	2,84E+09	2,24E+09	2,24E+09	3,07E+09
Q15113	PCOC1_HUMAN	PCOLCE	Procollagen C-endopeptidase enh	10265500	7576650	4675180	9519820	6102400	6025540	11223100	5942160	6266580	2698420	13175300	12507500	7211120	3686720	6252140	7815820
Q8NBP7	PCSK9_HUMAN	PCSK9	Proprotein convertase subtilisin/ke	6065890	10583200	3812300	5515960	4113500	6164660	6098140	4284820	8333850	6653180	8456080	3089830	1892920	5408870	5564690	4690120
Q9UHG3	PCYOX_HUMAN	PCYOX1	Prenylcysteine oxidase 1	34904100	53952300	17308700	31320800	34376100	44233600	29000500	19882700	19736100	42652800	20401800	34565600	16839800	33299000	27397100	45635900
P30101	PDIA3_HUMAN	PDIA3	Protein disulfide-isomerase A3		1612740	1E+08	1233720	2163990		2230330	1200820	1894720	2249830	1977670			8952170	1624840	1946460
Q96S96	PEBP4_HUMAN	PEBP4	Phosphatidylethanolamine-binding protein 4		12244200	19523800	19113700	23871600	21975200	17375500	26882900	14441900	10363700	14143900		37503400	8091650	6701340	27049700
P12955	PEPD_HUMAN	PEPD	Xaa-Pro dipeptidase	5535940	5233910	16189900	3507020	5256110	4003180	4593910	3275940	3446570	5310000	4364560	4453190	3592400	4754060	2775340	4452260
P07737	PROF1_HUMAN	PFN1	Profilin-1	6827740	8711260	2,91E+08	11640300	7650920	3892660	28113400	3697260	7830470	12590000	7367950	6492930	3854150	8344690	3676670	8255110
Q96PD5	PGRP2_HUMAN	PGLYRP2	N-acetylmuramoyl-L-alanine amid	1,75E+08	2,36E+08	1,76E+08	1,8E+08	1,3E+08	1,8E+08	2,05E+08	1,72E+08	1,78E+08	2,08E+08	2,49E+08	1,49E+08	1,54E+08	2,13E+08	1,4E+08	1,6E+08
Q6UXB8	PI16_HUMAN	PI16	Peptidase inhibitor 16	21294700	9483700	16919600	18141400	31675200	29772300	34948700	22910200	19636300	9473540	24209800	22282400	30727800	23493000	27192200	56827400
P01833	PIGR_HUMAN	PIGR	Polymeric immunoglobulin recepto	1,23E+08	1,09E+08	1,35E+08	39702100	1,43E+08	77172300	52349800	1,38E+08	1,33E+08	92378600	1,01E+08	86480400	72825800	1,24E+08	89185400	2,71E+08
P12273	PIP_HUMAN	PIP	Prolactin-inducible protein	13593400	7470340	26812700	6703140	21581100	10128600	7916010	8663400	24584600	10537600	25471400	8580950	11201300	7086200	31455100	11630100
P14618	KPYM_HUMAN	PKM	Pyruvate kinase PKM	1836910	3594800	1,1E+08	937796	3136290	2521840	8349930		8728870	7212780	2397280	1406870	17690000	20702600	2759230	3064070
Q13093	PAFA_HUMAN	PLA2G7	Platelet-activating factor acetylhyd	1269120	1468460	2762060	813947	2379210	2271930	2034860	2216130	1651570	2330140	1749740	2000900	2570950	3386890	1107750	2731780
P00747	PLMN_HUMAN	PLG	Plasminogen	9,83E+08	1,65E+09	1,42E+09	1,15E+09	9,68E+08	1,04E+09	9,57E+08	1,12E+09	8,58E+08	1,19E+09	1,11E+09	9,84E+08	9,13E+08	1,02E+09	1,09E+09	9,47E+08
P55058	PLTP_HUMAN	PLTP	Phospholipid transfer protein	1,77E+08	1,93E+08	2,32E+08	1,31E+08	2,49E+08	2,22E+08	1,84E+08	1,14E+08	1,45E+08	1,48E+08	2,02E+08	1,56E+08	2,37E+08	2,16E+08	1,68E+08	2,93E+08
Q6UX71	PXDC2_HUMAN	PLXDC2	Plexin domain-containing protein 2	11450900	8500680	10092100	6942110	15621700	10969500	9311420	5220880	4620420	5492320	5787720	8674710	8618070	9624460	9325930	14403600
P27169	PON1_HUMAN	PON1	Serum paraoxonase/arylesterase 1	4,89E+08	1,36E+09	7,06E+08	4,36E+08	4,66E+08	3,94E+08	4,47E+08	4,72E+08	8,15E+08	9,27E+08	4,75E+08	4,52E+08	6,83E+08	5,59E+08	3,47E+08	8,23E+08
Q15166	PON3_HUMAN	PON3	Serum paraoxonase/lactonase 3	15104500	43754400	30978100	14021600	17654600	19489100	29940400	26882600	18773400	33410300	29224800	24503800	18895800	19225400	17770500	19407000
P02775	CXCL7_HUMAN	PPBP	Platelet basic protein	57327900	1,51E+08	1,56E+08	67481900	50087000	43839200	65673400	98910600	71891000	99883800	1,41E+08	72379600	27201700	56283600	87658300	54257400
P32119	PRDX2_HUMAN	PRDX2	Peroxiredoxin-2	8910420	17709400	2,47E+09	7167870	18088400	8013650	15150800	12247500	13270000	22984300	8070090	9225630	16668400	22872400	12514400	5023410
P30041	PRDX6_HUMAN	PRDX6	Peroxiredoxin-6	2121890	2926180	5,98E+08	1211000	2299920	1900760	3514470	3265240	1569850	5197460	1748670	2528330	1573530	2593830	3084440	
P13727	PRG2_HUMAN	PRG2	Bone marrow proteoglycan		6743010	3622530	5805010	5442840	8666930	6118020	10331800	4620960	5453960	5841260	5840030	5586780	3519620		4954160
Q92954	PRG4_HUMAN	PRG4	Proteoglycan 4	11267700	21030200	15380700	7789730	7904690	12451700	9172220	7973250	11571100	27793600	22297500	13711200	8781260	10746000	12471100	12999800
P14314	GLU2B_HUMAN	PRKCSH	Glucosidase 2 subunit beta	223184		1800720	162870	246813	173844	188667	108818	112370	83809.8	130042	253767	205654	178954		296982
P04070	PROC_HUMAN	PROC	Vitamin K-dependent protein C	22903000	58662600	31628900	47390300	36767200	54594000	53009400	53389100	47297200	59764200	49293000	38186800	32916000	43592700	39898200	57299000
Q9UNN8	EPCR_HUMAN	PROCR	Endothelial protein C receptor	2815900	3283740	3464700	4099760	6444920	13136800	4686820	3729410	3571380	3075230	4385230	4773190	4812310	10448100	4196460	21636700
P07225	PROS_HUMAN	PROS1	Vitamin K-dependent protein S	1,45E+08	2,3E+08	2,04E+08	2,01E+08	2,22E+08	2,34E+08	1,72E+08	2,07E+08	2,4E+08	2,84E+08	3,02E+08	2,06E+08	1,9E+08	1,95E+08	2,83E+08	2,47E+08
P22891	PROZ_HUMAN	PROZ	Vitamin K-dependent protein Z		25805500	17067000	12571300	15883000	16424200	8190390	18745800	26573500	9043300	18477800	10991500	13514900	11962700	13705200	15164500
P24158	PRTN3_HUMAN	PRTN3	Myeloblastin	2351120		24758100	4765970	3838210	1049980	9454360	1235770	1648560	2620500	1575520	2173490	1989010		3128960	1127220
P07602	SAP_HUMAN	PSAP	Prosaposin	11481100	11987700	48349600	11243400	13035800	18329700	14349300	14344800	8991760	14356900	11732800	13846200	16309900	12453600	13271000	17897200
P41222	PTGDS_HUMAN	PTGDS	Prostaglandin-H2 D-isomerase	37670900	7706190	19224300	31764600	41185100	21743900	26676100	33889700	12206500	9063610	20085800	28605600	43421200	18418100	15333300	61916300
P10586	PTPRF_HUMAN	PTPRF	Receptor-type tyrosine-protein pho	375533	311187	299502	368297	228005	463357	592623		149640	194044	329711	489899	407576	225362	218265	649992
P23470	PTPRG_HUMAN	PTPRG	Receptor-type tyrosine-protein pho	4171900	2468280	1169380	2474250	4339440	4369070	3509770	2732150	2980370	2213910	3566840	3674830	2727420	3153770	2780490	7549260
Q12913	PTPRJ_HUMAN	PTPRJ	Receptor-type tyrosine-protein phosphatase et		2401230	6619610	1494600	1232730		1772680		1551390	1584350	1795610	1371130	1491560	1409880	1342940	2182850
Q13332	PTPRS_HUMAN	PTPRS	Receptor-type tyrosine-protein pho	235590		230211	181306	284862	340145		184192			207223	173671	347134	213976	134256	285102
P15151	PVR_HUMAN	PVR	Poliovirus receptor	3651870	5659000	2164860	3582820	6972920	5783110	6447230	5924680	5367520	3187130	5258100	5183610	5003210	5682490	4482010	7405710
P20742	PZP_HUMAN	PZP	Pregcy zone protein	16133600	5320620		29097700	22938800	6872290	17422000	1,52E+08	5042450	30617400	3083770	3228130	36461600	1385260	1,83E+08	12965400
O00391	QSOX1_HUMAN	QSOX1	Sulfhydryl oxidase 1	26211200	21473100	26523000	21199000	43225900	22323100	21339300	20140000	20759100	20471400	24657000	36896600	31136000	38837200	32941700	32738700
Q99969	RARR2_HUMAN	RARRES2	Retinoic acid receptor responder p	6873780	4179870	6942340	5490800	4412580	3315670	3155760	5563200	4690870	3487180	5636770	2672770	4811420	3686030	4754470	6070730
P02753	RET4_HUMAN	RBP4	Retinol-binding protein 4	1,09E+09	1,73E+09	4,01E+08	1,31E+09	1,11E+09	9,83E+08	1,1E+09	1,39E+09	6,63E+08	8,19E+08	1,11E+09	8,08E+08	9,4E+08	6,23E+08	8,26E+08	1,75E+09
P05451	REG1A_HUMAN	REG1A	Lithostathine-1-alpha	2986390	904891		3421940	2466160	4445430	3551590	5074280	882992	2214230	1958820	2313290	3554010	1562220	357514	4248280
P07998	RNAS1_HUMAN	RNASE1	Ribonuclease pancreatic	17301300	4679040	5800010	19539100	17675400	12928000	10528000	18971100	5716080	3089880	7275010	12492500	18208600	5792810	10190400	25247100
P34096	RNAS4_HUMAN	RNASE4	Ribonuclease 4	5177480	2775960	5497190	5133510	6125250	6939930	4019670	6201540	3495020	2986630	4658600	4801560	5314850	4400870	5306570	5728330
P13489	RINI_HUMAN	RNH1	Ribonuclease inhibitor	1023180	658345	69844900	918868		759918	1391480	483801	1122400	1149920		1615300		4025420	521172	
P26447	S10A4_HUMAN	S100A4	Protein S100-A4	2823490	2488770	4,21E+08	1771760	2119860	1930370	6686140	1920660	1566590	4693620	2326890	3842440	2089180	5286430	2143880	
P31151	S10A7_HUMAN	S100A7	Protein S100-A7	2999890	2478830	12434500	4977570	3978180	4022860	8546380	3801350	2686540	2876350	2517610	5081260	3293450	5138320	41741900	4831030
P05109	S10A8_HUMAN	S100A8	Protein S100-A8	9767160	7557630	6,28E+08	7120790	14991700	11510000	38292700	12298300	9650720	9480180	8116150	9447540	5591150	13103200	19018000	11076300
P06702	S10A9_HUMAN	S100A9	Protein S100-A9	6246820	5792570	5,78E+08	5333550	8137120	7653810	26725300	7260730	5346600	7726220	5942780	6262020	6086290	34195000	13973600	7881820
P0DJI8	SAA1_HUMAN	SAA1	Serum amyloid A-1 protein	60626500	29373600	42194500	38983600	86458000	85660300	43245600	1,1E+08	3,15E+08	1,09E+09	47968500	1,25E+09	1,95E+08	23433200	3,15E+08	66388300
P0DJI9	SAA2_HUMAN	SAA2	Serum amyloid A-2 protein	6174380	3581270		4565960	15315300	10492200		22119700	30142500	1,15E+08	6252000	31506000	7872240		47750400	7802490
A0A096LPE2	A0A096LPE2_HUMAN	SAA2-SAA4	SAA2-SAA4 readthrough	3,52E+08	5,72E+08	3,98E+08	3,41E+08	2,84E+08	2,68E+08	1,54E+08	3,27E+08	5,21E+08	9,02E+08	7,24E+08	3,08E+08	2,56E+08	3,02E+08	3,82E+08	3,62E+08
Q6UWP8	SBSN_HUMAN	SBSN	Suprabasin	6181380	1720820	8237610	2200360	5450020	2367730	4153860	2704660	2009360	1343950	2327060	1498280	2059130	6999660	1480140	5278530
P49908	SEPP1_HUMAN	SELENOP	Selenoprotein P	93970600	1,03E+08	1,39E+08	54848800	1,1E+08	73991600	95410700	65147700	66379400	1,3E+08	97909900	94877200	1,25E+08	86410500	76334900	1,61E+08
P14151	LYAM1_HUMAN	SELL	L-selectin	35588900	38865200	29214700	34148300	45748300	30692500	35673400	46218000	31458000	42568500	35354500	30051100	26358600	35780000	32922400	53460800
P01009	A1AT_HUMAN	SERPINA1	Alpha-1-antitrypsin	1,05E+10	8,73E+09	8,84E+09	9,31E+09	1,04E+10	8,75E+09	7,43E+09	8,98E+09	1,1E+10	9,18E+09	9,29E+09	7,81E+09	8,9E+09	1,02E+10	8,85E+09	7,21E+09
Q9UK55	ZPI_HUMAN	SERPINA10	Protein Z-dependent protease inhil	13377500	35024000	15466200	22259900	21922400	20676200	20873700	19886500	33209100	16414300	30092900	26841200	19821500	18694500	20670000	28059600
Q86U17	SPA11_HUMAN	SERPINA11	Serpin A11	1067940	1038230	1217150	1422170	1116070	1547900	1050240	1101400	1307170	1005280	865854	1189570	822912	976323	1150440	1333470
P01011	AACT_HUMAN	SERPINA3	Alpha-1-antichymotrypsin	2,92E+09	2,11E+09	2,5E+09	3,3E+09	2,56E+09	3,16E+09	2,42E+09	2,74E+09	3,09E+09	3,02E+09	2,65E+09	3,03E+09	2,02E+09	2,31E+09	2,59E+09	2,73E+09
P29622	KAIN_HUMAN	SERPINA4	Kallistatin	1,19E+08	2,14E+08	1,19E+08	1,25E+08	1,39E+08	1,7E+08	1,07E+08	1,23E+08	98597500	1,56E+08	1,38E+08	1,7E+08	1,31E+08	1,48E+08	73169700	1,72E+08
P05154	IPSP_HUMAN	SERPINA5	Plasma serine protease inhibitor	24532000	47508400	1536700	24461000	22016800	32509800	24458900	16094900	9488800	7635130	20209700	23490200	27572000	15047800	10019100	26822300
P08185	CBG_HUMAN	SERPINA6	Corticosteroid-binding globulin	1,82E+08	3,29E+08	2,01E+08	2,51E+08	2,8E+08	2,1E+08	1,95E+08	2,88E+08	2,13E+08	2,56E+08	1,99E+08	1,96E+08	1,77E+08	2,4E+08	2,79E+08	2,24E+08
P05543	THBG_HUMAN	SERPINA7	Thyroxine-binding globulin	1,31E+08	1,7E+08	1,32E+08	1,35E+08	1,41E+08	1,71E+08	84061600	2,14E+08	2E+08	2,3E+08	1,44E+08	2,16E+08	1,35E+08	1,92E+08	1,07E+08	1,4E+08
Q96P63	SPB12_HUMAN	SERPINB12	Serpin B12	2279290	551458	2636120	965964	1999500	671957	1281280	538721	482810		305774		601899	1792970	748549	1169060
P01008	ANT3_HUMAN	SERPINC1	Antithrombin-III	1,93E+09	1,23E+09	1,34E+09	1,16E+09	1,24E+09	1,77E+09	8,48E+08	1,32E+09	1,13E+09	1,07E+09	7,96E+08	1,14E+09	7,35E+08	1,17E+09	1,47E+09	1,44E+09
P05546	HEP2_HUMAN	SERPIND1	Heparin cofactor 2	4,18E+08	1,02E+09	6,69E+08	6,15E+08	6,26E+08	6,45E+08	5,76E+08	8,83E+08	7,77E+08	9,69E+08	8,99E+08	5,71E+08	5,22E+08	5,74E+08	5,17E+08	8,46E+08
P36955	PEDF_HUMAN	SERPINF1	Pigment epithelium-derived factor	1,19E+09	1,17E+09	1,41E+09	1,46E+09	1,19E+09	1,26E+09	1,38E+09	1,33E+09	8,36E+08	1,12E+09	1,43E+09	1,5E+09	1,6E+09	1,06E+09	1,02E+09	1,88E+09
P08697	A2AP_HUMAN	SERPINF2	Alpha-2-antiplasmin	3,09E+08	3,32E+08	5,06E+08	3,64E+08	3,12E+08	3,2E+08	3,04E+08	4,3E+08	3,33E+08	3,81E+08	2,64E+08	3,3E+08	2,41E+08	3,94E+08	2,74E+08	2,87E+08
P05155	IC1_HUMAN	SERPING1	Plasma protease C1 inhibitor	2,3E+09	3,02E+09	1,32E+09	2,47E+09	2,17E+09	2,66E+09	2,07E+09	2,16E+09	2,65E+09	1,98E+09	2,64E+09	2,26E+09	1,76E+09	1,83E+09	1,81E+09	2,67E+09
P07988	PSPB_HUMAN	SFTPB	Pulmonary surfactant-associated p	4501730	2082140	5508060		3004650			7255590	19027500	15663200	6361760	4653440	6729460	5584860		2332800
Q9H299	SH3L3_HUMAN	SH3BGRL3	SH3 domain-binding glutamic acid	2085250	2741120	1,76E+08	3591080	4069730	1098580	5687790	1423020	2387970	5177280	2425130	1748150	1471910	5522770	1183180	4007200
P04278	SHBG_HUMAN	SHBG	Sex hormone-binding globulin	1,13E+08	88060000	1,13E+08	1,5E+08	63044400	1,49E+08	44424100	75192200	88598200	67606000	78108900	61541500	1,1E+08	1,17E+08	1,41E+08	1,88E+08
P02730	B3AT_HUMAN	SLC4A1	Band 3 anion transport protein	828625	1583600	5,2E+09		3070850	1201950		3549990		6795500	3839820		1716780	3364040	2026140	3077130
P08294	SODE_HUMAN	SOD3	Extracellular superoxide dismutase	4710120	2587270	2628060	3799090	4302630	6187710	20671200		1734820	1267290	1941750	14895800	5218270	3008270	2545620	5500280
P09486	SPRC_HUMAN	SPARC	SPARC	31279000	86382500	60903500	36483800	27020700	21258200	31044800	31661500	33982400	56733600	57652500	27333200	19400500	23932100	41300800	23564600
Q14515	SPRL1_HUMAN	SPARCL1	SPARC-like protein 1	6500840	4924540	4707600	5941450	6819970	7982720	5405330	5178020	3521360	5009080	5305010	6744260	6533120	4010440	4336870	11277800
Q13103	SPP24_HUMAN	SPP2	Secreted phosphoprotein 24	15604900	24634000	20999400	19942900	19164600	24350500	14946000	21112900	19244900	16996800	15562300	10985800	16945000	15735900	13042400	10546700
P10124	SRGN_HUMAN	SRGN	Serglycin	13988600	13362800	49098400	13394700	9660520	7071540	7452170	11723800	5974330	15481300	21093800	7921600	7008780	6001380	10341100	10929900
A1L4H1	SRCRL_HUMAN	SSC5D	Soluble scavenger receptor cystein	314816	352410	211266	437604	366467	379800	304446	338949	382430	327762	418814	290620	253166	338195	384627	436308
Q4LDE5	SVEP1_HUMAN	SVEP1	Sushi, von Willebrand factor type A	5088890		1869100	3732840	8940780	3267430	2269890	3420470	2159320		2366830	4338240	5936620	5793390	6880800	7023200
P37802	TAGL2_HUMAN	TAGLN2	Transgelin-2	2930320	4003790	1,17E+08	8281910	6822460		6923440		5190820	11405200	4629420	1954770		4484520	1709410	6778050
P37837	TALDO_HUMAN	TALDO1	Transaldolase	4528770	2244060	1,05E+08	1792000	3305710		7986740	1904100	1826660	3348430		2753230		7256350	2265010	
C9JB55	C9JB55_HUMAN	TF	Transferrin (Fragment)	1,45E+10	1,63E+10	2,06E+10	1,28E+10	1,31E+10	1,43E+10	1,17E+10	1,62E+10	1,06E+10	3,92E+09	1,21E+10	1,09E+10	4,07E+09	1,49E+10	1,37E+10	1,06E+10
C9JVG0	C9JVG0_HUMAN	TF	Transferrin (Fragment)	9E+09	1,27E+10	1,16E+10	9,9E+09	1,01E+10	1,16E+10	1,1E+10	8,71E+09	8,98E+09	1,13E+10	1,21E+10	9,98E+09	1,12E+10	1,2E+10	9,23E+09	9,85E+09
P02787	TRFE_HUMAN	TF	Serotransferrin	1,85E+10	2,1E+10	1,09E+10	1,69E+10	1,66E+10	1,89E+10	1,5E+10	1,49E+10	1,53E+10	1,8E+10	2,06E+10	1,61E+10	1,36E+10	1,85E+10	2,02E+10	1,38E+10
P02786	TFR1_HUMAN	TFRC	Transferrin receptor protein 1	46039100	32283500	44858300	8375370	32525200	15659000	25608900	11662800	20860000	11623300	19568500	25437400	22354800	29936800	26845900	21555600
P01137	TGFB1_HUMAN	TGFB1	Transforming growth factor beta-1	959290	2671230	23043400	1407910	787825	636297	1485250	1681430	1686290	3364010	2619160	1010390	923659	1299280	1276270	780639
Q15582	BGH3_HUMAN	TGFBI	Transforming growth factor-beta-in	21090600	18771100	26578000	7531820	19483200	20294500	15954000	17416700	15987000	27800100	33370600	14626600	15608100	31722600	12992400	23304800
Q08188	TGM3_HUMAN	TGM3	Protein-glutamine gamma-glutamy	2268750	715374	2888710	1093430	2778220	1201710	2796470	728023	943657	918636	770319	552011	1118750	3131210	604348	2175420
O43493	TGON2_HUMAN	TGOLN2	Trans-Golgi network integral memb	5407840	1956370	5035950	5769700	5854360	4576010	5261810	4672380	917855	1834120	2168790	4308790	5523910	2620650	2718070	12651000
P07996	TSP1_HUMAN	THBS1	Thrombospondin-1	73652100	2,39E+08	1,06E+09	1,11E+08	97064200	71591400	1,16E+08	1,21E+08	1,22E+08	1,99E+08	1,64E+08	60454700	66541400	83486500	1,61E+08	91823200
P35443	TSP4_HUMAN	THBS4	Thrombospondin-4	3959520	3826890	5028270	3529140	6729300	7195400	5827330	3938400	2196410	2531310	3663530	4332310	5600330	9805880	5507100	13447400
P35590	TIE1_HUMAN	TIE1	Tyrosine-protein kinase receptor Ti	31974,1	46533.1		59462.2	87559	62500.6	48480.9	67465.2		46458.1	93460.3	51447.4	55959.5	20593.2		
P01033	TIMP1_HUMAN	TIMP1	Metalloproteinase inhibitor 1	18167900	10502300		6967650	11599600	7560820	5169710		7074130	10658400	7145040	7654640	7902970	9026480	10440900	15859900
P16035	TIMP2_HUMAN	TIMP2	Metalloproteinase inhibitor 2	3967190	3414210	4092490	2696450	5964750	4859000	4671250	3266210	2372950	1921860	3693400	4389320	6067590	5450550	4252120	6588190
P29401	TKT_HUMAN	TKT	Transketolase	1318720	900556	30085200	590028	1407930	822930	4184660	643653	771951	1178110	979685	2276410	660031	2813120	803102	692897

P24821	TENA_HUMAN	TNC	Tenascin	1029300	1541910		1186550	1876210	1109210	1143760	1094880	1123850	1191020	1102100	1429120	1425600	1377940	3657900	
P22105	TENX_HUMAN	TNXB	Tenascin-X	2533850	2278730	7637840	2447340	3450350	3338360	3279590	1798510	2711470	2977440	3238720	2722610	2187320	4629740	3646160	2777040
P60174	TPIS_HUMAN	TPI1	Triosephosphate isomerase	4346160	1838140	1,68E+08	2059230	2859380	1524840	5736760	1753200	1512910	3129690	1387970	2056720	8221460	18106900	2871840	2656220
P06753-2	TPM3_HUMAN	TPM3	Isoform 2 of Tropomyosin alpha-3 c	3723110	6193130	8,54E+08	7387870	7084930	4234960	8421050		9293950	9897910	7839650		3083080	14403100	4106560	8610890
P67936	TPM4_HUMAN	TPM4	Tropomyosin alpha-4 chain	461120	452230	1,41E+08	602088	671841		666931		401782	988003	753950		153755	783384	233971	677758
P02766	TTHY_HUMAN	TTR	Transthyretin	2,86E+08	9,49E+08	5,63E+08	3,27E+08	2,5E+08	3,64E+08	4,25E+08	3,53E+08	4,03E+08	3,12E+08	2,72E+08	3,82E+08	3,94E+08	4,13E+08	3,82E+08	4,25E+08
P10599	THIO_HUMAN	TXN	Thioredoxin	34201300	10375300	3,65E+08	23331400	27092700	13676700	30249600	8589850	17161700	17216300	17388500	6317510	11122200	46594800	21057600	28493000
Q6EMK4	VASN_HUMAN	VASN	Vasorin	7029920	4496990	6241450	6332010	9341920	8515280	7495040	5232780	6127020	5666570	7238070	7582810	8277490	6648540	6921480	12642100
P19320	VCAM1_HUMAN	VCAM1	Vascular cell adhesion protein 1	28512000	11130700	15103500	22108200	38240100	25921200	30180900	27523800	17836400	14897500	14661100	19500600	27216100	27199700	22241600	41405800
P18206	VINC_HUMAN	VCL	Vinculin	3262350	2088520	1,58E+08	1500770	3882910	1962690	3841910		3319240	3202850	3159140	2387830	1952120	2637460	2530850	2987510
O95497	VNN1_HUMAN	VNN1	Pantetheinase	2004050	4536810	15019800	1685980	12302600	5102150	5428200	9187730	5066310	15579100	3337250	3374310	5693560	7645070	10363400	2594440
Q9Y279	VSIG4_HUMAN	VSIG4	V-set and immunoglobulin domain-	464788			495538	330544	288178	203447	277367	103403	56500.7	122963	362032	365020	130621	162653	548157
P04004	VTNC_HUMAN	VTN	Vitronectin	9,94E+08	1,59E+09	1,16E+09	1,51E+09	1,14E+09	1,22E+09	1,26E+09	1,47E+09	1,7E+09	2,13E+09	1,49E+09	1,28E+09	9,95E+08	1,39E+09	1,09E+09	1,02E+09
P04275	VWF_HUMAN	VWF	von Willebrand factor	1,98E+08	81997500	1,69E+08	2,99E+08	2,15E+08	2,49E+08	1,37E+08	1,07E+08	1,14E+08	1,8E+08	2,13E+08	1,87E+08	2E+08	2,21E+08	1,08E+08	1,54E+08
P62258	1433E_HUMAN	YWHAE	14-3-3 protein epsilon	6290370	7103910	5,7E+08	4101690	7198290	5806330	14418800	11801200	7102560	7275470	6004250	8432480	6206820	27281400	9604460	13240600
P63104	1433Z_HUMAN	YWHAZ	14-3-3 protein zeta/delta	2621800	3586090	3,62E+08	1229890	3162850	1533840	5248070	2042070	2701560	3617600	3868660	3119750	2397090	10409200	2123830	2638440
A0A0G2JRQ6	A0A0G2JRQ6_HUMAN		Ig-like domain-containing protein (I	1,13E+08	1,67E+08	1,52E+08	1,79E+08	1,26E+08	1,26E+08	2,32E+08	1,7E+08	59504100	1,71E+08	80911700	1,13E+08	1,81E+08	2,08E+08	1,64E+08	1,09E+08
A0A0J9YY99	A0A0J9YY99_HUMAN		Ig-like domain-containing protein (I	4,6E+08	96993300	60582800	4,58E+08	4,5E+08	18029600	3,92E+08	59963200	11464700	44695400	21663200	41281900	9019060	14892200	15648600	36144700
A0A8I5KW61	A0A8I5KW61_HUMAN		C-X-C motif chemokine	3,76E+08	1,03E+09	1,02E+09	4,84E+08	2,93E+08	2,86E+08	3,33E+08	5,11E+08	4,82E+08	8,26E+08	7,38E+08	2,94E+08	1,44E+08	3,7E+08	6,44E+08	3,65E+08
A0A8Q3SIA1	A0A8Q3SIA1_HUMAN		Sushi domain-containing protein	9,85E+08	1,16E+09	9,54E+08	1,05E+09	1,05E+09	8,37E+08	9,6E+08	9,63E+08	1,03E+09	1,39E+09	1,2E+09	1,01E+09	9,7E+08	1,26E+09	1,17E+09	1,09E+09
A0AAG2SXZ4	A0AAG2SXZ4_HUMAN		Ig-like domain-containing protein	3,98E+08	2,01E+08	2,68E+08	1,04E+08	2,3E+08	2,59E+08	2,32E+08	1,3E+08	1,82E+08	2,06E+08	77866900	3,12E+08	3,78E+08	2,27E+08	1,08E+08	3,51E+08
A0AAG2TVE0	A0AAG2TVE0_HUMAN		Ig-like domain-containing protein	4,39E+08	4,27E+08	1,22E+09	2,19E+08	1,81E+08	76130500	1,36E+09	2,1E+08	1,41E+08	94381500	79457700	2,21E+08	1,89E+08	1,68E+08	3,09E+08	9,56E+08
B4E1Z4	B4E1Z4_HUMAN		Complement factor B	9,9E+08	1,21E+09	1,01E+09	1,04E+09	7,79E+08	9,34E+08	8,86E+08	1,1E+09	1,07E+09	9,86E+08	1,02E+09	8,13E+08	6,57E+08	9,53E+08	1,03E+09	9,26E+08
